# Integrating preclinical and clinical evidence: a meta-analysis of probiotic/synbiotic interventions on metabolic syndrome

**DOI:** 10.3389/fmicb.2026.1995383

**Published:** 2026-10-15

**Authors:** Chunxing Li, XiaoJing Li, Jiaxin Li, Yichao Shi, Lichaoyue Sun, Qin Yang, Zhao Ren

**Affiliations:** 1 Department of Pharmacy, Aerospace Center Hospital, Peking University Aerospace School of Clinical Medicine, Beijing, China; 2 Department of Endocrinology, Aerospace Center Hospital, Peking University Aerospace School of Clinical Medicine, Beijing, China; 3 Department of Respiratory and Critical Care Medicine, Aerospace Center Hospital, Peking University Aerospace School of Clinical Medicine, Beijing, China; 4 Department of Gastroenterology, Aerospace Central Hospital, Peking University Aerospace School of Clinical Medicine, Beijing, China

**Keywords:** adipokines, meta-analysis, metabolic syndrome, probiotic, proinflammatory cytokines

## Abstract

**Background:**

Published meta-analyses present inconsistent findings regarding the effects of probiotics on metabolic syndrome (MetS), with most lacking in-depth mechanistic exploration.

**Objective:**

To systematically assess the efficacy of probiotic/synbiotic interventions for MetS across preclinical and clinical settings, and to explore underlying mechanisms by quantifying inflammatory and metabolic biomarkers.

**Methods:**

We systematically searched PubMed, Embase, the Cochrane Central Register of Controlled Trials, CNKI, and Wanfang Data from inception to June 10, 2025. Both animal and clinical studies were included. For outcomes reported in different units, all values were standardized to a single common unit prior to pooling. Meta-analyses were performed using Stata 15.0 to calculate pooled mean differences (MD) and 95% confidence intervals (CIs). Sensitivity and subgroup analyses were used to assess result stability and heterogeneity.

**Results:**

This meta-analysis of 35 studies (12 clinical trials with 696 MetS patients and 23 animal studies) demonstrated significant metabolic improvements with probiotic/synbiotic interventions. In animal models, these interventions can significantly improve body weight (BW) (MD = –13.27 g, 95%CI: –18.66,–7.88), glucose metabolism (fasting plasma glucose (FPG), MD = −41.05 mg/dL, 95%CI: −46.50, −35.60; HbA1c, MD = –1.17%, 95%CI: −1.50, −0.84), lipid metabolism (TG, MD = –51.38 mg/dL, 95%CI: −71.46, −31.30; TC, MD = –36.66 mg/dL, 95%CI: −50.10, −23.22), inflammatory and metabolic targets (IL-6, MD = –15.07 pg./mL, 95%CI: −18.23, −11.91; Leptin, MD = –20.95 ng/mL, 95%CI: −39.79, −2.11; FFA, MD = –130.36 μmol/L, 95%CI: −191.52, −69.21), as well as liver function indicators in animal models of MetS. In patients, changes in FPG were non-significant (MD = –8.76 mg/dL, 95%CI:–18.29,0.77), whereas significant improvements were observed for BW (MD = –1.06 kg, 95%CI:–1.70,-0.42), waist circumference (MD = –2.12 cm, 95%CI:–3.70,–0.55), and lipid profiles: TG (MD = -20.51 mg/dL, 95%CI:–27.69,–13.34), TC (MD = -12.09 mg/dL, 95%CI:–16.15,–8.04), LDL-C (MD = –10.56 mg/dL, 95%CI:–14.96,–6.17), and HDL-C (MD = 1.97 mg/dL, 95%CI:0.55,3.39). Sensitivity analyses confirmed result stability, with low risk of publication bias.

**Conclusion:**

Probiotic and synbiotic supplementation effectively improves body composition and lipid metabolism in MetS patients and animal models, and improves glucose metabolism in animals. In MetS patients, supplementation reduces HbA1c without altering FPG. These interventions produce favorable changes in adipokines, inflammatory cytokines and metabolic stress markers. Further high-quality, long-term RCTs are required to validate these effects and optimize intervention strategies.

**Systematic review registration:**

https://www.crd.york.ac.uk/PROSPERO/, identifiers CRD420261373917 and CRD420261374049.

## 1 Introduction

Metabolic Syndrome (MetS) is a complex cluster of metabolic disorders characterized by central obesity, dyslipidemia, hypertension, and hyperglycemia. As a strong predictor of type 2 diabetes mellitus and cardiovascular diseases, it has become a major global public health challenge (Dhondge et al., 2024). Its pathophysiological mechanism is intricate, involving multiple interrelated pathways such as insulin resistance, chronic low-grade inflammation, oxidative stress, and intestinal flora dysbiosis (Mostafavi Abdolmaleky and Zhou, 2024; Palma et al., 2022).

In recent years, the crucial role of the intestinal microecology in maintaining metabolic homeostasis has attracted increasing attention. Gut microbiota and their metabolites, such as short-chain fatty acids, play confirmed roles in maintaining host metabolic homeostasis. The microbiota exert these effects mainly by modulating microbial composition and diversity, whereas their metabolites—such as short-chain fatty acids—act directly as signaling molecules that regulate host energy metabolism, immune function, and endocrine system (Kim et al., 2024). The imbalance of intestinal flora is closely associated with the occurrence and progression of MetS (Malnick and Ohayon, 2023). Based on this, probiotics and synbiotics (defined according to the consensus statement of the International Scientific Association for Probiotics and Prebiotics as a mixture comprising live microorganisms and substrates selectively utilized by host microorganisms that confers a health benefit on the host) have emerged as research hotspots in this field as potential intervention strategies targeting intestinal microecology to improve metabolic health (Chen et al., 2024a; Swanson et al., 2020).

Previous systematic reviews and meta-analyses have initially confirmed that probiotic and synbiotic supplements exert positive regulatory effects on multiple clinical indicators in patients with MetS (Hadi et al., 2021; Kaul et al., 2024; Dong et al., 2019; Liu et al., 2025; Companys et al., 2020). Studies have shown that such interventions can effectively reduce body composition indicators including body weight (BW), body mass index (BMI), and waist circumference (WC), while improving lipid profiles (decreasing triglycerides (TG) and total cholesterol (TC), and increasing high-density lipoprotein cholesterol (HDL-C)) and fasting plasma glucose (FPG) levels. Subgroup analyses further suggest that the intervention efficacy may be influenced by age, intervention duration, and region (Liu et al., 2025). For instance, benefits were more pronounced in Asian populations, in those under 50 years, and with interventions <12 weeks (Liu et al., 2025). Another comprehensive evaluation of microbial regulatory therapies has also verified that such treatments can effectively improve fasting blood glucose and fasting insulin levels, thereby alleviating insulin resistance (Kaul et al., 2024).

Existing evidence on the benefits of probiotic/synbiotic interventions in patients with MetS is conflicting. Additionally, several research limitations remain: First, systematic integration and comparison between animal and human studies are lacking. Current meta-analyses focus almost exclusively on human RCTs, ignoring the value of animal models in exploring histological and molecular mechanisms—a key bridge between basic and clinical research. No study has integrated animal and clinical trial results to evaluate their consistency, differences, or interspecies translational value. Second, exploration of potential biological mechanisms is superficial: existing meta-analyses only confirm clinical efficacy and provide general mechanistic descriptions, lacking systematic sorting and quantitative integration of specific biomarker changes, which hinders the identification of core pathways. Third, intervention heterogeneity is prominent: previous studies included diverse probiotic/synbiotic forms (e.g., capsules, powders, fermented foods), and complex food matrix components (e.g., viable bacteria fluctuations, strain interactions, sugar/fat interference) introduce significant heterogeneity, confounding true intervention effects. Restricting to standardized preparations (e.g., capsules, tablets, powders) can reduce confounding and improve result reliability.

To address these gaps, this study aims to: systematically integrate evidence on probiotic/synbiotic interventions for MetS in animals and humans; quantitatively evaluate their effects on MetS indicators (body composition, lipid/glucose metabolism, etc.) in both populations; systematically sort and quantitatively analyze mechanism-related biomarker changes to reveal potential pathways; and compare animal and clinical results to explore interspecies translational value and conclusion consistency. Because pooling heterogenous animal and human data would be statistically inappropriate, animal and human evidence are meta-analyzed separately and then compared qualitatively. This study intends to provide comprehensive, accurate evidence-based data for probiotics/synbiotics as MetS adjuvant therapies and guide future translational and clinical research.

## 2 Methods

This study was conducted in strict accordance with the Preferred Reporting Items for Systematic Reviews and Meta-Analyses (PRISMA) guidelines (Page et al., 2021). The protocols for the systematic reviews of human studies and animal studies were prospectively registered with the International Prospective Register of Systematic Reviews (PROSPERO) under registration numbers CRD420261373917 and CRD420261374049, respectively.

### 2.1 Literature search strategy

Relevant studies published from the inception of databases to 10 June 2025 were systematically retrieved from PubMed, Embase, the Cochrane Central Register of Controlled Trials, China National Knowledge Infrastructure (CNKI), and the Wanfang Data Knowledge Service Platform. The search strategy was constructed by combining MeSH terms and free-text words, covering core concepts including “Probiotics,” “Synbiotics,” “Metabolic Syndrome” and “Randomized controlled study” (RCTs). The specific search terms were detailed in Supplementary material 1.

### 2.2 Study selection and inclusion criteria

#### 2.2.1 Inclusion criteria

Studies meeting the following criteria were included:

The study subjects were animals diagnosed with MetS or human patients who met the diagnostic criteria for MetS. Given regional variations in the diagnostic criteria for MetS, subjects were required to receive a confirmed diagnosis of MetS in accordance with authoritative guidelines, including the Third Report of the National Cholesterol Education Program Adult Treatment Panel III (NCEP-ATP III) (National Cholesterol Education Program (NCEP), 2002), the International Diabetes Federation (IDF) (Grundy et al., 2005; Song and Ji, 2005), or other recognized criteria (Diabetes Branch of Chinese Medical Association, 2021). For the purpose of this study, MetS was defined as the presence of three or more of the following five factors: elevated fasting TG, reduced fasting HDL-C, abdominal obesity, elevated blood pressure, and impaired fasting glucose. For animal studies, we did not directly apply the absolute cutoff values of human diagnostic criteria (e.g., NCEP-ATP III/IDF), given the inherent species differences in anatomy and metabolism. Instead, MetS in animal models (e.g., rats fed a high-fat diet) was confirmed when at least three indicators of the aforementioned indicators showed a statistically significant deviation from matched lean controls (*p* < 0.05), with the specific statistical tests detailed in the Methods. increase compared with the non-model control group.

The efficacy and/or safety of probiotic and synbiotic supplementation were verified in the subjects. Probiotic and synbiotic supplementation was defined as the oral intake of pharmaceutical preparations (e.g., capsules, tablets, liquid formulations) containing at least one probiotic strain with a clearly defined dosage.

At least one key MetS-related indicator was assessed, including anthropometric indicators (BW, WC) or biochemical indicators (lipid/glucose metabolism, liver function). Biomarkers associated with the therapeutic targets of MetS were also included, such as C-reactive protein (CRP), tumor necrosis factor-α (TNF-α), interleukin-6 (IL-6), interleukin-1β (IL-1β), lipopolysaccharide (LPS), free fatty acids (FFA), adiponectin, and leptin.

The eligibility criteria comprised RCTs involving human participants and controlled experimental studies in animal models evaluating the same interventions. Sufficient data were provided to calculate mean difference (MD).

#### 2.2.2 Exclusion criteria

Studies were excluded if they met any of the following criteria:

The subjects met fewer than three of the five diagnostic criteria for MetS (fasting TG, fasting HDL-C, abdominal obesity, blood pressure, fasting glucose).

Probiotic and synbiotic supplementation was administered via dietary products including yogurt, kefir, mousse, milk, or other similar dairy/food formulations.

The study was a review, letter, conference abstract, case report, editorial, or study conducted in pediatric populations, and studies without relevant outcome indicators or extractable data were also excluded.

### 2.3 Data extraction and outcome measures

Two investigators (LCX and RZ) independently extracted data using a pre-designed electronic data extraction form. Any discrepancies arising during the extraction process were resolved by a third independent investigator. The extracted data included the authors, year of publication, study region, clinical trial registration number, sample size, mean age of participants, control and intervention regimens (including probiotic strains and placebo details), baseline characteristics of the study population, and all reported outcome measures.

**Primary outcome measures** included FPG, HbA1c, BW, WC, TC, TG, low-density lipoprotein cholesterol (LDL-C), and HDL-C.

**Secondary outcome measures** comprised fasting insulin, homeostasis model assessment of insulin resistance (HOMA-IR), homeostasis model assessment of β-cell function (HOMA-β), 0-h and 2-h plasma glucose levels during an oral glucose tolerance test (OGTT), CRP, IL-6, IL-1β, TNF-α, LPS, FFA, adipokines, leptin, adiponectin, alanine aminotransferase (ALT), and aspartate aminotransferase (AST).

For studies reporting only pre-intervention and post-intervention data, the MD was directly calculated as *post-intervention mean minus pre-intervention mean,* since data were presented as mean ± standard deviation (SD). The standard deviation of the mean difference (SD_d) was derived from the pre-intervention SD (SD_ pre), post-intervention SD (SD_ post), and the correlation coefficient (*r*) between the two sets of data, using the formula:

$$\mathrm{SD}\_d=\sqrt{\mathrm{SD}\_{\mathrm{pre}}^{2}+\mathrm{SD}\_{\text{post}}^{2}-2\cdot r\cdot \mathrm{SD}\_\mathrm{pre}\cdot \mathrm{SD}\_\text{post}}$$

where r was conservatively assumed to be 0.5 in this study.

When data were reported as median with interquartile range (IQR) or median with minimum/maximum values (range), the median was treated as the mean, and the SD was estimated using the following methods:

- SD estimated from IQR: $\mathrm{SD}\approx \frac{\mathrm{IQR}}{1.35}$
- SD estimated from minimum/maximum range: $\mathrm{SD}\approx \frac{\max-\min}{4}$

Subsequently, the pooled standard deviation (SD_ pooled) between the two groups was calculated as: $\mathrm{SD}\_\text{pooled}=\sqrt{\frac{SD_{1}^{2}(n_{1}-1)+SD_{2}^{2}(n_{2}-1)}{n_{1}+n_{2}-2}}$ ​​.

where n₁ and SD₁ denote the sample size and standard deviation of one group, and n₂ and SD₂ denote those of the other group.

The risk of bias in human clinical trials will be assessed using the Cochrane Risk of Bias Tool (RoB 2.0) (Sterne et al., 2019), which evaluates five core domains: randomization process, deviations from intended interventions, missing outcome data, measurement of outcomes, and selective reporting of results. Each study will be graded as low risk, some concerns, or high risk of bias overall. The risk of bias in animal experiments will be evaluated by the Systematic Review Centre for Laboratory Animal Experimentation (SYRCLE) tool (Hooijmans et al., 2014), covering selection bias, performance bias, detection bias, attrition bias, reporting bias, and other biases, with judgments graded as low, unclear, or high risk. Any disagreements will be resolved by arbitration from a third researcher.

### 2.4 Data analysis and synthesis

Statistical analysis will be performed using Stata 15.0 (Stata Corp, College Station, TX, USA). Stratified meta-analysis will be conducted separately for animal and human data. The mean differences (MD_change_) and their standard deviations (SD_change_) from baseline to the end of the trial for both the intervention and control groups were pooled using the DerSimonian-Laird random-effects model combined with the inverse variance method. For outcomes reported in different units, all measurements were standardized to a common metric before pooling. Blood glucose and blood lipid concentrations (FPG, TC, TG, LDL-C, HDL-C) were converted to mg/dL; serum insulin to pmol/L; free fatty acids (FFA) to μmol/L; and all other continuous outcomes were likewise converted to a single common unit per outcome according to their respective measurement definitions. No studies were excluded because of non-convertible units, as all reported data could be reliably standardized. To avoid unit-of-analysis errors, the shared control group’s sample size (N) was split proportionally across intervention arms, with its mean and SD preserved; each arm was compared against its split control subgroup without combining arms.

Subgroup analyses were performed according to age group, BMI, pro/synbiotic bacterial dosage and trial duration.

Sensitivity analysis was carried out by omitting one study at a time to evaluate the stability of the study results. When the number of included studies was ≥10, publication bias was visually assessed using funnel plots and quantitatively verified via Egger’s test. A two-sided *p* value < 0.05 was considered statistically significant.

## 3 Results

### 3.1 Literature search and study characteristics

The process of literature search and selection is presented in Figure 1. The first search retrieved a total of 4,263 records from PubMed (*n* = 413), Embase (*n* = 2003), Cochrane (*n* = 304), and ClinicalTrials.gov (*n* = 59), Wanfang Database (*n* = 218), CNKI (*n* = 1,266). After deduplication, 3,633 titles and abstracts were screened, and 35 full-text articles were finally included for quantitative synthesis, involving 12 studies (Depommier et al., 2019; Rabiei et al., 2015; Cicero et al., 2021; Eslamparast et al., 2014; Kathiya et al., 2025; Luangphiphat et al., 2025; Gorenjak et al., 2015; Parastouei et al., 2022; Rabiei et al., 2019; Rahimi et al., 2022; Wastyk et al., 2023; Yang J. et al., 2023) enrolling 696 patients with MetS and 23 studies (Yang et al., 2025; Cano et al., 2013; Cavalcante et al., 2019; Chen et al., 2024b; Huang et al., 2021; Jiang et al., 2023; Lee et al., 2018; Lin et al., 2022; Miao et al., 2023; Moya-Pérez et al., 2015; Oh et al., 2019; Osman et al., 2021; Sharma et al., 2020; Silva et al., 2022; Song et al., 2021; Sun et al., 2022; Wu et al., 2023; Yang et al., 2020; Yang M. et al., 2023; Yi et al., 2021; Yu et al., 2013; Zhang et al., 2021; Zheng et al., 2021) using rodent models of MetS (Supplementary material 2).

**Figure 1:**
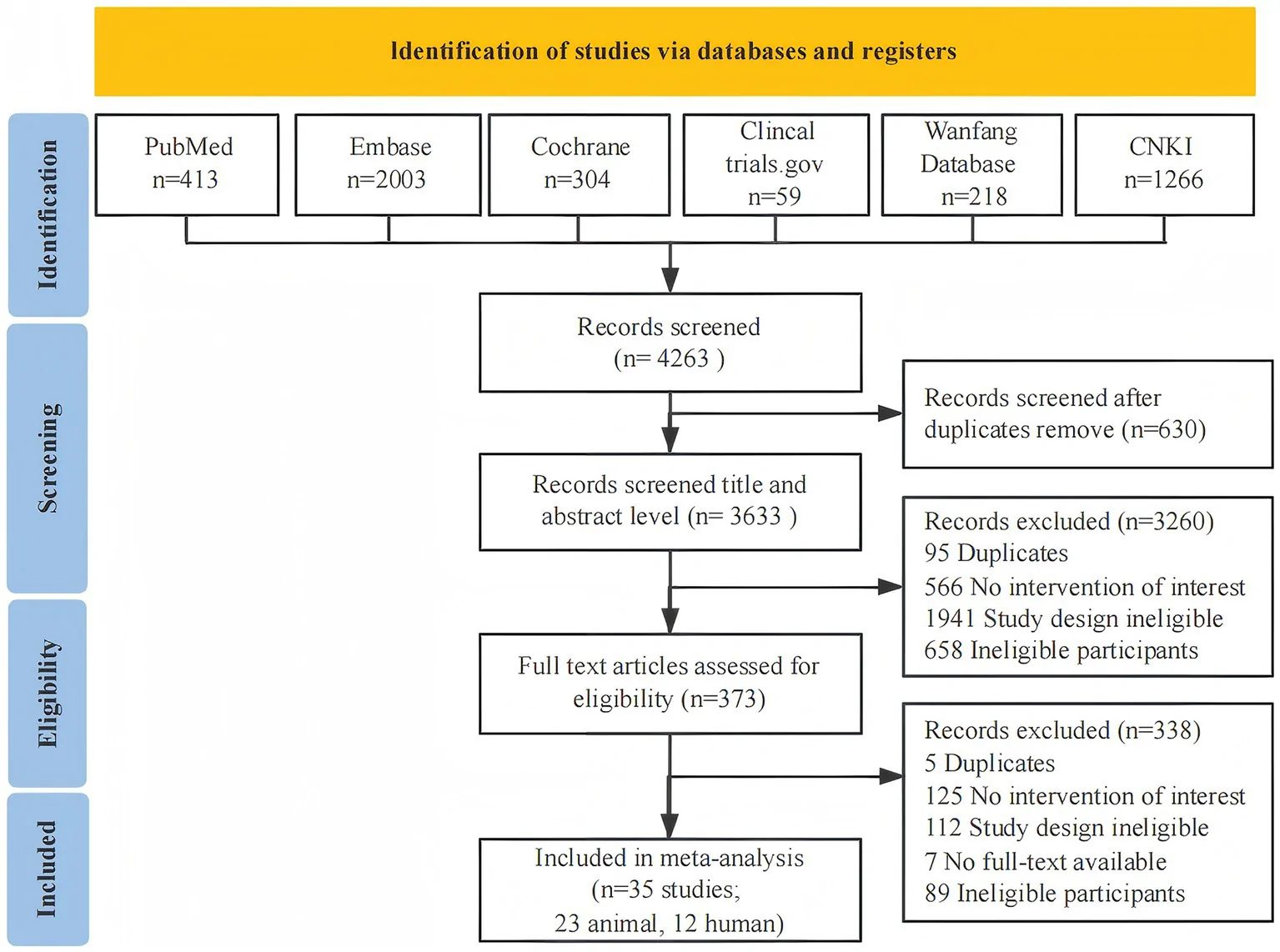
The literature search and selection process.

### 3.2 Trial characteristics and participant demographics

A total of 23 animal studies were included, including 7 in Chinese and 16 in English. The studies spanned six countries/regions, with China contributing the largest proportion (*n* = 13, 56.52%), followed by Korea (*n* = 4, 17.9%). The median duration of the intervention period was 12 weeks (range: 4–28 weeks). Details are shown in Table 1A.

**Table 1:** Characteristics of included studies.

(A) Characteristics of included experimental studies (Animal)										
**Study**	**Duration**	**Outcome**	**Treatment**	**Probiotic dose (CFU/day)**	**Sample size (n)**	**Country**	**Population**	**Age**	**Male (%)**	**Weight (g)**
Zhang et al. (2021)	10 W	FPG/HbA1c/TC/LDL-C/TG/HDL-C/Insulin/OGTT 0 h Blood Glucose/OGTT 2 h Blood Glucose/HOMA-IR/HOMA-β	HFD	NA	8	China	C57BL/6 J rats	6w	100	36.41
			*Bifidobacterium animalis* subsp. *lactis CP-9*	1.0 × 10^9^	8					
			*Lactobacillus reuteri*	1.0 × 10^9^	8					
Osman et al. (2021)	9 W	TC/LDL-C/TG/HDL-C	HFD	NA	8	Egypt	Albino Wistar Rats	2 M	100	160–170
			Postbiotic CFE-1(low dose)	1.0 × 10^8^	8					
			Postbiotic CFE-2(high dose)	2.0 × 10^8^	8					
Yang et al. (2020)	14 W	TC/LDL-C/TG/HDL-C/BW/CRP/FFA/IL-1β/IL-6/LPS/TNF-α	HFFD		10	China	ICR rats	7w	100	35–40
			*Lactobacillus plantarum S2-5*	1.0 × 10^9^	10					NA
			*Lactobacillus plantarum S38*	1.0 × 10^9^	10					
			*Lactobacillus plantarum S39*	1.0 × 10^9^	10					
			*Lactobacillus plantarum S9*	1.0 × 10^9^	10					
			*Lactobacillus plantarum C11*	1.0 × 10^9^	10					
			*Lactobacillus plantarum P3-18*	1.0 × 10^9^	10					
Zhang et al. (2021)	14 W	FPG/TC/LDL-C/TG/HDL-C/BW/Insulin/HOMA-IR/CRP/FFA/IL-1β/IL-6/LPS/TNF-α	HFFD	NA	10	China	ICR rats	7w	100	35 ~ 50
			*Pediococcus acidilactici AS185*	1.0 × 10^9^	10					
Yu et al. (2013)	8 W	FPG/TC/LDL-C/TG/HDL-C/WC/BW/Insulin	HFD	NA	12	China	Sprague–Dawley rats	3w	100	NA
			*Lactobacillus acidophilus 150B*	1.0 × 10^9^	12					50
			*Bifidobacterium breve DM8310*	1.0 × 10^9^	12					232
Yi et al. (2021)	12 W	FPG/TC/TG/BW/Insulin/HOMA-IR	HFD	NA	10	China	C57BL /6 N rats	5w	100	19
			VSL#3(multiple strains)	4.5 × 10^11^	10					
Chen et al. (2024b)	13 W	FPG/HbA1c/TC/LDL-C/TG/HDL-C/Insulin/HOMA-IR/FFA	HFD	NA	10	China	Sprague–Dawley rats	8w	100	170
			*Lactibiane iki*	1.0 × 10^7^	10					
Wu et al. (2023)	14 W	BW	HFD	NA	8	China	Sprague–Dawley rats	5w	100	NA
			*Lacticaseibacillus paracasei LM-141* (low dose)	2 × 10^7^	8					
			*Lacticaseibacillus paracasei LM-141* (high dose)	2 × 10^9^	8					
Cano et al. (2013)	14 W	FPG/TC/TG/Insulin/Leptin	HFD	NA	6	Spain	C57BL-6 mice	6-8w	100	NA
			*Bifidobacterium pseudocatenulatum CECT 7765*	5 × 10^8^	6					
Moya-Pérez et al. (2015)	14 W	FPG/TC/TG/BW/Insulin/ALT/IL-1β/IL-6/TNF-α/Leptin	HFD	NA	10	Spain	C57BL-6 mice	6-8w	100	NA
			*Bifidobacterium pseudocatenulatum CECT 7765*	1.0 × 10^9^	10					
Oh et al. (2019)	16 W	FPG/TC/LDL-C/HDL-C/BW/Insulin/HOMA-IR/IL-1β/TNF-α	HFD	NA	10	Korea	C57BL/6 J mice	6w	100	NA
			*Lactobacillus plantarum PMO 08*	1 × 10^8^	10					
Miao et al. (2023)	18 W	FPG/BW/OGTT 0 h Blood Glucose/OGTT 2 h Blood Glucose	HFD	NA	8	China	C57BL/6 J mice	6w	100	22.28 ± 0.66
			*Lacticaseibacillus paracasei K56* (low dose)	1.0 × 10^7^	8					NA
			*Lacticaseibacillus paracasei K56* (medium dose)	1.0 × 10^8^	8					
			*Lacticaseibacillus paracasei K56* (high dose)	1.0 × 10^9^	8					
Lin et al. (2022)	9 W	TC/LDL-C/TG/HDL-C/ALT/AST	HFD	NA	8	China	C57BL/6 mice	6w	100	NA
			*Lactobacillus delbrueckii subsp. bulgaricus strain TCI904* (low dose)	2.0 × 10^7^	8					
			*Lactobacillus delbrueckii subsp. bulgaricus strain TCI904* (high dose)	1.0 × 10^8^	8					
Lee et al. (2018)	4 W	FPG/LDL-C/TG/HDL-C/Insulin	HFD	NA	5	Korea	C57BL/6 J mice	12w	100	NA
			*Lactobacillus plantarum Ln4*	5.0 × 10^8^	5					
Huang et al. (2021)	12 W	TC/LDL-C/TG/BW/Adiponectin/Leptin	HFD	NA	6	Korea	C57BL/6 J male	5w	100	NA
			*Lactiplantibacillus plantarum KC28*	5.0 × 10^9^	6					
			*Lactobacillus rhamnosus GG*	5.0 × 10^9^	6					
			*Lactiplantibacillus plantarum 299v*	5.0 × 10^9^	6					
Yang M. et al. (2023)	12 W	TC/TG/ALT/AST	HFD	NA	9	Korea	C57BL/6 mice	7w	100	NA
			*Faecalibacterium prausnitzii A2-165*	1.0 × 10^8^	9					
			*Faecalibacterium prausnitzii EB-FPDK3*	1.0 × 10^8^	9					
			*Faecalibacterium prausnitzii EB-FPDK9*	1.0 × 10^8^	9					
			*Faecalibacterium prausnitzii EB-FPDK11*	1.0 × 10^8^	9					
			*Faecalibacterium prausnitzii EB-FPYYK1*	1.0 × 10^8^	9					
Cavalcante et al. (2019)	4 W	TC/LDL-C/TG/HDL-C/BW	HFD	NA	8	Brazil	Wistar rats	90d	100	NA
			*Lactobacillus fermentum 296*	1 × 10^9^	8					
Sharma et al. (2020)	28 W	FPG/TC/LDL-C/TG/HDL-C/BW/Insulin/ALT/AST	HFD	NA	7	India	Sprague Dawley rats	6w	100	140–160
			*Lactobacillus casei*	1.0 × 10^9^	7					NA
			*Lactobacillus gasseri*	1.0 × 10^9^	7					
			*Lactobacillus rhamnosus*	1.0 × 10^9^	7					
Silva et al. (2022)	16 W	FPG/TC/LDL-C/TG/HDL-C/WC/BW/Insulin/HOMA-IR/FFA	HFD	NA	12	Brazil	Wistar Hannover rat	3w	100	NA
			*Bifidobacterium lactis HN019*	1.0 × 10^10^	12					
Jiang et al. (2023)	12 W	FPG/BW	HFD	NA	8	China	C57BL/6 J mice	6w	100	NA
			*Lactobacillus gasseri CKCC1913*	1.0 × 10^9^	8					
Song et al. (2021)	6 W	TC/LDL-C/TG/HDL-C	HFD	NA	10	China	BALB/c mice	5w	100	NA
			*Loigolactobacillus coryniformis* subsp. *torquens T3* (high dose)	1.0 × 10^9^	10					
			*Loigolactobacillus coryniformis* subsp. *torquens T3* (low dose)	1.0 × 10^8^	10					
			*Lacticaseibacillus paracasei* subsp. *paracasei M5* (high dose)	1.0 × 10^9^	10					
			*Lacticaseibacillus paracasei* subsp. *paracasei M5* (low dose)	1.0 × 10^8^	10					
			*Lacticaseibacillus paracasei* subsp. *paracasei X12*(high dose)	1.0 × 10^9^	10					
			*Lacticaseibacillus paracasei* subsp. *paracasei X12*(low dose)	1.0 × 10^8^	10					
Sun et al. (2022)	8 W	IL-1β/IL-6/TNF-α	HFD	NA	8	China	Sprague–Dawley rats		100	180
			*Bacillus natto* (low dose)	1.0 × 10^6^	8					
			*Bacillus natto* (medium dose)	1.0 × 10^8^	8					
			*Bacillus natto* (high dose)	1.0 × 10^10^	8					
Yang et al. (2020)	10 W	FPG/BW	HFD	NA	6	China	C57BL/6 J mice	4w	100	18
			*Lactiplantibacillus plantarum* (high dose)	1.0 × 10^9^	6					
			*Lactiplantibacillus plantarum* (medium dose)	1.0 × 10^8^	6					
			*Lactiplantibacillus plantarum* (low dose)	1.0 × 10^7^	6					
NA: not applicable; HFFD: High Fat-Fructose Diet; HFD: High-Fat Diet; BW: body weight; BMI: body mass index; WC: waist circumference; FPG: Fasting Plasma glucose; HbA1c: Hemoglobin A1c; TC: Total Cholesterol; LDL-C: Low-density lipoprotein cholesterol; TG: Triglycerides; HDL-C: High-density lipoprotein cholesterol; HOMA-IR: Homeostasis model assessment of insulin resistance; HOMA-β: Homeostasis model assessment of β-cell function; OGTT: Oral Glucose Tolerance Test; CRP: C-reactive protein; IL-6: Interleukin-6; IL-1β: Interleukin-1 beta; TNF-α: Tumor Necrosis Factor-alpha; LPS: Lipopolysaccharides; FFA: Free fatty acids; ALT: Alanine aminotransferase activity; AST: Aspartate aminotransferase activity.										

(B) Characteristics of included clinical studies (Human)												
Study	Duration	Trial registration	Outcome	Treatment	Probiotic dose (CFU/day)	Sample size (n)	Country	Population	Age	Male(%)	BMI(kg/m^2^)	Weight (kg)
Yang J. et al. (2023)	12 W	NCT05005754	HbA1c/WC/OGTT 0 h Blood Glucose/OGTT 2 h Blood Glucose/HOMA-IR/HOMA-β/ALT/AST	*Lacticaseibacillus paracasei 8,700:2*	1.0 × 10 ^10^	66	China	MetS	45.89	69.7	26.62	NA
				placebo	NA	64			46.05	76.6	26.82	
Gorenjak et al. (2015)	3 M	NCT02288572	FPG/TC/LDL-C/TG/HDL-C/WC/BWt/Adiponectin	*Lactobacillus plantarum PCS 26*	1.0 × 10^9^	8	Slovenia	MetS	48.3	75	30.3	99.7
				placebo	NA	8				50	32.9	92.75
Luangphiphat et al. (2025)	12 W	TCTR20230505002	FPG/HbA1c/TC/LDL-C/TG/HDL-C/WC/BW/ALT/AST	*Lacticaseibacillus paracasei MSMC39-1* and *Bifidobacterium animalis TA-1*	1.0 × 10^9^	31	Thailand	risk factors of MetS	42.29	30	28.27	76.47
				Placebo	NA	27			43.89	30	27.07	75.9
Kathiya et al. (2025)	12 W	NA	TC/LDL-C/TG/HDL-C/CRP/IL-6/TNF-α	*Lactobacillus* and *Bifidobacterium* species	5.0 × 10^9^	40	India	MetS	30–65	NA	NA	NA
				Control Group		40						
Depommier et al. (2019)	3 M	NCT02637115	HbA1c/TC/LDL-C/TG/WC/BW/Insulin/ALT/AST/CRP/LPS	*Akkermansia muciniphila*	1.0 × 10 ^10^	9	Brussels	excess body BW insulin resistance and a MetS	52.89	67	36.82	109.03
				Placebo	NA	11			49.45	45	37.63	112.45
Rabiei et al. (2015)	3 M	IRCT2013111115368N1	WC	Symbiotic (multiple strains)	4 × 10^8^	23	Iran	MetS	57.1	26.09	32.00	81.3
				Placebo	NA	23			60.8	30.4	32.7	84
Rabiei et al. (2019)	3 M	IRCT2013111115368N1	FPG/TC/LDL-C/TG/HDL-C/BW/Insulin/HOMA-IR	Symbiotic (multiple strains)	4 × 10^8^	23	Iran	MetS	57.1	26.09	32.00	81.3
				Placebo	NA	23			60.8	30.4	32.7	84
Eslamparast et al. (2014)	28 W	NCT02008838	FPG/TC/LDL-C/TG/HDL-C	Symbiotic (multiple strains)	4 × 10^8^	19	Iran	MetS	47.52	39.47	32.13	NA
				Placebo	NA	19			46.05		31.51	
Parastouei et al. (2022)	8 W	NA	FPG/WC/TC/LDL-C/TG/HDL-C	Symbiotic (multiple strains)	1.0 × 10^10^–1.0 × 10^8^	30	Iran	MetS in military personnel	42.33	63.3	32.51	NA
				Placebo	NA	30			40.6	53.3	31.64	
Rahimi et al. (2022)	12 W	IRCT201608299856N3	FPG/TC/LDL-C/TG/HDL-C/WC/BW/Insulin/HOMA-IR/HOMA-β/ALT/AST	Symbiotic (multiple strains)	2.0 × 10^9^	52	Iran	MetS	42.77	42.3	31.03	84.28
				Placebo	NA	56			45.64	35.7	31.14	82.04
Cicero et al. (2021)	74d	NA	FPG/TC/LDL-C/TG/HDL-C/WC/HOMA-IR/ALT/AST/TNF-α/Adiponectin/Leptin	Symbiotic (multiple strains)	6 × 10^9^	30	Italy	Older adults with MetS	72	46.67	27.4	NA
				Placebo	NA	30			71	43.33	27.3	
Wastyk et al. (2023)	10 W	NCT03201068	FPG/TG/HDL-C/WC	*Limosilactobacillus reuteri NCIMB 30242; Lactiplantibacillus plantarum UALp-05; Bifidobacterium animalis* subsp. *lactis B420*	2.0 × 10^10^	21	USA	MetS	57	46	NA	87.2 kg
				placebo	NA	13			55	38		87.1 kg
NA: not applicable; MetS: metabolic syndrome; BW: body weight; BMI: body mass index; WC: waist circumference; FPG: Fasting Plasma glucose; HbA1c: Hemoglobin A1c; TC: Total Cholesterol; LDL-C: Low-density lipoprotein cholesterol; TG: Triglycerides; HDL-C: High-density lipoprotein cholesterol; HOMA-IR: Homeostasis model assessment of insulin resistance; HOMA-β: Homeostasis model assessment of β-cell function; OGTT: Oral Glucose Tolerance Test; CRP: C-reactive protein; IL-6: Interleukin-6; IL-1β: Interleukin-1 beta; TNF-α: Tumor Necrosis Factor-alpha; LPS: Lipopolysaccharides; FFA: Free fatty acids; ALT: Alanine aminotransferase activity; AST: Aspartate aminotransferase activity.												

A total of 12 human studies were included, all of which were published in English. The included studies were conducted in 8 countries/regions, with Iran (5 studies, 41.67%) contributing the most. The proportion of male participants ranged from 26.09 to 46.6%. The median duration of the intervention period was 12 weeks (range: 8–28 weeks). Details are shown in Table 1B.

The publication years of the included studies ranged from 2013 to 2025, with 25 studies (71.43%) published in 2020 or later. Details are shown in Table 1.

### 3.3 SYRCLE risk-of-bias assessment

The SYRCLE risk-of-bias assessment results showed that among the 23 included studies, 5 were rated as low risk and 18 as unclear for sequence generation; all studies were rated as low risk for baseline characteristics; allocation concealment, random housing, and random outcome assessment were all rated as unclear; blinding of caregivers/researchers and blinding of outcome assessor were each rated as low risk in 2 studies and unclear in 21; for incomplete outcome data, selective outcome reporting, and other sources of bias, all studies were rated as low risk. No study was rated as high risk in any domain (Supplementary material 3).

### 3.4 Risk of bias assessment

The risk of bias (ROB) was assessed using the Cochrane Collaboration’s ROB tool. Overall, 41.67% of the included studies were rated as low risk of bias, 58.33% as having some concerns, and 0% as high risk of bias. For randomization sequence generation, 41.67% of studies were at low risk of bias, 58.33% had some concerns, and 0% were at high risk of bias. All studies (100%) were judged to be at low risk of bias for intended interventions, outcome data, outcome assessment and selective reporting (Supplementary material 4).

### 3.5 Effect on glucose metabolism

#### 3.5.1 Change in FPG

Meta-analysis of 14 animal studies involving 308 animals with MetS and 9 clinical studies involving 550 patients with MetS showed that probiotic intervention significantly reduced FPG in animals compared with placebo (MD = −41.05 mg/dL, 95% CI: −46.50 to −35.60, *p* < 0.001) (Figure 2A and Table 2A), but did not reduce FPG in patients (MD = −8.76 mg/dL, 95% CI: −18.29 to 0.77, *p* = 0.07) (Figure 2B and Table 2B).

**Figure 2:**
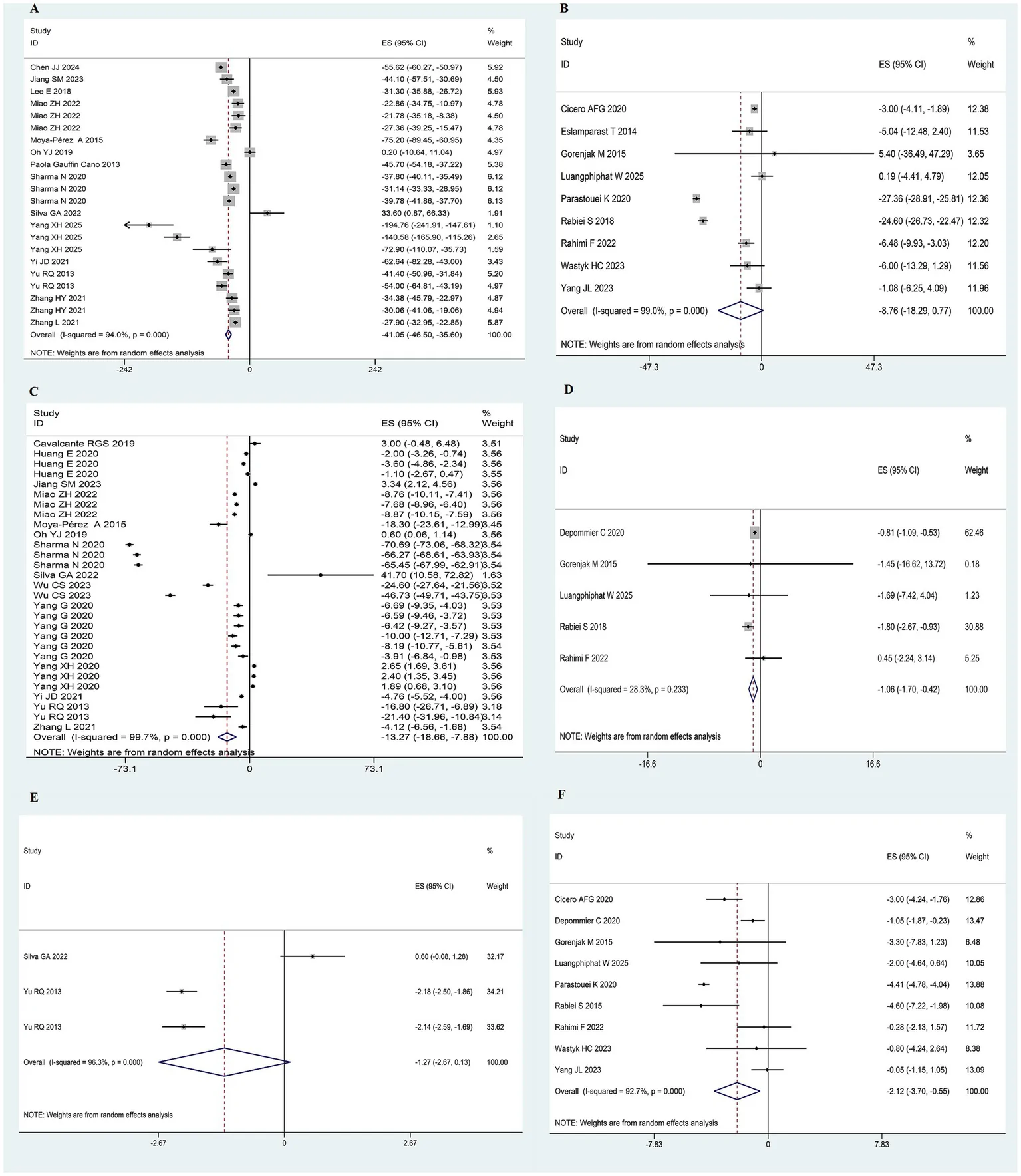
Forest plot of meta-analysis for animal and human studies on fasting plasma glucose (FPG), body weight (BW), and waist circumference (WC). Animal data: FPG **(A)**, BW **(C)**, WC **(E)**; Human data: FPG **(B)**, BW **(D)**, WC **(F)**.

**Table 2:** Summary table of the results for each outcomes.

**(A)** Experimental outcomes (Animal studies)								
**Outcomes**	**Subgroup**		**Included study**	**Experimental group (*N*)**	**Control group (*N*)**	***I*** ^***2***^ ***%***	**MD (95% CI)**	***P***
FPG	Overall		14	185	123	94.00	-41.05 (−46.50, −35.60)	<0.001
	Intervention duration	<12 weeks	4	64	32	94.50	−64.86 (−82.95, −46.77)	<0.001
		≥12 weeks	10	121	91	94.20	−35.36 (−41.17, −29.54)	<0.001
	Probiotic dosage(dichotomous)	<10^9^ CFU	6	60	41	96.90	−54.05 (−72.26, −35.85)	<0.001
		≥10^9^ CFU	10	125	82	88.90	−38.93 (−43.70, −34.16)	<0.001
	Probiotic dosage(stratified)	10^7^–10^8^ CFU	3	24	15	96.70	−78.89 (−120.16, −37.62)	<0.001
		10^8^–10^9^ CFU	5	36	27	99.60	−44.71 (−68.22, −21.21)	<0.001
		10^9^–10^10^ CFU	8	103	60	88.10	−39.03 (−43.53, −34.53)	<0.001
		10^10^–10^11^ CFU	1	12	13	-	33.60 (0.87, 66.33)	0.04
		≥10^11^ CFU	1	10	10	-	−62.64 (−82.28, −43.00)	<0.001
HbA1c	Overall		2	26	18	24.30	−1.17 (−1.50, −0.84)	<0.001
	Intervention duration	<12 weeks	1	16	8	0.00	−0.99 (−1.33, −0.66)	<0.001
		≥12 weeks	1	10	10	-	−1.45 (−1.89, −1.01)	<0.001
	Probiotic dosage (dichotomous)	<10^9^ CFU	1	10	10	-	−1.45 (−1.89, −1.01)	<0.001
		≥10^9^ CFU	1	16	8	0.00	−0.99 (−1.33, −0.66)	<0.001
	Probiotic dosage(stratified)	10^7^–10^8^ CFU	1	10	10	-	−1.45 (−1.89, −1.01)	<0.001
		10^9^–10^10^ CFU	1	16	8	0.00	−0.99 (−1.33, −0.66)	<0.001
BW	Overall		14	249	125	99.70	−13.27 (−18.66, −7.88)	< 0.001
	Intervention duration	<12 weeks	3	50	26	85.60	0.89 (−1.20, 2.97)	0.41
		≥12 weeks	11	199	99	99.80	−15.50 (−22.17, −8.84)	<0.001
	Probiotic dosage(dichotomous)	<10^9^ CFU	4	46	23	99.20	−5.70 (−10.39, −1.01)	0.02
		≥10^9^ CFU	13	203	102	99.70	−14.98 (−22.80, −7.16)	<0.001
	Probiotic dosage(stratified)	10^7^–10^8^ CFU	2	14	5	99.40	−3.04 (−14.23, 8.14)	0.59
		10^8^–10^9^ CFU	4	32	19	99.30	−7.10 (−13.53, −0.68)	0.03
		10^9^–10^10^ CFU	11	181	80	99.80	−17.07 (−26.20, −7.94)	<0.001
		10^10^–10^11^ CFU	1	12	12	–	18.86(−19.60, 57.32)	0.01
		≥10^11^ CFU	1	10	10	–	−4.76 (−5.52, −4.00)	<0.001
WC	Overall		2	36	24	96.30	−1.27 (−2.67, 0.13)	0.08
	Intervention duration	<12 weeks	1	24	12	0.00	−2.17 (−2.43, −1.90)	<0.001
		≥12 weeks	1	12	12	–	0.60 (−0.08, 1.28)	0.09
	Probiotic dosage(dichotomous)	≥10^9^ CFU	2	36	24	96.30	−1.27 (−2.67, 0.13)	0.08
	Probiotic dosage(stratified)	10^9^–10^10^ CFU	1	24	12	0.00	−2.17 (−2.43, −1.90)	<0.001
		10^10^–10^11^ CFU	1	12	12	-	0.60 (−0.08, 1.28)	0.09
TC	Overall		17	352	162	99.40	−36.66 (−50.10, −23.22)	<0.001
	Intervention duration	<12 weeks	5	124	46	98.90	−39.41 (−59.74, −19.07)	<0.001
		≥12 weeks	12	228	116	99.50	−35.18 (−52.19, −18.18)	<0.001
	Probiotic dosage (dichotomous)	<10^9^ CFU	5	89	45	99.40	−52.98 (−74.34, −31.62)	<0.001
		≥10^9^ CFU	13	263	117	99.40	−30.55 (−48.30, −12.80)	0.001
	probiotic dosage(stratified)	10^7^–10^8^ CFU	2	26	18	99.30	−68.73 (−125.20, −12.26)	0.02
		10^8^–10^9^ CFU	3	63	27	99.50	−46.21 (−69.93, −22.50)	<0.001
		10^9^–10^10^ CFU	11	241	95	99.40	−30.11 (−48.96, −11.25)	0.002
		10^10^–10^11^ CFU	1	12	12	–	−8.60 (−12.08, −5.12)	<0.001
		≥10^11^ CFU	1	10	10	–	−64.19 (−82.84, −45.55)	<0.001
TG	Overall		17	348	158	99.90	−51.38 (−71.46, −31.30)	<0.001
	Intervention duration	<12 weeks	6	130	52	99.10	−49.63 (−66.44, −32.81)	<0.001
		≥12 weeks	11	218	106	99.90	−52.39 (−79.05, −25.73)	<0.001
	Probiotic dosage(dichotomous)	<10^9^ CFU	5	85	41	99.70	−52.24 (−80.37, −24.11)	<0.001
		≥10^9^ CFU	13	263	117	99.90	−51.29 (−82.32, −20.27)	0.001
	Probiotic dosage(stratified)	10^7^–10^8^ CFU	2	26	18	99.10	−95.59 (−162.76, −28.42)	0.005
		10^8^–10^9^ CFU	3	59	23	99.80	−33.85 (−67.22, −0.48)	0.047
		10^9^–10^10^ CFU	11	241	95	99.90	−52.70 (−85.14, −20.26)	0.001
		10^10^–10^11^ CFU	1	12	12	–	−33.20 (−41.71, −24.69)	<0.001
		≥10^11^ CFU	1	10	10	–	−31.86 (−50.09, −13.63)	0.001
LDL-C	Overall		14	287	133	99.60	−18.86 (−26.48, −11.24)	<0.001
	Intervention duration	<12 weeks	6	130	52	99.30	−21.78 (−29.34, −14.22)	<0.001
		≥12 weeks	8	157	81	99.60	−16.21 (−29.29, −3.13)	0.02
	Probiotic dosage(dichotomous)	<10^9^ CFU	5	50	42	99.90	−46.99 (−69.75, −24.23)	<0.001
		≥10^9^ CFU	10	237	91	99.40	−12.34 (−20.33, −4.34)	0.002
	Probiotic dosage(stratified)	10^7^–10^8^ CFU	2	26	18	99.80	−58.91 (−121.83, 4.00)	0.07
		10^8^–10^9^ CFU	3	24	24	99.90	−35.30 (−72.20, 1.60)	0.06
		10^9^–10^10^ CFU	9	225	79	99.40	−12.43 (−20.75, −4.10)	0.003
		10^10^–10^11^ CFU	1	12	12	–	10.00 (−13.40, −6.60)	<0.001
HDL-C	Overall		13	269	127	98.60	10.03 (7.13, 12.93)	<0.001
	Intervention duration	<12 weeks	6	130	52	98.10	12.23 (8.29, 16.17)	<0.001
		≥12 weeks	7	139	75	98.80	6.65 (1.96, 11.34)	0.01
	Probiotic dosage(dichotomous)	<10^9^ CFU	5	50	42	98.90	5.98 (1.01, 10.95)	0.02
		≥10^9^ CFU	9	219	85	98.50	12.23 (8.23, 16.23)	<0.001
	Probiotic dosage(stratified)	10^7^–10^8^ CFU	2	26	18	98.10	9.90 (3.96, 15.84)	0.001
		10^8^–10^9^ CFU	3	24	24	99.40	1.97 (−8.61, 12.55)	0.72
		10^9^–10^10^ CFU	8	207	73	97.80	13.14 (8.91, 17.38)	<0.001
		10^10^–10^11^ CFU	1	12	12	-	1.10 (0.50, 1.70)	<0.001
Insulin	Overall		11	135	101	99.70	−3.33 (−3.64, −3.03)	<0.001
	Intervention duration	<12 weeks	3	46	26	96.60	−7.05 (−8.85, −5.25)	<0.001
		≥12 weeks	8	89	75	99.70	−1.95 (−2.24, −1.66)	<0.001
	Probiotic dosage (dichotomous)	<10^9^ CFU	3	26	26	98.30	−6.34 (−10.98, −1.70)	0.007
		≥10^9^ CFU	8	109	75	99.60	−2.29 (−2.54, −2.04)	<0.001
	probiotic dosage (stratified)	10^7^–10^8^ CFU	1	10	10	–	−8.19 (−8.53, −7.85)	< 0.001
		10^8^–10^9^ CFU	2	16	16	98.40	−5.41 (−13.79, 2.97)	0.21
		10^9^–10^10^ CFU	6	87	53	99.60	−2.59 (−2.87, −2.32)	<0.001
		10^10^–10^11^ CFU	1	12	12	–	0.00 (−0.36, 0.36)	1.00
		≥10^11^ CFU	1	10	10	–	−2.09 (−2.71, −1.47)	<0.001
HOMA-IR	Overall		6	68	60	99.50	−3.02 (−5.69, −0.36)	0.03
	Intervention duration	<12 weeks	1	16	8	0.00	−4.53 (−6.01, −3.06)	<0.001
		≥12 weeks	5	52	52	99.70	−2.57 (−5.70, 0.56)	0.11
	Probiotic dosage(dichotomous)	<10^9^ CFU	2	20	20	99.90	−4.59 (−12.31, 3.13)	0.24
		≥10^9^ CFU	4	48	40	93.80	−2.05 (−3.25, −0.84)	0.001
	probiotic dosage(stratified)	10^7^–10^8^ CFU	1	10	10	-	−8.53 (−8.93, −8.13)	<0.001
		10^8^–10^9^ CFU	1	10	10	-	−0.65 (−1.00, −0.30)	<0.001
		10^9^–10^10^ CFU	2	26	18	89.40	−3.09 (−5.85, −0.34)	0.03
		10^10^–10^11^ CFU	1	12	12	–	0.00 (−0.38, 0.38)	1.00
		≥10^11^ CFU	1	10	10	–	−2.48 (−3.24, −1.72)	<0.001
HOMA-β			1	16	8	–	–	–
OGTT 0 h Blood Glucose	Overall		2	40	16	0.00	−1.34 (−1.65, −1.03)	<0.001
	Intervention duration	<12 weeks	1	16	8	0.00	−1.56 (−2.05, −1.07)	<0.001
		≥12 weeks	1	24	8	18.70	−1.17 (−1.64, −0.70)	<0.001
	Probiotic dosage(dichotomous)	<10^9^ CFU	1	16	5	0.00	−1.39 (−1.88, −0.89)	<0.001
		≥10^9^ CFU	2	24	11	30.60	−1.31 (−1.80, −0.83)	<0.001
	Probiotic dosage(stratified)	10^7^–10^8^ CFU	1	8	3	–	−0.85 (−2.13, 0.43)	0.20
		10^8^–10^9^ CFU	1	8	3	–	−1.48 (−2.02, −0.94)	<0.001
		10^9^–10^10^ CFU	2	24	11	30.60	−1.31 (−1.80, −0.83)	<0.001
OGTT 2 h Blood Glucose	Overall		2	40	16	82.10	−2.33 (−3.45, −1.21)	<0.001
	Intervention duration	<12 weeks	1	16	8	36.10	−2.18 (−2.96, −1.41)	<0.001
		≥12 weeks	1	24	8	87.60	−2.25 (−4.35, −0.14)	0.04
	Probiotic dosage(dichotomous)	<10^9^ CFU	1	16	5	92.30	−2.01 (−6.33, 2.30)	0.36
		≥10^9^ CFU	2	24	11	0.00	−2.19 (−2.73, −1.64)	0.00
	Probiotic dosage(stratified)	10^7^–10^8^ CFU	1	8	3	–	0.32 (−1.93, 2.57)	0.78
		10^8^–10^9^ CFU	1	8	3	–	−4.09 (−4.93, −3.25)	<0.001
		10^9^–10^10^ CFU	2	24	11	0.00	−2.19 (−2.73, −1.64)	<0.001
CRP	Overall		2	70	20	98.80	−33.63(−52.45,-14.81)	<0.001
	Intervention duration	≥12 weeks	2	70	20	98.80	−33.63(−52.45,-14.81)	<0.001
	Probiotic dosage(dichotomous)	≥10^9^ CFU	2	70	20	98.80	−33.63(−52.45,-14.81)	<0.001
	Probiotic dosage(stratified)	10^9^–10^10^ CFU	2	70	20	98.80	−33.63(−52.45,-14.81)	<0.001
IL-6	Overall		4	104	54	64.20	−15.07 (−18.23, −11.91)	<0.001
	Intervention duration	<12 weeks	1	24	24	77.50	−15.87 (−22.89, −8.84)	<0.001
		≥12 weeks	3	80	30	53.80	−14.55 (−18.00, −11.10)	<0.001
	Probiotic dosage (dichotomous)	<10^9^ CFU	1	16	16	81.80	−11.76 (−24.77, 1.26)	0.08
		≥10^9^ CFU	4	88	38	64.30	−15.48 (−18.99, −11.98)	<0.001
	Probiotic dosage(stratified)	10^7^–10^8^ CFU	1	8	8	–	−4.25 (−14.47, 5.97)	0.42
		10^8^–10^9^ CFU	1	8	8	–	−17.63 (−22.17, −13.09)	<0.001
		10^9^–10^10^ CFU	3	80	30	53.80	−14.55 (−18.00, −11.10)	<0.001
		10^10^–10^11^ CFU	1	8	8	-	−21.09 (−25.47, −16.71)	<0.001
IL-1β	Overall		5	114	64	96.70	−5.11(−7.27,-2.95)	<0.001
	Intervention duration	<12 weeks	1	24	24	0.00	−1.82 (−2.39, −1.24)	<0.001
		≥12 weeks	4	90	40	97.40	6.50 (−11.16, −1.85)	0.006
	Probiotic dosage (dichotomous)	<10^9^ CFU	2	26	26	89.20	−0.94 (−2.29, 0.41)	0.17
		≥10^9^ CFU	4	88	38	94.40	−6.86 (−10.50, −3.21)	<0.001
	Probiotic dosage (stratified)	10^7^–10^8^ CFU	1	8	8	–	−1.29 (−2.25, −0.33)	0.008
		10^8^–10^9^ CFU	2	18	18	91.20	−0.82 (−2.79, 1.16)	0.42
		10^9^–10^10^ CFU	3	80	30	90.30	−7.60 (−11.31, −3.89)	<0.001
		10^10^–10^11^ CFU	1	8	8	–	−2.24 (−3.16, −1.32)	<0.001
TNF-α	Overall		5	114	64	94.80	−31.42 (−44.86, −17.98)	<0.001
	Intervention duration	<12 weeks	1	24	24	0.00	−13.52 (−17.87, −9.16)	<0.001
		≥12 weeks	4	90	40	95.40	−41.36 (−71.75, −10.97)	0.01
	Probiotic dosage(dichotomous)	<10^9^ CFU	2	26	26	91.10	−7.68 (−17.54, 2.17)	0.13
		≥10^9^ CFU	4	88	38	88.80	−43.54 (−67.00, −20.08)	<0.001
	Probiotic dosage(stratified)	10^7^–10^8^ CFU	1	8	8	-	−9.41 (−17.02, −1.80)	0.02
		10^8^–10^9^ CFU	2	18	18	94.10	−7.07 (−20.94, 6.79)	0.32
		10^9^–10^10^ CFU	4	80	30	79.30	−47.86 (−71.38, −24.34)	<0.001
		10^10^–10^11^ CFU	1	8	8	-	−17.09 (−25.76, −8.42)	<0.001
LPS	Overall		2	70	20	86.00	−55.23 (−83.55, −26.91)	<0.001
	Intervention duration	≥12 weeks	2	70	20	86.00	−55.23 (−83.55, −26.91)	<0.001
	Probiotic dosage(dichotomous)	≥10^9^ CFU	2	70	20	86.00	−55.23 (−83.55, −26.91)	<0.001
	Probiotic dosage(stratified)	10^9^–10^10^ CFU	2	70	20	86.00	−55.23 (−83.55, −26.91)	<0.001
FFA	Overall		4	92	42	99.70	−130.36 (−191.52, −69.21)	<0.001
	Intervention duration	≥12 weeks	4	92	42	99.70	−130.36 (−191.52, −69.21)	<0.001
	Probiotic dosage(dichotomous)	<10^9^ CFU	1	10	10	–	−680.00 (−829.01, −530.99)	<0.001
		≥10^9^ CFU	3	82	32	99.70	−89.59 (−151.93, −27.25)	0.01
	Probiotic dosage(stratified)	10^7^–10^8^ CFU	1	10	10	–	−680.00 (−829.01, −530.99)	<0.001
		10^9^–10^10^ CFU	2	70	20	99.10	−102.32 (−157.74, −46.89)	<0.001
		10^10^–10^11^ CFU	1	12	12	-	−1.16 (−1.32, −1.01)	<0.001
Adiponectin			1	18	6	61.40	1.07 (0.46, 1.68)	0.001
Leptin	Overall		3	34	22	94.50	−20.95 (−39.79, −2.11)	0.03
	Intervention duration	≥12 weeks	3	34	22	94.50	−20.95 (−39.79, −2.11)	0.03
	Probiotic dosage(dichotomous)	≥10^9^ CFU	3	34	22	94.50	−20.95 (−39.79, −2.11)	0.03
	Probiotic dosage(stratified)	10^9^–10^10^ CFU	3	34	22	94.50	−20.95 (−39.79, −2.11)	0.03
ALT	Overall		4	92	42	99.50	−9.72 (−14.69, −4.75)	< 0.001
	Intervention duration	<12 weeks	1	16	16	74.30	−2.50 (−6.81, 1.81)	0.26
		≥12 weeks	3	76	26	99.60	−11.29 (−16.91, −5.66)	<0.001
	Probiotic dosage(dichotomous)	<10^9^ CFU	2	61	25	90.40	−7.83 (−9.71, −5.95)	<0.001
		≥10^9^ CFU	2	31	17	99.70	−13.67 (−28.24, 0.90)	0.07
	Probiotic dosage(stratified)	10^7^–10^8^ CFU	1	16	16	74.30	−2.50 (−6.81, 1.81)	0.26
		10^8^–10^9^ CFU	1	45	9	83.00	−9.40 (−10.80, −8.01)	<0.001
		10^9^–10^10^ CFU	2	31	17	99.70	−13.67 (−28.24, 0.90)	0.07
AST	Overall		3	82	32	98.80	−25.17 (−33.49, −16.84)	<0.001
	Intervention duration	<12 weeks	1	16	16	88.80	2.40 (−7.30, 2.50)	0.34
		≥12 weeks	2	66	16	94.20	−30.64 (−35.18, −26.11)	<0.001
	Probiotic dosage(dichotomous)	<10^9^ CFU	2	61	25	99.00	−19.28 (−28.43, −10.13)	<0.001
		≥10^9^ CFU	1	21	7	88.60	−39.33 (−48.19, −30.47)	<0.001
	Probiotic dosage(stratified)	10^7^–10^8^ CFU	1	16	16	88.80	−2.40 (−7.30, 2.50)	0.34
		10^8^–10^9^ CFU	1	45	9	89.20	−26.03 (−29.46, −22.60)	< 0.001
		10^9^–10^10^ CFU	1	21	7	88.60	−39.33 (−48.19, −30.47)	< 0.001
-: not applicable; BW: body weight; BMI: body mass index; WC: waist circumference; FPG: Fasting Plasma glucose; HbA1c: Hemoglobin A1c; TC: Total Cholesterol; LDL-C: Low-density lipoprotein cholesterol; TG: Triglycerides; HDL-C: High-density lipoprotein cholesterol; HOMA-IR: Homeostasis model assessment of insulin resistance; HOMA-β: Homeostasis model assessment of β-cell function; OGTT: Oral Glucose Tolerance Test; CRP: C-reactive protein; IL-6: Interleukin-6; IL-1β: Interleukin-1 beta; TNF-α: Tumor Necrosis Factor-alpha; LPS: Lipopolysaccharides; FFA: Free fatty acids; ALT: Alanine aminotransferase activity; AST: Aspartate aminotransferase activity.								

**(B)** Clinical outcomes (Human studies)								
**Outcomes**	**Subgroup**		**Included study**	**Experimental Group(N)**	**Control Group (N)**	***I*** ^***2***^ ***%***	**MD (95% CI)**	***P***
FPG	Overall		9	280	270	99.00	−8.76 (−18.29, 0.77)	0.07
	Intervention duration	<12 weeks	3	81	73	99.70	−12.21 (−31.37, 6.95)	0.21
		≥12 weeks	6	199	197	97.20	−6.82 (−18.24, 4.60)	0.24
	Mean age	<50 years	7	236	234	99.10	−6.56 (−18.03, 4.90)	0.26
		≥50 years	2	44	36	95.70	−15.64 (−33.86, 2.57)	0.09
	Baseline mean BMI	no data	1	21	13	-	−6.00 (−13.29, 1.29)	0.11
		25–29.9 kg/m^2^	3	127	121	7.60	−2.59 (−3.98, −1.21)	< 0.001
		≥30 kg/m^2^	5	132	136	97.20	−15.46 (−24.53, −6.39)	0.001
	Probiotic dosage(dichotomous)	<10^9^ CFU	2	42	42	95.90	−15.16 (−34.32, 4.00)	0.12
		≥10^9^ CFU	7	238	228	99.10	−6.71 (−18.16, 4.74)	0.25
HbA1c	Overall		3	106	102	0.00	−0.08 (−0.15, −0.01)	0.03
	Intervention duration	≥12 weeks	3	106	102	0.00	−0.08 (−0.15, −0.01)	0.03
	Mean age	<50 years	2	97	91	0.00	−0.07 (−0.15, 0.01)	0.07
		≥50 years	1	9	11	-	−0.09 (−0.24, 0.06)	0.24
	Baseline mean BMI	25–29.9 kg/m^2^	2	97	91	0.00	−0.07 (−0.15, 0.01)	0.07
		≥30 kg/m^2^	1	9	11	-	−0.09 (−0.24, 0.06)	0.24
	Probiotic dosage(dichotomous)	≥10^9^ CFU	3	106	102	0.00	−0.08 (−0.15, −0.01)	0.03
BW	Overall		5	123	125	28.30	−1.06 (−1.70, −0.42)	0.001
	Intervention duration	≥12 weeks	5	123	125	28.30	−1.06 (−1.70, −0.42)	0.001
	Mean age	<50 years	3	91	91	0.00	0.03 (−2.38, 2.43)	0.98
		≥50 years	2	32	34	77.90	−1.22 (−2.17, −0.26)	0.01
	Baseline mean BMI	25–29.9 kg/m^2^	1	31	27	-	−1.69 (−7.42, 4.04)	0.56
		≥30 kg/m^2^	4	92	98	45.50	−1.07 (−1.83, −0.30)	0.01
	Probiotic dosage(dichotomous)	<10^9^ CFU	1	23	23	-	−1.80 (−2.67, −0.93)	<0.001
		≥10^9^ CFU	4	100	102	0.00	−0.80 (−1.08, −0.52)	<0.001
WC	Overall		9	270	262	92.70	−2.12 (−3.70, −0.55)	0.01
	Intervention duration	<12 weeks	3	81	73	76.30	−3.36 (−4.91, −1.82)	<0.001
		≥12 weeks	6	189	189	59.10	−1.31 (−2.44, −0.19)	0.02
	Mean age	<50 years	6	217	215	93.00	−2.13 (−4.14, −0.13)	0.04
		≥50 years	3	53	47	69.40	−2.06 (−4.38, 0.26)	0.08
	Baseline mean BMI	no data	1	21	13	-	−0.80 (−4.24, 2.64)	0.65
		25–29.9 kg/m^2^	3	127	121	83.70	−1.63 (−3.79, 0.53)	0.14
		≥30 kg/m^2^	5	122	128	94.00	−2.64 (−4.83, −0.44)	0.02
	Probiotic dosage(dichotomous)	<10^9^ CFU	1	23	23	-	−4.60 (−7.22, −1.98)	0.001
		≥10^9^ CFU	8	247	239	93.60	−1.85 (−3.53, −0.16)	0.03
TC	Overall		9	242	244	90.00	−12.09 (−16.15, −8.04)	< 0.001
	Intervention duration	<12 weeks	2	60	60	0.00	−11.67 (−13.30, −10.04)	< 0.001
		≥12 weeks	7	182	184	92.40	−12.73 (−19.00, −6.47)	< 0.001
	Mean age	<50 years	7	210	210	88.60	−13.60 (−19.40, −7.80)	< 0.001
		≥50 years	2	32	34	94.50	−8.74 (−15.61, −1.87)	0.01
	Baseline mean BMI	no data	1	40	40	-	−16.00 (−19.21, −12.79)	< 0.001
		25–29.9 kg/m^2^	2	61	57	97.20	−26.56 (−60.07, 6.95)	0.12
		≥30 kg/m^2^	6	141	147	83.50	−8.38 (−12.14, −4.62)	< 0.001
	Probiotic dosage(dichotomous)	<10^9^ CFU	2	42	42	0.00	−5.37 (−7.77, −2.96)	< 0.001
		≥10^9^ CFU	7	200	202	88.50	−13.71 (−18.06, −9.35)	< 0.001
TG	Overall		10	263	257	89.80	−20.51 (−27.69, −13.34)	< 0.001
	Intervention duration	<12 weeks	3	81	73	91.20	−18.10 (−31.85, −4.36)	0.01
		≥12 weeks	7	182	184	86.50	−21.40 (−30.13, −12.67)	< 0.001
	Mean age	<50 years	7	210	210	83.80	−22.45 (−30.67, −14.23)	< 0.001
		≥50 years	3	53	47	94.00	−15.70 (−32.49, 1.08)	0.07
	Baseline mean BMI	no data	2	61	53	84.40	−8.45 (−34.64, 17.73)	0.53
		25–29.9 kg/m^2^	2	61	57	0.00	−18.32 (−24.06, −12.59)	<0.001
		≥30 kg/m^2^	6	141	147	93.60	−25.08 (−36.78, −13.37)	<0.001
	Probiotic dosage (dichotomous)	<10^9^ CFU	2	42	42	91.80	−35.09 (−80.49, 10.32)	0.13
		≥10^9^ CFU	8	221	215	84.50	19.83 (−27.15, −12.51)	<0.001
LDL-C	Overall		9	242	244	94.10	−10.56 (−14.96, −6.17)	<0.001
	Intervention duration	<12 weeks	2	60	60	0.00	−9.16 (−10.48, −7.83)	<0.001
		≥12 weeks	7	182	184	95.60	−11.00 (−17.96, −4.04)	0.002
	Mean age	<50 years	7	210	210	95.10	−11.72 (−18.08, −5.35)	<0.001
		≥50 years	2	32	34	52.30	−6.93 (−9.38, −4.47)	<0.001
	Baseline mean BMI	no data	1	40	40	–	−13.00 (−15.43, −10.57)	<0.001
		25–29.9 kg/m^2^	2	61	57	98.70	−25.18 (−57.95, 7.59)	0.13
		≥30 kg/m^2^	6	141	147	80.60	−5.84 (−8.95, −2.73)	<0.001
	Probiotic dosage(dichotomous)	<10^9^ CFU	2	42	42	0.00	−8.15 (−10.89, −5.41)	<0.001
		≥10^9^ CFU	7	200	202	95.60	−11.57 (−16.92, −6.21)	<0.001
HDL-C	Overall		9	254	246	92.70	1.97 (0.55, 3.39)	0.01
	Intervention duration	<12 weeks	3	81	73	94.00	1.52 (−1.05, 4.09)	0.25
		≥12 weeks	6	173	173	93.40	2.29 (−0.21, 4.79)	0.07
	Mean age	<50 years	7	210	210	93.80	2.51 (0.45, 4.56)	0.02
		≥50 years	3	44	36	0.00	0.30 (−0.16, 0.75)	0.20
	Baseline mean BMI	no data	2	61	53	0.00	1.87 (0.74, 2.99)	0.001
		25–29.9 kg/m^2^	2	61	57	0.00	3.40 (2.51, 4.30)	< 0.001
		≥30 kg/m^2^	5	132	136	94.40	1.71 (−0.10, 3.51)	0.06
	Probiotic dosage(dichotomous)	<10^9^ CFU	2	42	42	98.50	3.97 (−3.31, 11.25)	0.29
		≥10^9^ CFU	7	212	204	85.50	1.29 (−0.22, 2.80)	0.09
Insulin	Overall		4	114	120	99.40	−9.67 (−14.66, −4.69)	<0.001
	Intervention duration	<12 weeks	1	30	30	-	−0.50 (−1.24, 0.24)	0.19
		≥12 weeks	3	84	90	99.50	−14.45 (−21.26, −7.64)	<0.001
	Mean age	<50 years	2	82	86	32.80	−0.17 (−0.76, 0.43)	0.59
		≥50 years	2	32	34	99.00	−28.79 (−72.55, 14.96)	0.20
	Baseline mean BMI	25–29.9 kg/m^2^	1	84	30	-	−0.50 (−1.24, 0.24)	0.19
		≥30 kg/m^2^	3	30	90	99.50	−14.45 (−21.26, −7.64)	<0.001
	Probiotic dosage (dichotomous)	<10^9^ CFU	1	23	23	–	−6.70 (−7.15, −6.25)	<0.001
		≥10^9^ CFU	3	91	97	98.40	−9.31 (−14.30, −4.33)	<0.001
HOMA-IR	Overall		4	171	173	99.60	−0.86 (−2.85,1.13)	0.40
	Intervention duration	<12 weeks	1	30	30	–	0.50 (0.19, 0.81)	0.001
		≥12 weeks	3	141	143	99.70	−1.31 (−3.91, 1.29)	0.32
	Mean age	<50 years	3	148	150	82.50	0.07 (−0.36, 0.51)	0.74
		≥50 years	1	23	23	-	−3.50 (−3.73, −3.27)	0.00
	Baseline mean BMI	25–29.9 kg/m^2^	2	96	94	85.10	0.09 (−0.77, 0.96)	0.83
		≥30 kg/m^2^	2	75	79	99.80	−1.76 (−5.16, 1.64)	0.31
	Probiotic dosage(dichotomous)	<10^9^ CFU	1	23	23	-	−3.50 (−3.73, −3.27)	<0.001
		≥10^9^ CFU	3	148	150	82.50	0.07 (−0.36, 0.51)	0.74
HOMA-β			2	118	120	72.70	2.90(−19.03,24.83)	0.80
OGTT 2 h Blood Glucose			1	66	64	–	–	–
CRP			2	49	51	0.00	−1.20(−1.33,-1.07)	<0.001
IL-6			1	40	40	–	–	–
TNF-α			2	70	70	97.60	−1.87 (−4.71, 0.97)	0.20
LPS			1	9	11	–	–	–
Adiponectin			2	38	38	93.30	2.39(−3.40,8.18)	0.42
Leptin			1	30	30	–	–	–
ALT	Overall		5	188	188	86.60	−1.13 (−3.14, 0.87)	0.27
	Intervention duration	<12 weeks	1	30	30	-	−1.80 (−2.72, −0.88)	<0.001
		≥12 weeks	4	158	158	77.40	−0.92 (−3.80, 1.96)	0.53
	Mean age	<50 years	4	179	177	36.40	−2.00 (−3.63, −0.38)	0.02
		≥50 years	1	9	11	-	0.75 (0.13, 1.37)	0.02
	Baseline mean BMI	25–29.9 kg/m^2^	3	127	121	54.30	−1.61 (−5.02, 1.80)	0.36
		≥30 kg/m^2^	2	61	67	86.80	−0.70 (−3.90, 2.49)	0.67
	Probiotic dosage(dichotomous)	≥10^9^ CFU	5	188	188	86.60	−1.13 (−3.14, 0.87)	0.27
AST	Overall		5	188	188	58.40	−0.95 (−2.00, 0.11)	0.08
	Intervention duration	<12 weeks	1	30	30	-	−1.50 (−2.57, −0.43)	0.006
		≥12 weeks	4	158	158	58.40	−0.73 (−2.24, 0.78)	0.34
	Mean age	<50 years	4	179	177	54.90	−1.16 (−2.73, 0.41)	0.15
		≥50 years	1	9	11	-	−0.45 (−1.05, 0.15)	0.14
	Baseline mean BMI	25–29.9 kg/m^2^	3	127	121	69.90	−0.86 (−3.44, 1.71)	0.51
		≥30 kg/m^2^	2	61	67	0.00	−0.54 (−1.11, 0.03)	0.06
	Probiotic dosage(dichotomous)	≥10^9^ CFU	5	188	188	58.40	−0.95 (−2.00, 0.11)	0.08

–: not applicable; BW: body weight; BMI: body mass index; WC: waist circumference; FPG: Fasting Plasma glucose; HbA1c: Hemoglobin A1c; TC: Total Cholesterol; LDL-C: Low-density lipoprotein cholesterol; TG: Triglycerides; HDL-C: High-density lipoprotein cholesterol; HOMA-IR: Homeostasis model assessment of insulin resistance; HOMA-β: Homeostasis model assessment of β-cell function; OGTT: Oral Glucose Tolerance Test; CRP: C-reactive protein; IL-6: Interleukin-6; IL-1β: Interleukin-1 beta; TNF-α: Tumor Necrosis Factor-alpha; LPS: Lipopolysaccharides; FFA: Free fatty acids; ALT: Alanine aminotransferase activity; AST: Aspartate aminotransferase activity.

#### 3.5.2 Change in HbA1c

Meta-analysis of 2 animal studies involving 44 animals with MetS and 4 clinical studies involving 208 patients with MetS showed that probiotic intervention significantly reduced HbA1c compared with placebo, with (MD = −1.17%, 95% CI: −1.50 to −0.84, *p* < 0.001) in animals (Table 2A and Supplementary material 5.1.1) and (MD = −0.08%, 95% CI: −0.15 to −0.01, *p* = 0.03) in patients (Table 2B and Supplementary material 5.2.1).

### 3.6 Effect on anthropometric measurements

#### 3.6.1 Change in BW

Meta-analysis of 14 animal studies involving 374 mice with MetS and 5 clinical studies involving 248 MetS patients showed that probiotic intervention significantly reduced BW compared with placebo, with (MD = −13.27 g, 95% CI: −18.66 to −7.88, *p* < 0.001) in animals (Figure 2C and Table 2A) and (MD = −1.06 kg, 95% CI: −1.70 to −0.42, *p* = 0.001) in patients (Figure 2D and Table 2B).

#### 3.6.2 Change in WC

Meta-analysis of 2 animal studies involving 60 animals with MetS and 9 clinical studies involving 532 patients with MetS showed that probiotic intervention did not reduce WC in animals compared with placebo (MD = −1.27 cm, 95% CI: −2.67 to 0.13, *p* = 0.08) (Figure 2E and Table 2A), but significantly decreased WC in patients (MD = −2.12 cm, 95% CI: −3.70 to −0.55, *p* = 0.01) (Figure 2F and Table 2B).

### 3.7 Effect on lipid metabolism

#### 3.7.1 Animal studies

Meta-analyses including a total of 17 studies (506 animals), 13 studies (396 animals), 17 studies (514 animals), and 14 studies (420 animals) with MetS showed that probiotic intervention significantly improved lipid profiles compared with placebo, with TG decreased by MD = −51.38 mg/dL (95% CI: −71.46 to −31.30, *p* < 0.001) (Figure 3A and Table 2A), HDL-C increased by MD = 10.03 mg/dL (95% CI: 7.13 to 12.93, *p* < 0.001) (Figure 3B and Table 2A), TC reduced by MD = −36.66 mg/dL (95% CI: −50.10 to −23.22, *p* < 0.001) (Figure 3C and Table 2A), and LDL-C reduced by MD = −18.86 mg/dL (95% CI: −26.48 to −11.24, *p* < 0.001) (Figure 3D and Table 2A).

**Figure 3:**
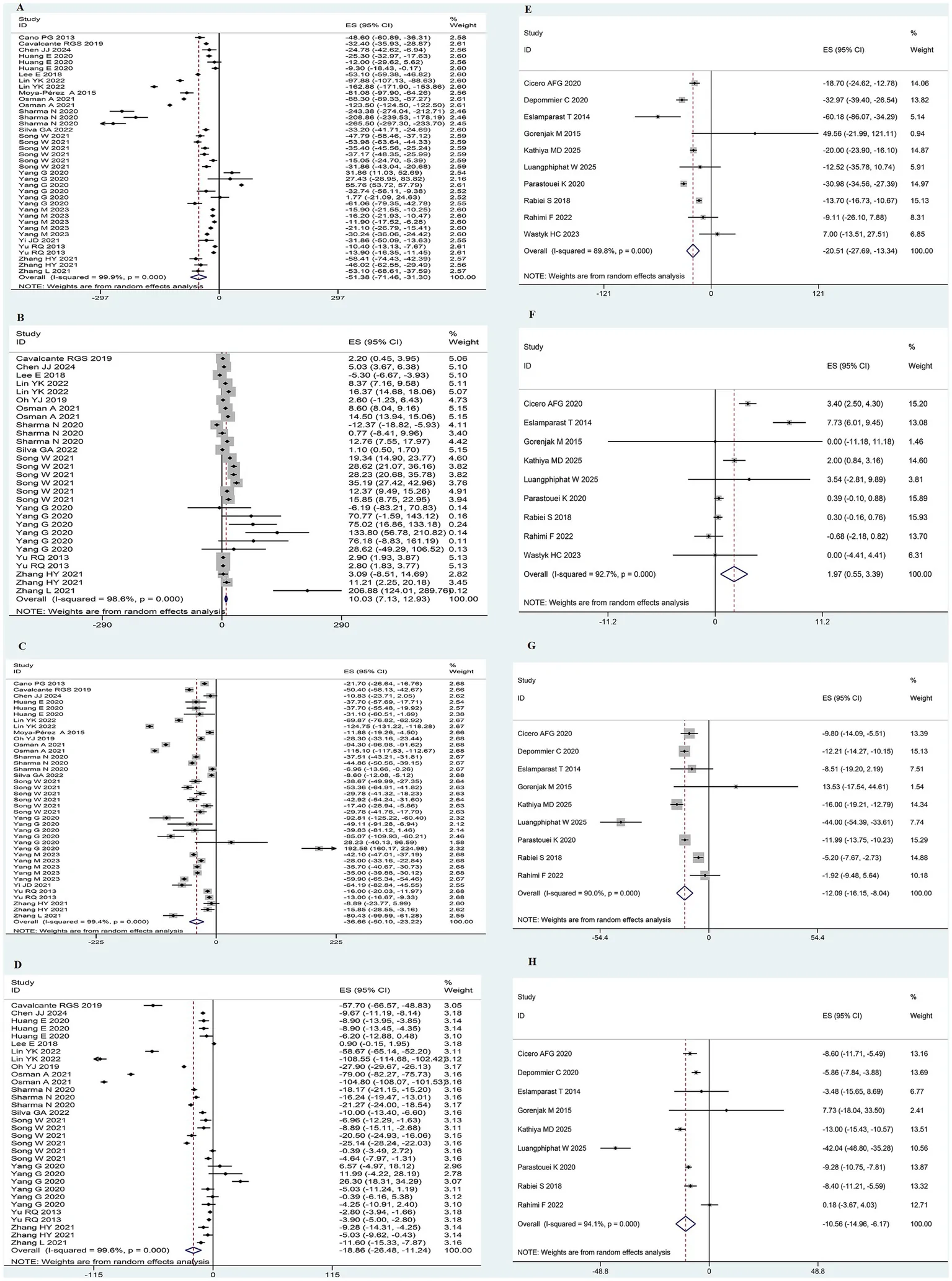
Forest plot of meta-analysis for animal and human studies on triglycerides (TG), high-density lipoprotein cholesterol (HDL-C), total cholesterol (TC), and low-density lipoprotein cholesterol (LDL-C). Animal data: TG **(A)**, HDL-C **(B)**, TC **(C)**, LDL-C **(D)**; Human data: TG **(E)**, HDL-C **(F)**, TC **(G)**, LDL-C **(H)**.

#### 3.7.2 Clinical studies

Meta-analyses including a total of 10 studies (520 patients), 9 studies (500 patients), 9 studies (486 patients), and 9 studies (486 patients) with MetS showed that probiotic intervention significantly improved lipid profiles compared with placebo, with TG decreased by MD = −20.51 mg/dL (95% CI: −27.69 to −13.34, *p* < 0.001) (Figure 3E and Table 2B), HDL-C increased by MD = 1.97 mg/dL (95% CI: 0.55 to 3.39, *p* = 0.01) (Figure 3F and Table 2B), TC reduced by MD = −12.09 mg/dL (95% CI: −16.15 to −8.04, *p* < 0.001) (Figure 3G and Table 2B), and LDL-C reduced by MD = −10.56 mg/dL (95% CI: −14.96 to −6.17, *p* < 0.001) (Figure 3H and Table 2B).

### 3.8 Secondary outcomes

In animal studies, pooled results showed a statistically significant reduction in fasting insulin, HOMA-IR, CRP, ALT, AST, FFA, IL-1β, IL-6, LPS, TNF-α, well as a significant elevation in adiponectin adiponectin levels (Table 2A and Supplementary materials 5.1.2–5.1.15).

In patients with MetS, no significant differences were found in HOMA-IR, HOMA-β, ALT, AST, TNF-α, and adiponectin, but insulin and CRP were significantly reduced (Table 2B and Supplementary materials 5.2.2–5.2.9).

Detailed results of subgroup analyses are presented in Table 2.

### 3.9 Sensitivity analysis

In animal studies, sensitivity analyses showed that FPG, HbA1c, TC, HDL-C, LDL-C, TG, body weight, insulin, CRP, ALT, AST, FFA, IL-1β, IL-6, LPS, TNF-α, adiponectin, leptin, and 0-h and 2-h blood glucose during glucose tolerance testing were stable; WC and HOMA-IR were unstable (Supplementary material 6.1).

In patients with Mets, sensitivity analyses showed that FPG, HbA1c, ALT and AST were unstable, while TC, HDL-C, LDL-C, TG, WC, HOMA-IR, and BW were stable (Supplementary material 6.2).

### 3.10 Publication bias

Egger’s test and enhanced funnel plots were performed for all outcomes. In animal studies, Egger’s test showed that the *p*-values of BW, insulin, IL-1β, TNF-α, FFA, leptin and ALT were all <0.05, suggesting potential publication bias. The funnel plot asymmetry for these outcomes most likely reflects genuine between-study heterogeneity rather than publication bias, because the asymmetric points fell outside the significance contours with no concentration in the non-significant (*p* > 0.10) regions; nevertheless, publication bias cannot be entirely excluded. The remaining indicators had *p*-values >0.05 with good funnel plot symmetry, indicating a low risk of publication bias in animal studies (Figures 4A–C and Supplementary material 7.1). In patients with Mets, all Egger’s test *p*-values were >0.05 and funnel plots were symmetrical, indicating no significant publication bias in this group (Figures 4D–F and Supplementary material 7.2).

**Figure 4:**
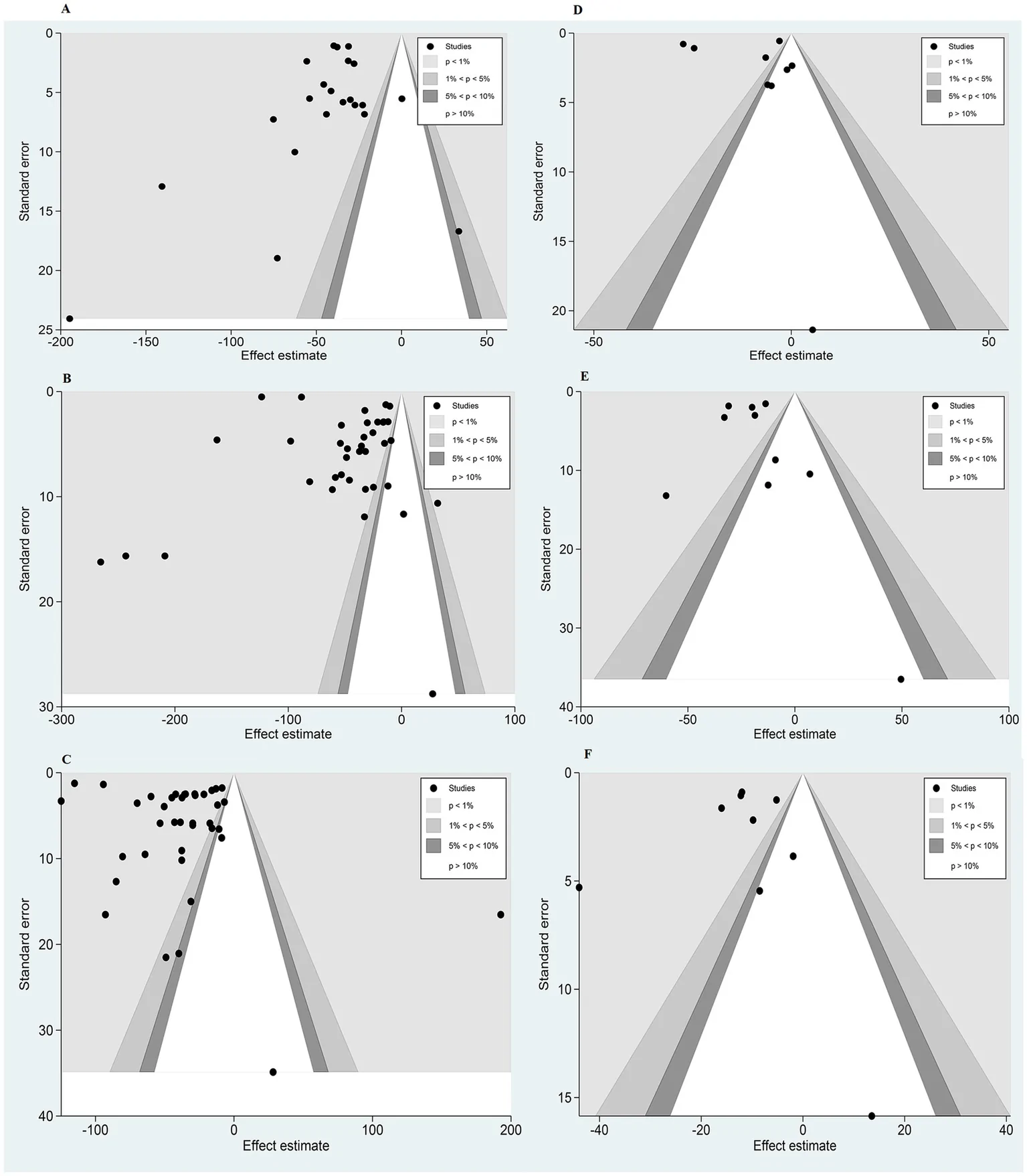
Contour-enhanced funnel plots of meta-analysis for animal and human studies on fasting plasma glucose (FPG), triglycerides (TG) and total cholesterol (TC). Animal data: FPG **(A)**, TG **(B)**, TC **(C)**; Human data: FPG **(D)**, TG **(E)**, TC **(F)**.

## 4 Discussion

This study systematically integrated evidence from both animal experimental studies and human clinical trials associated with MetS, thereby addressing a critical limitation of previous meta-analyses that predominantly focused on human-based research while overlooking the cross-species translational value. Furthermore, a quantitative integrated analysis of mechanism-related biomarkers was conducted in this study to elucidate the underlying biological pathways. To minimize potential confounding factors and enhance the reliability of the accumulated evidence, probiotics derived from dietary sources (e.g., yogurt and milk) were strictly excluded, and only pharmaceutical probiotic preparations with well-defined dosages and standardized formulations were included in the analysis.

Results demonstrated that probiotic and synbiotic interventions effectively ameliorated HbA1c, lipid metabolism, BW, WC, and CRP levels in MetS patients. Meanwhile, in MetS animal models, these interventions significantly improved BW, glycemic metabolism, lipid metabolism, as well as targets including adipokines (adiponectin, leptin), proinflammatory cytokines (CRP, IL-6, IL-1β, TNF-α), metabolic stress signals (FFA, LPS), and liver function parameters (ALT, AST).

The findings of the present study regarding anthropometric indices and lipid metabolism were highly consistent with those reported in the meta-analysis by Liu et al. (2025). Both studies demonstrated statistically significant changes in BW, WC, TC, TG, and HDL-C in the probiotic/synbiotic intervention group, with consistent directional trends. Notably, the present study further confirmed that LDL-C exhibited a statistically significant decrease following the intervention. In contrast, Liu et al. also observed a favorable but non-significant downward trend in LDL-C with probiotic/synbiotic intervention, with a pooled MD of −0.11 (95% CI: −0.24, 0.01).

The findings of the present study regarding glucose metabolism were similar to those reported by Kaul et al. (2024). Both studies demonstrated statistically significant changes in insulin levels in the probiotic/synbiotic intervention group, with consistent directional trends, and neither found significant intergroup differences in the HOMA-IR index. Notably, the present study further confirmed that the intervention could induce statistically significant changes in HbA1c levels. Kaul et al. (2024) also observed a trend toward a reduction in HbA1c levels with probiotic/synbiotic intervention, although this trend was not statistically significant, with a pooled MD of −0.09 (95% CI: −0.23, 0.04).

A study by Kaul et al. (2024) showed that high-dose probiotic intervention with a daily dose exceeding 10^10^ CFU could significantly improve FPG levels compared with the low-dose group. However, no similar dose-dependent difference between the improvement of FPG and probiotic dose was observed in either MetS patients or animal models in this study. This discrepancy may be related to the following factors: first, the limited number of studies and small sample size included in the subgroup analysis led to insufficient statistical power to detect intergroup differences; second, existing evidence indicates that probiotics need to reach a minimum concentration of at least 10^6^ CFU in the intestinal tract to exert effective biological activity, while a large number of viable bacteria are lost during gastrointestinal transit, so most clinical studies adopt higher doses to ensure therapeutic efficacy (Zhai et al., 2021). In the intervention trials included in this study, all probiotic doses were not less than 10^7^ CFU, which generally exceeded the effective concentration threshold; thus, all groups were within the effective therapeutic range, and no significant dose-dependent difference was observed. Although the results of this study showed no significant statistical differences between the high-dose group (>10^9^ CFU) and low-dose group (<10^9^ CFU) of probiotic and synbiotic interventions in terms of glucose metabolism indicators (e.g., insulin, glycated hemoglobin, glucose tolerance), four lipid metabolism parameters (except TG in MetS patients), BW, WC, FFA, and leptin, it was also observed in MetS animal models that the high-dose group could improve the levels of homeostatic model assessment for insulin resistance (HOMA-IR), IL-6, IL-1β, and TNF-α, while no such significant difference was found in the low-dose group, which may be related to the small sample size of the low-dose group, whereas the high-dose group usually included a larger number of studies.

Studies by Kaul et al. (2024) have shown that microbiome therapeutic regimens produce more significant improvements in FPG in patients with a BMI ≥ 30 kg/m^2^ compared with those with a lower baseline BMI. The results of this study demonstrated that both groups of subjects with BMI ≥ 30 kg/m^2^ and 25 ~ 29.9 kg/m^2^ experienced a significant reduction in FPG levels after probiotic or synbiotic intervention, and the absolute MD in FPG reduction in the high BMI group (BMI ≥ 30 kg/m^2^) was approximately 6 times that in the low BMI group (25 ~ 29.9 kg/m^2^). The change trend of TG was consistent with that of FPG, with a more significant improvement effect in the high BMI group after intervention. Among the indicators of insulin, BW, WC, TC, and LDL-C, statistically significant differences were observed in the BMI ≥ 30 kg/m^2^ group after intervention, while no statistically significant differences were found in the BMI 25 ~ 29.9 kg/m^2^ group. Notably, the absolute MD in the latter group was rather higher, which is speculated to be due to the small sample size included in this group, resulting in a wider 95% CI that failed to show statistical significance.

Both Kaul et al. (2024) and Liu et al. (2025) observed significant improvements in lipid profiles in the subgroup of subjects aged <50 years. The results of this study were generally consistent with their findings; specifically, the absolute MD in lipid changes was greater in the <50 years subgroup. TG and HDL-C showed statistically significant differences in the <50 years subgroup but not in the ≥50 years subgroup, whereas TC and LDL-C exhibited statistically significant differences in both the ≥50 years and <50 years subgroups. Notably, in both this study and the studies by Kaul et al. (2024) and Liu et al. (2025), the number of subjects included in the older age subgroup was significantly smaller than that in the younger age subgroup. This insufficient sample size may have affected the stability and reliability of the results.

Kaul et al. (2024) found that compared with trials with an intervention duration of <12 weeks, patients who received continuous intervention for ≥12 weeks showed better efficacy in FPG. A study by Stenman et al. (2014) further confirmed that a longer intervention duration (more than 3 months) is conducive to better control of fat mass and lipid profiles. In contrast, Liu et al. (2025) reported that in the subgroup with an intervention duration of ≥12 weeks, there were no statistically significant differences in WC, TG, and HDL-C, suggesting that a longer intervention cycle may not exert a more significant regulatory effect on body shape and lipid metabolism.

In this study, statistically significant differences in FPG and the four lipid indicators were observed between the <12 weeks and ≥12 weeks intervention subgroups in both animals and humans with MetS. However, the absolute MD of blood glucose indicators was larger in the <12 weeks subgroup than in the ≥12 weeks subgroup, while the absolute MD of lipid indicators was smaller in the <12 weeks subgroup than in the ≥12 weeks subgroup. Meanwhile, in animals with MetS, statistically significant differences in IL-6, IL-1β, TNF-α, and HbA1c were observed in both subgroups, with a larger absolute mean difference in the ≥12 weeks subgroup. This phenomenon may be related to the fact that blood lipids, HbA1c, and inflammatory indicators require long-term treatment to show efficacy, and clinical guidelines usually recommend monitoring blood lipids and glycated hemoglobin every 3 months (Committee ADAPP, 2022).

Previous meta-analyses have suggested that the beneficial effects of probiotics/synbiotics on metabolic parameters in patients with MetS are limited. Hadi et al. (2021) reported that intervention significantly reduced TC levels but showed no significant effects on BW, BMI, WC, FPG, HOMA-IR, insulin, TG, LDL-C, or HDL-C. Dong et al. (2019) only observed a significant reduction in LDL-C levels, with no significant effects on other anthropometric, glucose metabolism, or lipid metabolism parameters. In contrast, a 2025 meta-analysis by Liu et al. (2025) included 24 RCTs covering all RCTs enrolled in the two aforementioned studies, and demonstrated statistically significant differences in all measured outcomes. The inconsistency among these studies indicates that the heterogeneity between subgroup and overall results in the present study may be attributed to the limited number of included studies and small sample sizes, which failed to detect some therapeutic effects. Therefore, the results of subgroup analyses with a small number of included studies should be interpreted with caution, and further validation by large-scale, high-quality clinical trials is warranted.

In patients with MetS, studies investigating the underlying mechanisms of action remain limited. Only one or two RCTs have examined levels of CRP, IL-6, TNF-α, LPS, adiponectin, and leptin, with results indicating a significant reduction solely in CRP, whereas other indicators either lack meta-analytic evidence or show no statistically significant differences.

In animal models of MetS, the present quantitative analysis confirmed that probiotic/synbiotic intervention significantly reduced serum levels of FFA, LPS, leptin, and proinflammatory cytokines including TNF-α, IL-6, IL-1β, and CRP, while increasing adipokine concentrations. Consistent findings were reported by Noormohammadi et al. (2023), who demonstrated decreased leptin levels following probiotic treatment. Statistically, these results may provide reasonable support for the metabolic endotoxemia hypothesis, which proposes that impaired intestinal barrier function leads to LPS translocation into the circulation, thereby activating inflammatory signaling pathways such as TLR4/NF-κB and ultimately exacerbating insulin resistance and metabolic disturbances (Ghosh et al., 2020).

Mechanistically, reduced LPS levels indicate restoration of intestinal barrier function, which is generally regarded as the initial target of probiotic intervention (Ma et al., 2022). Concurrent reductions in TNF-α and IL-6 suggest inhibition of the JNK and IKKβ/NF-κB pathways, which directly contributes to the restoration of insulin signaling (Zhao et al., 2021). The decrease in IL-1β implies suppressed activation of the NLRP3 inflammasome, further alleviating inflammatory cascades (Sanjiwani et al., 2022). Decreased leptin and elevated adipokine levels reflect the effects of probiotics in attenuating lipotoxicity and improving adipose tissue inflammation, which may be mediated via regulation of metabolic pathways including AMPK (Kim et al., 2013).

This study showed that probiotic intervention achieved more pronounced metabolic improvements in MetS animal models than in MetS patients, as reflected by significant reductions in FPG and HOMA-IR among other indicators. Several factors may explain this discrepancy. First, in rodent models, interventions were administered under rigorously controlled conditions, with standardized high-fat diet induction, uniform disease induction, and more severe yet homogeneous baseline metabolic pathology, which maximizes the detectability of a true treatment effect (He et al., 2021). In contrast, human participants typically followed uncontrolled outpatient diets and frequently continued concurrent baseline medications (e.g., metformin or statins), and varied in baseline severity and adherence. These real-world confounders are likely to attenuate and obscure the true effect of probiotics/synbiotics on glycemic metabolism, thereby partly accounting for the overall smaller reductions in FPG, HOMA-IR, and related indicators observed in MetS patients compared with animal models (Setayesh et al., 2026).

Limitations of the present study are as follows. First, some of the included RCTs had relatively small sample sizes, which may compromise the reliability of the conclusions. Secondly, substantial heterogeneity was observed for some outcome indicators, which may be related to the kinds of experimental animals, comorbidities and concomitant medications of participants, intervention duration (4 weeks to several months), probiotic strains, and dosages (10^7^–10^11^ CFU). Subgroup analyses were performed according to intervention duration, mean age of participants, baseline mean body mass index (BMI), and probiotic dosage to explore the sources of heterogeneity. However, high heterogeneity persisted in subgroups for several outcomes, suggesting that heterogeneity was driven by multiple factors. As shown in Table 1, the probiotic strains included in this meta-analysis were highly heterogeneous. Although single strains were commonly adopted in animal studies, there was little overlap of strains across different studies. Most human trials used multi-strain formulations with distinct strain combinations and proportions. These characteristics precluded meaningful subgroup analyses stratified by strain type to trace the sources of heterogeneity. In addition, differences in rodent strains, high-fat diet formulations, and delivery vehicles were other potential sources of heterogeneity. Nevertheless, sensitivity analyses confirmed the robustness of the core findings, indicating an overall consistent beneficial trend of probiotics/synbiotics in improving MetS.

## 5 Conclusion

In summary, probiotic and synbiotic interventions effectively improved BW and lipid metabolism in patients with MetS and animal models. In MetS patients, they lowered HbA1c without affecting FPG, whereas in animal models they improved FPG, HbA1c, insulin, and HOMA-IR. In addition, these interventions exerted a favorable modulatory effect on adipokines (adiponectin, leptin), pro-inflammatory cytokines (CRP, IL-6, IL-1β, TNF-α), and metabolic stress markers. Taken together, these findings suggest that probiotic and synbiotic supplementation represents a promising adjunctive strategy for the management of MetS. However, given the considerable heterogeneity among the included studies and the limited sample sizes, particularly in the subgroup analyses. Therefore, prior to their widespread application, larger-sample, rigorous RCTs are required to define the optimal intervention regimen.

## Data Availability

The original contributions presented in the study are included in the article/Supplementary material, further inquiries can be directed to the corresponding author/s.
