# Supplementary material for: Integrating preclinical and clinical evidence: a meta-analysis of probiotic/synbiotic interventions on metabolic syndrome
Source: Front Microbiol. 2026 Oct 15;17:1995383. doi: 10.3389/fmicb.2026.1995383 (PMC13717699; doi:10.3389/fmicb.2026.1995383)
Supplement: Supplementary file 1 [file Supplementary_file_1.docx]

**Supplementary Material**

**Content**

[Supplementary 1 Search strategy	1](#_Toc22111)

[Supplementary 2 Citations of included studies	9](#_Toc30966)

[Citations for the 10 eligible studies(1-35)	9](#_Toc11996)

[Supplementary 3 Risk of bias assessment for included animal studies (evaluated using the SYRCLE’s tool)	12](#_Toc8863)

[Supplementary 4 Risk of bias assessment for included randomized controlled trials (evaluated using the Cochrane RoB 2.0 tool)	15](#_Toc31654)

[Supplementary 5 Forest plot of meta-analysis for animal and human studies	18](#_Toc13714)

[5.1 Animal studies	18](#_Toc21242)

[5.1.1 HbA1c	18](#_Toc2728)

[5.1.2 Insulin	19](#_Toc8090)

[5.1.3 HOMA-IR	20](#_Toc10692)

[5.1.4 OGTT 0h Blood Glucose	21](#_Toc31093)

[5.1.5 OGTT 2h Blood Glucose	22](#_Toc9286)

[5.1.6 CRP	23](#_Toc26838)

[5.1.7 IL-6	24](#_Toc21522)

[5.1.8 IL-1β	25](#_Toc4039)

[5.1.9 TNF-α	26](#_Toc11223)

[5.1.10 LPS	27](#_Toc21450)

[5.1.11 FFA	28](#_Toc27254)

[5.1.12 Adiponectin	29](#_Toc12823)

[5.1.13 Leptin	30](#_Toc21176)

[5.1.14 ALT	31](#_Toc31481)

[5.1.15 AST	32](#_Toc20924)

[5.2 Human studies	33](#_Toc21773)

[5.2.1 HbA1c	33](#_Toc4543)

[5.2.2 Insulin	34](#_Toc12134)

[5.2.3 HOMA-IR	35](#_Toc9259)

[5.2.4 HOMA-β	36](#_Toc16868)

[5.2.5 CRP	37](#_Toc1292)

[5.2.6 TNF-α	38](#_Toc30024)

[5.2.7 Adiponectin	39](#_Toc2919)

[5.2.8 ALT	40](#_Toc6337)

[5.2.9 AST	41](#_Toc13570)

[Supplementary 6 Results of sensitive analysis for animal and human studies	42](#_Toc14597)

[6.1 Animal studies	42](#_Toc29078)

[6.1.1 Sensitive analysis of FPG	42](#_Toc19779)

[6.1.2 Sensitive analysis of HbA1c	44](#_Toc28816)

[6.1.3 Sensitive analysis of BW	45](#_Toc20574)

[6.1.4 Sensitive analysis of WC	47](#_Toc19906)

[6.1.5 Sensitive analysis of TC	48](#_Toc20827)

[6.1.6 Sensitive analysis of TG	50](#_Toc7349)

[6.1.7 Sensitive analysis of LDL-C	52](#_Toc7143)

[6.1.8 Sensitive analysis of HDL-C	53](#_Toc3966)

[6.1.9 Sensitive analysis of insulin	56](#_Toc25182)

[6.1.10 Sensitive analysis of HOMA-IR	57](#_Toc7491)

[6.1.11 Sensitive analysis of OGTT 0h blood glucose	58](#_Toc23739)

[6.1.12 Sensitive analysis of OGTT 2h blood glucose	59](#_Toc28109)

[6.1.13 Sensitive analysis of CRP	60](#_Toc11352)

[6.1.14 Sensitive analysis of IL-6	61](#_Toc15075)

[6.1.15 Sensitive analysis of IL-1β	62](#_Toc4717)

[6.1.16 Sensitive analysis of TNF-α	63](#_Toc7487)

[6.1.17 Sensitive analysis of LPS	64](#_Toc14244)

[6.1.18 Sensitive analysis of FFA	65](#_Toc10841)

[6.1.19 Sensitive analysis of Leptin	66](#_Toc30018)

[6.1.20 Sensitive analysis of ALT	67](#_Toc12637)

[6.1.21 Sensitive analysis of AST	68](#_Toc21689)

[6.2 Human studies	69](#_Toc6409)

[6.2.1 Sensitive analysis of FPG	69](#_Toc24733)

[6.2.2 Sensitive analysis of HbA1c	70](#_Toc11591)

[6.2.3 Sensitive analysis of BW	71](#_Toc31303)

[6.2.4 Sensitive analysis of WC	72](#_Toc25274)

[6.2.5 Sensitive analysis of TC	73](#_Toc16826)

[6.2.6 Sensitive analysis of TG	74](#_Toc1948)

[6.2.7 Sensitive analysis of LDL-C	75](#_Toc18819)

[6.2.8 Sensitive analysis of HDL-C	76](#_Toc21659)

[6.2.9 Sensitive analysis of insulin	77](#_Toc24223)

[6.2.10 Sensitive analysis of HOMA-IR	78](#_Toc9626)

[6.2.11 Sensitive analysis of ALT	79](#_Toc11454)

[6.2.12 Sensitive analysis of AST	80](#_Toc8579)

[Supplementary 7 Confunnel plots for human and animal studies	81](#_Toc2384)

[7.1 Animal studies	81](#_Toc4487)

[7.1.1 Confunnel plots for HbA1c	81](#_Toc17469)

[7.1.2 Confunnel plots for BW	82](#_Toc27517)

[7.1.3 Confunnel plots for LDL-C	83](#_Toc4931)

[7.1.4 Confunnel plots for HDL-C	84](#_Toc28751)

[7.1.5 Confunnel plots for insulin	85](#_Toc5445)

[7.1.6 Confunnel plots for HOMA-IR	86](#_Toc1668)

[7.1.7 Confunnel plots for OGTT 0h blood glucose	87](#_Toc5395)

[7.1.8 Confunnel plots for OGTT 2h blood glucose	88](#_Toc31342)

[7.1.9 Confunnel plots for CRP	89](#_Toc12149)

[7.1.10 Confunnel plots for IL-6	90](#_Toc22667)

[7.1.11 Confunnel plots for IL-1β	91](#_Toc24184)

[7.1.12 Confunnel plots for TNF-α	92](#_Toc31760)

[7.1.13 Confunnel plots for LPS	93](#_Toc15449)

[7.1.14 Confunnel plots for FFA	94](#_Toc31425)

[7.1.15 Confunnel plots for leptin	95](#_Toc8823)

[7.1.16 Confunnel plots for ALT	96](#_Toc8311)

[7.1.17 Confunnel plots for AST	97](#_Toc11579)

[7.2 Human studies	98](#_Toc18)

[7.2.1 Confunnel plots for BW	98](#_Toc13270)

[7.2.2 Confunnel plots for WC	99](#_Toc3230)

[7.2.3 Confunnel plots for LDL-C	100](#_Toc31548)

[7.2.4 Confunnel plots for HDL-C	101](#_Toc1265)

[7.2.5 Confunnel plots for insulin	102](#_Toc26067)

[7.2.6 Confunnel plots for HOMA-IR	103](#_Toc3356)

[7.2.7 Confunnel plots for ALT	104](#_Toc11684)

[7.2.8 Confunnel plots for AST	105](#_Toc27733)

**Abbreviations**

FPG:Fasting Plasma glucose;

HbA1c:Hemoglobin A1c;

BW:body weight

WC:waist circumference

TC:Total Cholesterol;

LDL-C:Low-density lipoprotein cholesterol;

TG:Triglycerides;

HDL-C:High-density lipoprotein cholesterol ;

HOMA-IR:Homeostasis model assessment of insulin resistance;

HOMA-β:Homeostasis model assessment of β-cell function;

OGTT:Oral Glucose Tolerance Test;

CRP:C-reactive protein;

IL-6:Interleukin-6;

IL-1β:Interleukin-1 beta;

TNF-α:Tumor Necrosis Factor-alpha;

LPS:Lipopolysaccharides;

FFA:Free fatty acids ;

ALT:Alanine aminotransferase activity ;

AST:Aspartate aminotransferase activity

# Supplementary 1 Search strategy

**Search strategy**

Pubmed, Embase , the Cochrane Controlled Register of Trials, ClinicalTrials.gov, CNKI (China National Knowledge Infrastructure) and Wanfang Data Knowledge Service Platform from database inception to June 10, 2025.

The detailed search strategy for each database was presented below:

**Pubmed**

**Participation (****Metabolic Syndrome) related**

#1 "Metabolic Syndrome"[MeSH Terms]

#2 "Insulin Resistance"[MeSH Terms]

#3 "Obesity, Abdominal"[MeSH Terms]

#4 "Metabolic Syndromes"[Title/Abstract]

#5 "Syndrome, Metabolic"[Title/Abstract]

#6 "Syndromes, Metabolic"[Title/Abstract]

#7 "Syndrome X, Reaven"[Title/Abstract]

#8 "Metabolic Syndrome X"[Title/Abstract]

#9 "Insulin Resistance Syndrome X"[Title/Abstract]

#10 "Metabolic Cardiovascular Syndrome"[Title/Abstract]

#11 "Syndrome X, Insulin Resistance"[Title/Abstract]

#12 "Metabolic X Syndrome"[Title/Abstract]

#13 "Syndrome X, Metabolic"[Title/Abstract]

#14 "Cardiometabolic Syndrome"[Title/Abstract]

#15 "Cardiometabolic Syndromes"[Title/Abstract]

#16 "Syndrome, Cardiometabolic"[Title/Abstract]

#17 "Resistance, Insulin"[Title/Abstract]

#18 "Insulin Sensitivity"[Title/Abstract]

#19 "Sensitivity, Insulin"[Title/Abstract]

#20 "Abdominal Obesities"[Title/Abstract]

#21 "Abdominal Obesity"[Title/Abstract]

#22 "Central Obesity"[Title/Abstract]

#23 "Obesity, Central"[Title/Abstract]

#24 "Obesity, Visceral"[Title/Abstract]

#25 "Visceral Obesity"[Title/Abstract]

#26 OR #1-25

**Intervention (Probiotics) related**

#27 "Probiotics"[MeSH Terms]

#28 "Lactobacillus"[MeSH Terms]

#29 "[Lactobacillus acidophilus](https://www.ncbi.nlm.nih.gov/mesh/68007779)"[MeSH Terms]
#30 "[Lactobacillus crispatus](https://www.ncbi.nlm.nih.gov/mesh/2016205)"[MeSH Terms]

#31 "[Lactobacillus delbrueckii](https://www.ncbi.nlm.nih.gov/mesh/68048668)"[MeSH Terms]

\#32"[Lactobacillus gasseri](https://www.ncbi.nlm.nih.gov/mesh/2016200)"[MeSH Terms]

#33 "[Lactobacillus helveticus](https://www.ncbi.nlm.nih.gov/mesh/68052198)"[MeSH Terms]

#34 "[Lactobacillus plantarum](https://www.ncbi.nlm.nih.gov/mesh/68048191)"[MeSH Terms]

#35 "[Saccharomyces boulardii](https://www.ncbi.nlm.nih.gov/mesh/2016195)"[MeSH Terms]

#36 "Clostridium butyricum"[MeSH Terms]

#37 "Streptococcus thermophilus"[MeSH Terms]

#38 "Bifidobacterium"[MeSH Terms]

#39 "[Bifidobacterium adolescentis](https://www.ncbi.nlm.nih.gov/mesh/2016207)"[MeSH Terms]

#40 "[Bifidobacterium animalis](https://www.ncbi.nlm.nih.gov/mesh/2016194)"[MeSH Terms]

#41 "[Bifidobacterium bifidum](https://www.ncbi.nlm.nih.gov/mesh/2016197)"[MeSH Terms]

#42 "[Bifidobacterium breve](https://www.ncbi.nlm.nih.gov/mesh/2016196)"[MeSH Terms]

#43 "[Bifidobacterium longum](https://www.ncbi.nlm.nih.gov/mesh/2016190)"[MeSH Terms]

#44 "[Bifidobacterium longum subspecies infantis](https://www.ncbi.nlm.nih.gov/mesh/2016236)"[MeSH Terms]

#45 "[Bifidobacterium pseudocatenulatum](https://www.ncbi.nlm.nih.gov/mesh/2016238)"[MeSH Terms]

#46 "[Leuconostoc mesenteroides](https://www.ncbi.nlm.nih.gov/mesh/2016209)"[MeSH Terms]

#47 "[Escherichia coli K12](https://www.ncbi.nlm.nih.gov/mesh/68048168)"[MeSH Terms]

#48 "[Bacillus amyloliquefaciens](https://www.ncbi.nlm.nih.gov/mesh/2016189)"[MeSH Terms]

#49 "[Bacillus clausii](https://www.ncbi.nlm.nih.gov/mesh/2016213)"[MeSH Terms]

#50 "[Bacillus coagulans](https://www.ncbi.nlm.nih.gov/mesh/2016202)"[MeSH Terms]

#51 "[Bacillus subtilis](https://www.ncbi.nlm.nih.gov/mesh/68001412)"[MeSH Terms]

#52 "[Lacticaseibacillus](https://www.ncbi.nlm.nih.gov/mesh/2103236)"[MeSH Terms]

#53 "Lacticaseibacillus paracasei"[MeSH Terms]

#54 "[Synbiotics](https://www.ncbi.nlm.nih.gov/mesh/68058616)"[Title/Abstract]

#55 "Lactobacillus amylovorus"[Title/Abstract]

#56 "Lactobacillus bulgaricus"[Title/Abstract]

#57 "Lactobacillus delbrueckii subsp. bulgaricus"[Title/Abstract]

#58 "Lactobacillus delbrueckii subspecies bulgaricus"[Title/Abstract]

#59 "boulardii, Saccharomyces"[Title/Abstract]

#60 "Bifidobacterium animalis subsp lactis"[Title/Abstract]

#61 "Bifidobacterium lactis"[Title/Abstract]

#62 "Bifidobacterium BB-12"[Title/Abstract]

#63 "Bifidobacterium infantis"[Title/Abstract]

#64 "Bifidobacterium longum ssp. infantis"[Title/Abstract]

#65 "Lactic acid bacteria"[Title/Abstract]

#66 OR #27-65

#67 #26 AND #66

**Studies (randomized controlled trial) related**

#68

(randomized controlled trial[pt] OR controlled clinical trial[pt] OR randomized[tiab] OR placebo[tiab] OR drug therapy[sh] OR randomly[tiab] OR trial[tiab] OR groups[tiab]) NOT (animals[mh] NOT humans[mh])

#169 #67AND #68

**EMBASE**

**Participation (Metabolic Syndrome) related**

#1 'metabolic syndrome x'/de

#2 'insulin resistance'/de

#3 'abdominal obesity'/de

#4 'Metabolic Syndromes':ti,ab,kw

#5 'Syndrome, Metabolic':ti,ab,kw

#6 'Syndromes, Metabolic':ti,ab,kw

#7 'Syndrome X, Reaven':ti,ab,kw

#8 'Metabolic Syndrome X':ti,ab,kw

#9 'Insulin Resistance Syndrome X':ti,ab,kw

#10 'Metabolic Cardiovascular Syndrome':ti,ab,kw

#11 'Syndrome X, Insulin Resistance':ti,ab,kw

#12 'Metabolic X Syndrome':ti,ab,kw

#13 'Syndrome X, Metabolic':ti,ab,kw

#14 'Cardiometabolic Syndrome':ti,ab,kw

#15 'Cardiometabolic Syndromes':ti,ab,kw

#16 'Syndrome, Cardiometabolic':ti,ab,kw

#17 'Resistance, Insulin':ti,ab,kw

#18 'Insulin Sensitivity':ti,ab,kw

#19 'Sensitivity, Insulin':ti,ab,kw

#20 'Abdominal Obesities':ti,ab,kw

#21 'Abdominal Obesity':ti,ab,kw

#22 'Central Obesity':ti,ab,kw

#23 'Obesity, Central':ti,ab,kw

#24 'Obesity, Visceral':ti,ab,kw

#25 'Visceral Obesity':ti,ab,kw

#26 OR#1-25

**Intervention (Probiotics) related**

#27 'probiotic agent'/de

#28 'Lactobacillus'/de

#29 'Lactobacillus acidophilus'/de

#30 'Lactobacillus crispatus'/de

#31 'Lactobacillus delbrueckii'/de

#32 'Lactobacillus gasseri'/de

#33 'Lactobacillus helveticus'/de

#34 'Lactobacillus plantarum'/de

#35 'Saccharomyces boulardii'/de

#36 'Clostridium butyricum'/de

#37 'Streptococcus thermophilus'/de

#38 'Bifidobacterium'/de

#39 'Bifidobacterium adolescentis'/de

#40 'Bifidobacterium animalis'/de

#41 'Bifidobacterium bifidum'/de

#42 'Bifidobacterium breve'/de

#43 'Bifidobacterium longum'/de

#44 'Bifidobacterium longum subsp. infantis'/de

#45 'Bifidobacterium pseudocatenulatum'/de

#46 'Leuconostoc mesenteroides'/de

#47 'Escherichia coli K-12'/de

#48 'Bacillus amyloliquefaciens'/de

#49 'Bacillus clausii'/de

#50 'Bacillus coagulans'/de

#51 'Bacillus subtilis'/de

#52 'Lacticaseibacillus'/de

#53 'Lactobacillus paracasei'/de

#54 '[Synbiotics](https://www.ncbi.nlm.nih.gov/mesh/68058616)':ti,ab,kw

#55 'Lactobacillus amylovorus':ti,ab,kw

#56 'Lactobacillus bulgaricus':ti,ab,kw

#57 'Lactobacillus delbrueckii subsp. bulgaricus':ti,ab,kw

#58 'Lactobacillus delbrueckii subspecies bulgaricus':ti,ab,kw

#59 'boulardii, Saccharomyces':ti,ab,kw

#60 'Bifidobacterium animalis subsp lactis':ti,ab,kw

#61 'Bifidobacterium lactis':ti,ab,kw

#62 'Bifidobacterium BB-12':ti,ab,kw

#63 'Bifidobacterium infantis':ti,ab,kw

#64 'Bifidobacterium longum ssp. infantis':ti,ab,kw

#65 'Lactic acid bacteria':ti,ab,kw

#66 OR #27-65

#67 #26 AND #66

**Studies (randomized controlled trial) related**

#68

'crossover procedure':de OR 'double-blind procedure':de OR 'randomized controlled trial':de OR 'single-blind procedure':de OR (random* OR factorial* OR crossover* OR cross NEXT/1 over* OR placebo* OR doubl* NEAR/1 blind* OR singl* NEAR/1 blind* OR assign* OR allocat* OR volunteer*):de,ab,ti

#69 #67AND #68

**Cochrane**

**Participation (Metabolic Syndrome) related**

#1 MeSH descriptor: [Metabolic Syndrome] this term only

#2 MeSH descriptor: [Insulin Resistance] this term only

#3 MeSH descriptor: [Obesity, Abdominal] this term only

#4 ("Metabolic Syndromes"):ti,ab,kw

#5 ("Syndrome, Metabolic"):ti,ab,kw

#6 ("Syndromes, Metabolic"):ti,ab,kw

#7 ("Syndrome X, Reaven"):ti,ab,kw

#8 ("Metabolic Syndrome X"):ti,ab,kw

#9 ("Insulin Resistance Syndrome X"):ti,ab,kw

#10 ("Metabolic Cardiovascular Syndrome"):ti,ab,kw

#11 ("Syndrome X, Insulin Resistance"):ti,ab,kw

#12 ("Metabolic X Syndrome"):ti,ab,kw

#13 ("Syndrome X, Metabolic"):ti,ab,kw

#14 ("Cardiometabolic Syndrome"):ti,ab,kw

#15 ("Cardiometabolic Syndromes"):ti,ab,kw

#16 ("Syndrome, Cardiometabolic"):ti,ab,kw

#17 ("Resistance, Insulin"):ti,ab,kw

#18 ("Insulin Sensitivity"):ti,ab,kw

#19 ("Sensitivity, Insulin"):ti,ab,kw

#20 ("Abdominal Obesities"):ti,ab,kw

#21 ("Abdominal Obesity"):ti,ab,kw

#22 ("Central Obesity"):ti,ab,kw

#23 ("Obesity, Central"):ti,ab,kw

#24 ("Obesity, Visceral"):ti,ab,kw

#25 ("Visceral Obesity"):ti,ab,kw

#26 OR #1-25

**Intervention (Probiotics) related**

#27 MeSH descriptor: [Probiotics] this term only

#28 MeSH descriptor: [Lactobacillus] this term only

#29 MeSH descriptor: [[Lactobacillus acidophilus](https://www.ncbi.nlm.nih.gov/mesh/68007779)] this term only
#30 MeSH descriptor: [[Lactobacillus crispatus](https://www.ncbi.nlm.nih.gov/mesh/2016205)] this term only

#31 MeSH descriptor: [[Lactobacillus delbrueckii](https://www.ncbi.nlm.nih.gov/mesh/68048668)] this term only

#32 MeSH descriptor: [[Lactobacillus gasseri](https://www.ncbi.nlm.nih.gov/mesh/2016200)] this term only

#33 MeSH descriptor: [[Lactobacillus helveticus](https://www.ncbi.nlm.nih.gov/mesh/68052198)] this term only

#34 MeSH descriptor: [[Lactobacillus plantarum](https://www.ncbi.nlm.nih.gov/mesh/68048191)] this term only

#35 MeSH descriptor: [[Saccharomyces boulardii](https://www.ncbi.nlm.nih.gov/mesh/2016195)] this term only

#36 MeSH descriptor: [Clostridium butyricum] this term only

#37 MeSH descriptor: [Streptococcus thermophilus] this term only

#38 MeSH descriptor: [Bifidobacterium] this term only

#39 MeSH descriptor: [[Bifidobacterium bifidum](https://www.ncbi.nlm.nih.gov/mesh/2016197)] this term only

#40 MeSH descriptor: [[Bifidobacterium breve](https://www.ncbi.nlm.nih.gov/mesh/2016196)] this term only

#41 MeSH descriptor: [[Bifidobacterium longum](https://www.ncbi.nlm.nih.gov/mesh/2016190)] this term only

#42 MeSH descriptor: [[Bifidobacterium longum subspecies infantis](https://www.ncbi.nlm.nih.gov/mesh/2016236)] this term only

#43 MeSH descriptor: [[Bacillus clausii](https://www.ncbi.nlm.nih.gov/mesh/2016213)] this term only

#44 MeSH descriptor: [[Bacillus coagulans](https://www.ncbi.nlm.nih.gov/mesh/2016202)] this term only

#45 MeSH descriptor: [[Bacillus subtilis](https://www.ncbi.nlm.nih.gov/mesh/68001412)] this term only

#46 MeSH descriptor: [[Lacticaseibacillus](https://www.ncbi.nlm.nih.gov/mesh/2103236)] this term only

#47 MeSH descriptor: [Lacticaseibacillus paracasei] this term only

#48 ("[Synbiotics](https://www.ncbi.nlm.nih.gov/mesh/68058616)"):ti,ab,kw

#49 ("Lactobacillus amylovorus"):ti,ab,kw

#50 ("Lactobacillus bulgaricus"):ti,ab,kw

#51 ("Lactobacillus delbrueckii subsp. bulgaricus"):ti,ab,kw

#52 ("Lactobacillus delbrueckii subspecies bulgaricus"):ti,ab,kw

#53 ("boulardii, Saccharomyces"):ti,ab,kw

#54 ("Bifidobacterium animalis subsp lactis"):ti,ab,kw

#55 ("Bifidobacterium lactis"):ti,ab,kw

#56 ("Bifidobacterium BB-12"):ti,ab,kw

#57 ("Bifidobacterium infantis"):ti,ab,kw

#58 ("Bifidobacterium longum ssp. infantis"):ti,ab,kw

#59 ("Lactic acid bacteria"):ti,ab,kw

#60 OR #27-59

#61 #26 AND #60

**Studies (randomized controlled trial) related**

#62 MeSH descriptor: [Randomized Controlled Trials as Topic] this term only

#63 ("randomized controlled trial"):ti,ab,kw

#64 ("randomized control trial"):ti,ab,kw

#65 ("randomized controlled trials"):ti,ab,kw

#66 ("rct"):ti,ab,kw

#67 ("rcts"):ti,ab,kw

#68 ("randomized"):ti,ab,kw

#69 ("placebo"):ti,ab,kw

#70 ("randomly"):ti,ab,kw

#71 OR #62-70

#72 #61 AND #71

**ClinicalTrials.gov**

#1 Condition or disease: Metabolic Syndrome

#2 Study Results: Studies With results

#3 Intervention/treatment: Probiotics

**CNKI, Wanfang Data Knowledge Service Platform**

The following are the corresponding English translations of the Chinese subject terms：

**Participants (Metabolic Syndrome) related**

Metabolic syndrome

Insulin resistance

Insulin sensitivity

Abdominal obesity / Central obesity

Visceral fat obesity / Visceral obesity

Cardiometabolic syndrome

Reaven syndrome

Syndrome X

Dysmetabolic syndrome

Insulin resistance syndrome

Cardiovascular syndrome

Metabolic syndrome X

**Intervention (Probiotics) related**

Beneficial bacteria

Beneficial active microorganisms

Bifidobacterium

Lactic acid bacteria

Actinobacteria

Probiotic supplement

Gut microbiota

Probiotic preparation

Live bacterial preparation

Yeast

Prebiotic

Probiotic

Lactobacillus acidophilus

Lactobacillus crispatus

Lactobacillus bulgaricus

Lactobacillus plantarum

Lactobacillus gasseri

Lactobacillus helveticus

Lactobacillus casei

Lactobacillus paracasei

Bifidobacterium

Bifidobacterium adolescentis

Bifidobacterium animalis

Bifidobacterium bifidum

Bifidobacterium breve

Bifidobacterium longum

Bifidobacterium infantis

BB-12

Saccharomyces cerevisiae

Saccharomyces boulardii

Clostridium butyricum

Streptococcus thermophilus

Bacillus subtilis

Bacillus coagulans

Bacillus coagulans

Bacillus amyloliquefaciens

Bacillus licheniformis

Synbiotic

Clostridium butyricum

Bacillus cereus

**Studies (randomized controlled trial) related**

randomized controlled trial

RCT

RCTs

**Supplementary 2 Citations of included studies**

**Citations for the 10 eligible studies(1-35)**

1.	Yang J, Huang J, Huang Z, et al. Cardiometabolic benefits of Lacticaseibacillus paracasei 8700: 2: a randomized double-blind placebo-controlled trial. Clinical nutrition. 2023;42(9):1637‐1646.

2.	Rabiei S, Shakerhosseini R, Saadat N. The effects of symbiotic therapy on anthropometric measures, body composition and blood pressure in patient with metabolic syndrome: a triple blind RCT. Med J Islam Repub Iran. 2015;29:213.

3.	Rabiei S, Hedayati M, Rashidkhani B, Saadat N, Shakerhossini R. The Effects of Synbiotic Supplementation on Body Mass Index, Metabolic and Inflammatory Biomarkers, and Appetite in Patients with Metabolic Syndrome: A Triple-Blind Randomized Controlled Trial. J Diet Suppl. 2019;16(3):294-306.

4.	Eslamparast T, Zamani F, Hekmatdoost A, et al. Effects of synbiotic supplementation on insulin resistance in subjects with the metabolic syndrome: a randomised, double-blind, placebo-controlled pilot study. Br J Nutr. 2014;112(3):438-45.

5.	Parastouei K, Saeidipoor S, Sepandi M, Abbaszadeh S, Taghdir M. Effects of synbiotic supplementation on the components of metabolic syndrome in military personnel: a double-blind randomised controlled trial. BMJ Mil Health. 2022;168(5):362-367.

6.	Luangphiphat W, Prombutara P, Jamjuree P, et al. The efficacy of Lacticaseibacillus paracasei MSMC39-1 and Bifidobacterium animalis TA-1 probiotics in modulating gut microbiota and reducing the risk of the characteristics of metabolic syndrome: A randomized, double-blinded, placebo-controlled study. PLoS One. 2025;20(1):e0317202.

7.	Rahimi F, Pasdar Y, Kaviani M, et al. Efficacy of the Synbiotic Supplementation on the Metabolic Factors in Patients with Metabolic Syndrome: a Randomized, Triple-Blind, Placebo-Controlled Trial. International journal of clinical practice. 2022;2022:2967977.

8.	Cicero AFG, Fogacci F, Bove M, Giovannini M, Borghi C. Impact of a short-term synbiotic supplementation on metabolic syndrome and systemic inflammation in elderly patients: a randomized placebo-controlled clinical trial. Eur J Nutr. 2021;60(2):655-663.

9.	Kathiya MD, Parmar TN, Patel DJ, Damor NM. Impact of Gut Microbiota Modulation on Inflammation and Cardiovascular Risk in Patients with Metabolic Syndrome. European Journal of Cardiovascular Medicine. 2025;15(3):733-736.

10.	Mario Gorenjak, Dejan Škorjanc, Skok P, Mechanisms of Action of Probiotic and Protective Bacteria PCS 20, PCS 26, and LGG in Prevention of the Metabolic Syndrome. 2014.

11.	Wastyk HC, Perelman D, Topf M, et al. Randomized controlled trial demonstrates response to a probiotic intervention for metabolic syndrome that may correspond to diet. Gut Microbes. 2023;15(1):2178794.

12.	Depommier C, Everard A, Druart C, et al. Supplementation with Akkermansia muciniphila in overweight and obese human volunteers: a proof-of-concept exploratory study. Nat Med. 2019;25(7):1096-1103.

13.	Wu CS, Lin CC, Hsieh FC, Wu TY, Fang AH. Antiobesity Effect of Lacticaseibacillus paracasei LM-141 on High-Fat Diet-Induced Rats through Alleviation of Inflammation and Insulin Resistance. Evidence-based Complementary and Alternative Medicine. 2023;2023.

14.	Sun R, Li D, Sun M, et al. Bacillus natto ameliorates obesity by regulating PI3K/AKT pathways in rats. Biochemical and Biophysical Research Communications. 2022;603:160-166.

15.	Cano PG, Santacruz A, Trejo FM, Sanz Y. Bifidobacterium CECT 7765 improves metabolic and immunological alterations associated with obesity in high-fat diet-fed mice. Obesity (Silver Spring). 2013;21(11):2310-21.

16.	Moya-Pérez A, Neef A, Sanz Y. Bifidobacterium pseudocatenulatum CECT 7765 Reduces Obesity-Associated Inflammation by Restoring the Lymphocyte-Macrophage Balance and Gut Microbiota Structure in High-Fat Diet-Fed Mice. PLoS One. 2015;10(7):e0126976.

17.	Chen JJ, Zheng RF, Mo WB, Li GF, ML L. Changes in glycolipid metabolism, oxidative stress, and myogenic differentiation levels of skeletal muscle satellite cells in rats with type 2 diabetes mellitus treated with exercise combined with probiotics. Acta Laboratorium Animalis Scientia Sinica. 2024;32(09):1171-1181.

18.	Yi JD, Chang XY, Wang XL, YJ C. Effect of probiotics interfering with the expression of DAG-PKC in liver tissue on insulin resistance in obese mice. Herald of Medicine. 2021;40(08):1017-1021.

19.	Zheng HY, Lan H, Zhuang YY, HW L. Effects of Bifidobacterium CP-9 and Lactobacillus reuteri on glucose and lipid metabolism in obese mice. Acta Nutrimenta Sinica. 2021;43(3):283-288.

20.	Oh YJ, Kim HJ, Kim TS, Yeo IH, Ji GE. Effects of Lactobacillus plantarum PMO 08 Alone and Combined with Chia Seeds on Metabolic Syndrome and Parameters Related to Gut Health in High-Fat Diet-Induced Obese Mice. J Med Food. 2019;22(12):1199-1207.

21.	Miao Z, Zheng H, Liu WH, et al. Lacticaseibacillus paracasei K56 Attenuates High-Fat Diet-Induced Obesity by Modulating the Gut Microbiota in Mice. Probiotics Antimicrob Proteins. 2023;15(4):844-855.

22.	Song W, Song C, Li L, et al. Lactobacillus alleviated obesity induced by high-fat diet in mice. J Food Sci. 2021;86(12):5439-5451.

23.	Lin YK, Lin YH, Chiang CF, Yeh TM, Shih WL. Lactobacillus delbrueckii subsp. bulgaricus strain TCI904 reduces body weight gain, modulates immune response, improves metabolism and anxiety in high fat diet-induced obese mice. 3 Biotech. 2022;12(12).

24.	Jiang S, Liu A, Ma W, et al. Lactobacillus gasseri CKCC1913 mediated modulation of the gut-liver axis alleviated insulin resistance and liver damage induced by type 2 diabetes. Food Funct. 2023;14(18):8504-8520.

25.	Lee E, Jung SR, Lee SY, et al. Lactobacillus plantarum Strain Ln4 Attenuates Diet-Induced Obesity, Insulin Resistance, and Changes in Hepatic mRNA Levels Associated with Glucose and Lipid Metabolism. Nutrients. 2018;10(5).

26.	Osman A, El-Gazzar N, Almanaa TN, El-Hadary A, Sitohy M. Lipolytic Postbiotic from Lactobacillus paracasei Manages Metabolic Syndrome in Albino Wistar Rats. Molecules. 2021;26(2).

27.	Huang E, Kim S, Park H, et al. Modulation of the Gut Microbiome and Obesity Biomarkers by Lactobacillus Plantarum KC28 in a Diet-Induced Obesity Murine Model. Probiotics and Antimicrobial Proteins. 2021;13(3):677-697.

28.	Zhang L, Gao L, Wang C, et al. Pediococcus acidilactici AS185 improves metabolic syndrome induced by a high-fat and high-fructose diet. Food Science. 2021;42(1):215-221.

29.	Yang M, Wang JH, Shin JH, et al. Pharmaceutical efficacy of novel human-origin Faecalibacterium prausnitzii strains on high-fat-diet-induced obesity and associated metabolic disorders in mice. Frontiers in Endocrinology. 2023;14.

30.	Cavalcante RGS, de Albuquerque TMR, de Luna Freire MO, et al. The probiotic Lactobacillus fermentum 296 attenuates cardiometabolic disorders in high fat diet-treated rats. Nutr Metab Cardiovasc Dis. 2019;29(12):1408-1417.

31.	Yu RQ, Yuan JL, Ma LY, Qin QX, XY W. Probiotics improve obesity-associated dyslipidemia and insulin resistance in high-fat diet-fed rats. Chinese Journal of Contemporary Pediatrics. 2013;15(12):1123-1127.

32.	Yang XH, Hong JQ, Wang LH, et al. Screening and Evaluation of Lactic Acid Bacteria for Hypoglycemic Effect in Type 2 Diabetic Mellitus Mice. Food Science. 2025;46(08):151-161.

33.	Yang G, Zhang L, Wang C, et al. Screening and Evaluation of Lactobacillus Strains for Improving Metabolic Syndrome. Modern Food Science and Technology. 2020;36(11):54-60.

34.	Sharma N, Navik U, Tikoo K. Unveiling the presence of epigenetic mark by Lactobacillus supplementation in high-fat diet-induced metabolic disorder in Sprague-Dawley rats. J Nutr Biochem. 2020;84:108442.

35.	Silva GA, Moreira ALG, Silva PHF, et al. The use of probiotics can reduce the severity of experimental periodontitis in rats with metabolic syndrome: An immunoenzymatic and microtomographic study. J Periodontol. 2022;93(2):e1-e12.

**Supplementary 3 Risk of bias assessment for included animal studies (evaluated using the SYRCLE’s tool)**

**Table S3.1 The results of risk of bias assessment for included animal studies using the SYRCLE’s tool**

| Study | Selection bias | | | Performance bias | | Detection bias | | Attrition bias | Reporting bias | Other |
| --- | --- | --- | --- | --- | --- | --- | --- | --- | --- | --- |
|  | Sequence generation | Baseline characteristics | Allocation concealment | Random housing | Blinding | Random outcome assessment | Blinding | Incomplete outcome data | Selective outcome reporting | Other sources of bias |
| Zhang HY 2021 | Yes | Yes | Unclear | Unclear | Unclear | Unclear | Unclear | Yes | Yes | Yes |
| Osman A 2021 | Unclear | Yes | Unclear | Unclear | Unclear | Unclear | Unclear | Yes | Yes | Yes |
| Yang G 2020 | Unclear | Yes | Unclear | Unclear | Unclear | Unclear | Unclear | Yes | Yes | Yes |
| Zhang L 2021 | Unclear | Yes | Unclear | Unclear | Unclear | Unclear | Unclear | Yes | Yes | Yes |
| Yu RQ 2013 | Unclear | Yes | Unclear | Unclear | Unclear | Unclear | Unclear | Yes | Yes | Yes |
| Yi JD 2021 | Yes | Yes | Unclear | Unclear | Unclear | Unclear | Unclear | Yes | Yes | Yes |
| Chen JJ 2024 | Yes | Yes | Unclear | Unclear | Unclear | Unclear | Unclear | Yes | Yes | Yes |
| Wu CS 2023 | Unclear | Yes | Unclear | Unclear | Unclear | Unclear | Unclear | Yes | Yes | Yes |
| Cano PG 2013 | Unclear | Yes | Unclear | Unclear | Yes | Unclear | Yes | Yes | Yes | Yes |
| Moya-Pérez A 2015 | Unclear | Yes | Unclear | Unclear | Yes | Unclear | Yes | Yes | Yes | Yes |
| Oh YJ 2019 | Unclear | Yes | Unclear | Unclear | Unclear | Unclear | Unclear | Yes | Yes | Yes |
| Miao ZH 2022 | Yes | Yes | Unclear | Unclear | Unclear | Unclear | Unclear | Yes | Yes | Yes |
| Lin YK 2022 | Yes | Yes | Unclear | Unclear | Unclear | Unclear | Unclear | Yes | Yes | Yes |
| Lee E 2018 | Unclear | Yes | Unclear | Unclear | Unclear | Unclear | Unclear | Yes | Yes | Yes |
| Huang E 2020 | Unclear | Yes | Unclear | Unclear | Unclear | Unclear | Unclear | Yes | Yes | Yes |
| Yang M 2023 | Unclear | Yes | Unclear | Unclear | Unclear | Unclear | Unclear | Yes | Yes | Yes |
| Cavalcante RGS 2019 | Unclear | Yes | Unclear | Unclear | Unclear | Unclear | Unclear | Yes | Yes | Yes |
| Sharma N 2020 | Unclear | Yes | Unclear | Unclear | Unclear | Unclear | Unclear | Yes | Yes | Yes |
| Silva GA 2022 | Unclear | Yes | Unclear | Unclear | Unclear | Unclear | Unclear | Yes | Yes | Yes |
| Jiang SM 2023 | Unclear | Yes | Unclear | Unclear | Unclear | Unclear | Unclear | Yes | Yes | Yes |
| Song W 2021 | Unclear | Yes | Unclear | Unclear | Unclear | Unclear | Unclear | Yes | Yes | Yes |
| Sun RY 2022 | Unclear | Yes | Unclear | Unclear | Unclear | Unclear | Unclear | Yes | Yes | Yes |
| Yang XH 2020 | Unclear | Yes | Unclear | Unclear | Unclear | Unclear | Unclear | Yes | Yes | Yes |

Evaluation results of the SYRCLE's tool

|  | Yes | Unclear | No |
| --- | --- | --- | --- |
| Sequence generation | 5 | 18 | 0 |
| Baseline characteristics | 23 | 0 | 0 |
| Allocation concealment | 0 | 23 | 0 |
| Random housing | 0 | 23 | 0 |
| Performance bias-Blinding | 2 | 21 | 0 |
| Random outcome assessment | 0 | 23 | 0 |
| Detection bias-Blinding | 2 | 21 | 0 |
| Incomplete outcome data | 23 | 0 | 0 |
| Selective outcome reporting | 23 | 0 | 0 |
| Other sources of bias | 23 | 0 | 0 |

**Figure S3.1 Summary of risk of bias assessment for included animal studies using the SYRCLE’s tool**

**Supplementary 4 Risk of bias assessment for included randomized controlled trials (evaluated using the Cochrane RoB 2.0 tool)**

**Table S4.1 The results of risk of bias assessment for included randomized controlled trials using the Cochrane RoB 2.0 tool**

| **Study** | Randomization process | Deviations from intended interventions | Missing outcome data | Measurement of the outcome | Selection of the reported result | Overall |  |  |
| --- | --- | --- | --- | --- | --- | --- | --- | --- |
| Yang JL 2023 |  |  |  |  |  |  |  | Low risk |
| Gorenjak M 2015 |  |  |  |  |  |  |  | Some concerns |
| Luangphiphat W 2025 |  |  |  |  |  |  |  | High risk |
| Kathiya MD 2025 |  |  |  |  |  |  |  |  |
| Depommier C 2020 |  |  |  |  |  |  |  |  |
| Rabiei S 2015 |  |  |  |  |  |  |  |  |
| Rabiei S 2018 |  |  |  |  |  |  |  |  |
| Eslamparast T 2014 |  |  |  |  |  |  |  |  |
| Parastouei K 2020 |  |  |  |  |  |  |  |  |
| Rahimi F 2022 |  |  |  |  |  |  |  |  |
| Cicero AFG 2020 |  |  |  |  |  |  |  |  |
| Wastyk HC 2023 |  |  |  |  |  |  |  |  |

As percentage (intention-to-treat)

|  | Low risk | Some concerns | High risk |
| --- | --- | --- | --- |
| Randomization process | 41.7 | 58.3 | 0 |
| Deviations from intended interventions | 100 | 0 | 0 |
| Mising outcome data | 100 | 0 | 0 |
| Measurement of the outcome | 100 | 0 | 0 |
| Selection of the reported result | 100 | 0 | 0 |
| Overall Bias | 41.7 | 58.3 | 0 |

**Figure S4.1 Summary of risk of bias assessment for included randomized controlled trials using the Cochrane RoB 2.0 tool**

# Supplementary 5 Forest plot of meta-analysis for animal and human studies

# 5.1 Animal studies

# 5.1.1 HbA1c

**Figure S5.1.1 Forest plot of HbA1c**

# **5.1.2 Insulin**

**Figure S5.1.2 Forest plot of insulin**

# 5.1.3 HOMA-IR

**Figure S5.1.3 Forest plot of HOMA-IR**

# 5.1.4 OGTT 0h Blood Glucose

**Figure S5.1.4 Forest plot of OGTT 0h blood glucose**

# 5.1.5 OGTT 2h Blood Glucose

**Figure S5.1.5 Forest plot of OGTT 2h blood glucose**

# 5.1.6 CRP

**Figure S5.1.6 Forest plot of CRP**

# 5.1.7 IL-6

**Figure S5.1.7 Forest plot of IL-6**

# 5.1.8 IL-1β

**Figure S5.1.8 Forest plot of IL-1β**

# 5.1.9 TNF-α

**Figure S5.1.9 Forest plot of TNF-α**

# 5.1.10 LPS

**Figure S5.1.10 Forest plot of LPS**

# 5.1.11 FFA

**Figure S5.1.11 Forest plot of FFA**

# 5.1.12 Adiponectin

**Figure S5.1.12 Forest plot of adiponectin**

# 5.1.13 Leptin

**Figure S5.1.13 Forest plot of leptin**

# 5.1.14 ALT

**Figure S5.1.14 Forest plot of ALT**

# 5.1.15 AST

**Figure S5.1.15 Forest plot of AST**

# 5.2 Human studies

# 5.2.1 HbA1c

**Figure S5.2.1 Forest plot of HbA1c**

# 5.2.2 Insulin

**Figure S5.2.2 Forest plot of insulin**

# 5.2.3 HOMA-IR

**Figure S5.2.3 Forest plot of HOMA-IR**

# 5.2.4 HOMA-β

**Figure S5.2.4 Forest plot of HOMA-β**

# 5.2.5 CRP

**Figure S5.2.5 Forest plot of	CRP**

# 5.2.6 TNF-α

**Figure S5.2.6 Forest plot of TNF-α**

# 5.2.7 Adiponectin

**Figure S5.2.7 Forest plot of adiponectin**

# 5.2.8 ALT

**Figure S5.2.8 Forest plot of ALT**

# 5.2.9 AST

**Figure S5.2.9 Forest plot of AST**

# Supplementary 6 Results of sensitive analysis for animal and human studies

# 6.1 Animal studies

# 6.1.1 Sensitive analysis of FPG

**Table S6.1.1 Sensitive analysis of FPG**

| ------------------------------------------------------------------------------ |  |  |  |  |  |  |  |  |
| --- | --- | --- | --- | --- | --- | --- | --- | --- |
| Study omitted \| Estimate [95% Conf. Interval] |  |  |  |  |  |  |  |  |
| -------------------+---------------------------------------------------------- |  |  |  |  |  |  |  |  |
| Zhang HY 2021 \| -41.422818 -47.044246 -35.80139 |  |  |  |  |  |  |  |  |
| Zhang HY 2021 \| -41.646239 -47.262976 -36.029502 |  |  |  |  |  |  |  |  |
| Zhang L 2021 \| -41.932088 -47.617755 -36.246422 |  |  |  |  |  |  |  |  |
| Yu RQ 2013 \| -41.070432 -46.709856 -35.431007 |  |  |  |  |  |  |  |  |
| Yu RQ 2013 \| -40.344579 -45.907136 -34.782022 |  |  |  |  |  |  |  |  |
| Yi JD 2021 \| -40.257054 -45.772717 -34.741391 |  |  |  |  |  |  |  |  |
| Chen JJ 2024 \| -39.81834 -45.139467 -34.497214 |  |  |  |  |  |  |  |  |
| Moya-Pérez A 2015\| -39.348065 -44.749512 -33.946618 |  |  |  |  |  |  |  |  |
| Oh YJ 2019 \| -42.924581 -48.228823 -37.620339 |  |  |  |  |  |  |  |  |
| Miao ZH 2022 \| -41.957584 -47.537829 -36.377338 |  |  |  |  |  |  |  |  |
| Miao ZH 2022 \| -41.950527 -47.520142 -36.380912 |  |  |  |  |  |  |  |  |
| Miao ZH 2022 \| -41.751235 -47.35169 -36.15078 |  |  |  |  |  |  |  |  |
| Lee E 2018 \| -41.801173 -47.569875 -36.032471 |  |  |  |  |  |  |  |  |
| Sharma N 2020 \| -41.931692 -48.292961 -35.570423 |  |  |  |  |  |  |  |  |
| Sharma N 2020 \| -42.155039 -48.294895 -36.015183 |  |  |  |  |  |  |  |  |
| Sharma N 2020 \| -41.862813 -48.28569 -35.439936 |  |  |  |  |  |  |  |  |
| Silva GA 2022 \| -42.361256 -47.74962 -36.972892 |  |  |  |  |  |  |  |  |
| Yang XH 2025 \| -39.207386 -44.394135 -34.020637 |  |  |  |  |  |  |  |  |
| Yang XH 2025 \| -38.132887 -43.204312 -33.061462 |  |  |  |  |  |  |  |  |
| Yang XH 2025 \| -40.519712 -45.996998 -35.042425 |  |  |  |  |  |  |  |  |
| Jiang SM 2023 \| -40.923808 -46.520011 -35.327605 |  |  |  |  |  |  |  |  |
| Paola Gauffin Cano 2013\|-40.814844 -46.448433 -35.181254 |  |  |  |  |  |  |  |  |
| -------------------+---------------------------------------------------------- |  |  |  |  |  |  |  |  |
| Combined \| -41.047731 -46.495909 -35.599552 |  |  |  |  |  |  |  |  |
| ------------------------------------------------------------------------------ |  |  |  |  |  |  |  |  |
|  |  |  |  |  |  |  |  |  |

# 6.1.2 Sensitive analysis of HbA1c

**Table S6.1.2 Sensitive analysis of HbA1c**

| ------------------------------------------------------------------------------ |  |  |  |  |  |  |  |  |
| --- | --- | --- | --- | --- | --- | --- | --- | --- |
| Study omitted \| Estimate [95% Conf. Interval] |  |  |  |  |  |  |  |  |
| -------------------+---------------------------------------------------------- |  |  |  |  |  |  |  |  |
| Zhang HY 2021 \| -1.2027815 -1.641618 -.76394499 |  |  |  |  |  |  |  |  |
| Zhang HY 2021 \| -1.3877546 -1.8045989 -.97091036 |  |  |  |  |  |  |  |  |
| Chen JJ 2024 \| -.9928002 -1.3252773 -.66032308 |  |  |  |  |  |  |  |  |
| -------------------+---------------------------------------------------------- |  |  |  |  |  |  |  |  |
| Combined \| -1.169901 -1.503956 -.83584588 |  |  |  |  |  |  |  |  |
| ------------------------------------------------------------------------------ |  |  |  |  |  |  |  |  |

# 6.1.3 Sensitive analysis of BW

**Table S6.1.3 Sensitive analysis of BW**

| ------------------------------------------------------------------------------ |  |  |  |  |  |  |  |  |
| --- | --- | --- | --- | --- | --- | --- | --- | --- |
| Study omitted \| Estimate [95% Conf. Interval] |  |  |  |  |  |  |  |  |
| -------------------+---------------------------------------------------------- |  |  |  |  |  |  |  |  |
| Yang G 2020 \| -13.508204 -19.031564 -7.9848433 |  |  |  |  |  |  |  |  |
| Yang G 2020 \| -13.512252 -19.030869 -7.9936358 |  |  |  |  |  |  |  |  |
| Yang G 2020 \| -13.518433 -19.037485 -7.9993817 |  |  |  |  |  |  |  |  |
| Yang G 2020 \| -13.387657 -18.90642 -7.8688932 |  |  |  |  |  |  |  |  |
| Yang G 2020 \| -13.453222 -18.977522 -7.9289219 |  |  |  |  |  |  |  |  |
| Yang G 2020 \| -13.610265 -19.127995 -8.0925352 |  |  |  |  |  |  |  |  |
| Zhang L 2021 \| -13.601701 -19.132098 -8.0713033 |  |  |  |  |  |  |  |  |
| Yu RQ 2013 \| -13.156354 -18.637185 -7.6755225 |  |  |  |  |  |  |  |  |
| Yu RQ 2013 \| -13.009379 -18.487589 -7.5311693 |  |  |  |  |  |  |  |  |
| Yi JD 2021 \| -13.522523 -19.421549 -7.6234963 |  |  |  |  |  |  |  |  |
| Wu CS 2023 \| -12.861447 -18.332981 -7.3899127 |  |  |  |  |  |  |  |  |
| Wu CS 2023 \| -12.077831 -17.384868 -6.7707943 |  |  |  |  |  |  |  |  |
| Moya-Pérez A 2015\| -13.092716 -18.581983 -7.6034488 |  |  |  |  |  |  |  |  |
| Oh YJ 2019 \| -13.695907 -19.752551 -7.6392635 |  |  |  |  |  |  |  |  |
| Miao ZH 2022 \| -13.419274 -19.032804 -7.805744 |  |  |  |  |  |  |  |  |
| Miao ZH 2022 \| -13.456091 -19.08876 -7.8234211 |  |  |  |  |  |  |  |  |
| Miao ZH 2022 \| -13.413119 -19.040235 -7.7860042 |  |  |  |  |  |  |  |  |
| Huang E 2020 \| -13.664751 -19.301476 -8.0280273 |  |  |  |  |  |  |  |  |
| Huang E 2020 \| -13.605079 -19.246322 -7.9638367 |  |  |  |  |  |  |  |  |
| Huang E 2020 \| -13.706062 -19.28881 -8.1233139 |  |  |  |  |  |  |  |  |
| Cavalcante RGS 2019\| -13.862425 -19.366355 -8.3584955 |  |  |  |  |  |  |  |  |
| Sharma N 2020 \| -11.291252 -15.940051 -6.6424526 |  |  |  |  |  |  |  |  |
| Sharma N 2020 \| -11.438323 -16.189258 -6.6873875 |  |  |  |  |  |  |  |  |
| Sharma N 2020 \| -11.449696 -16.344273 -6.5551183 |  |  |  |  |  |  |  |  |
| Silva GA 2022 \| -14.183111 -19.617548 -8.7486736 |  |  |  |  |  |  |  |  |
| Yang XH 2020 \| -13.82978 -19.508849 -8.1507105 |  |  |  |  |  |  |  |  |
| Yang XH 2020 \| -13.824172 -19.479958 -8.1683852 |  |  |  |  |  |  |  |  |
| Yang XH 2020 \| -13.810569 -19.432403 -8.1887353 |  |  |  |  |  |  |  |  |
| Jiang SM 2023 \| -13.86709 -19.469669 -8.2645117 |  |  |  |  |  |  |  |  |
| -------------------+---------------------------------------------------------- |  |  |  |  |  |  |  |  |
| Combined \| -13.272466 -18.664206 -7.8807247 |  |  |  |  |  |  |  |  |
| ------------------------------------------------------------------------------ |  |  |  |  |  |  |  |  |

# 6.1.4 Sensitive analysis of WC

**Table S6.1.4 Sensitive analysis of WC**

------------------------------------------------------------------------------

Study ommited	|	Coef. [95% Conf. Interval]

-------------------+----------------------------------------------------------

Yu RQ 2013	|	-.78249671	-3.4675357	1.9025422

Yu RQ 2013	|	-.80701714	-3.5311629	1.9171286

Silva GA 2022	|	-2.1664865	-2.4296296	-1.9033433

-------------------+----------------------------------------------------------

Combined	|	-1.2721655	-2.6739951	.12966414

------------------------------------------------------------------------------

# 6.1.5 Sensitive analysis of TC

**Table S6.1.5 Sensitive analysis of TC**

| ------------------------------------------------------------------------------ |  |  |  |  |  |  |  |
| --- | --- | --- | --- | --- | --- | --- | --- |
| Study omitted \| Estimate [95% Conf. Interval] |  |  |  |  |  |  |  |
| -------------------+---------------------------------------------------------- |  |  |  |  |  |  |  |
| Zhang HY 2021 \| -37.402662 -51.016357 -23.788966 |  |  |  |  |  |  |  |
| Zhang HY 2021 \| -37.220287 -50.848483 -23.592092 |  |  |  |  |  |  |  |
| Yang G 2020 \| -35.325668 -48.923083 -21.728252 |  |  |  |  |  |  |  |
| Yang G 2020 \| -36.390461 -49.976928 -22.803993 |  |  |  |  |  |  |  |
| Yang G 2020 \| -36.591296 -50.178858 -23.003734 |  |  |  |  |  |  |  |
| Yang G 2020 \| -35.439569 -49.050749 -21.828389 |  |  |  |  |  |  |  |
| Yang G 2020 \| -37.706083 -51.247308 -24.164857 |  |  |  |  |  |  |  |
| Yang G 2020 \| -42.120929 -55.500322 -28.741537 |  |  |  |  |  |  |  |
| Zhang L 2021 \| -35.515701 -49.139506 -21.891895 |  |  |  |  |  |  |  |
| Yu RQ 2013 \| -37.220819 -50.917295 -23.524343 |  |  |  |  |  |  |  |
| Yu RQ 2013 \| -37.312621 -50.935772 -23.689469 |  |  |  |  |  |  |  |
| Yi JD 2021 \| -35.937276 -49.56909 -22.305463 |  |  |  |  |  |  |  |
| Cano PG 2013 \| -37.057311 -50.804887 -23.309735 |  |  |  |  |  |  |  |
| Moya-Pérez A 2015\| -37.338564 -50.966059 -23.711068 |  |  |  |  |  |  |  |
| Oh YJ 2019 \| -36.867848 -50.684647 -23.051049 |  |  |  |  |  |  |  |
| Lin YK 2022 \| -35.736919 -49.483765 -21.990073 |  |  |  |  |  |  |  |
| Lin YK 2022 \| -34.280625 -47.578361 -20.982889 |  |  |  |  |  |  |  |
| Huang E 2020 \| -36.63225 -50.259043 -23.005456 |  |  |  |  |  |  |  |
| Huang E 2020 \| -36.631431 -50.264452 -22.99841 |  |  |  |  |  |  |  |
| Huang E 2020 \| -36.795989 -50.402365 -23.189612 |  |  |  |  |  |  |  |
| Yang M 2023 \| -36.479509 -50.377255 -22.581763 |  |  |  |  |  |  |  |
| Yang M 2023 \| -36.878771 -50.671668 -23.085874 |  |  |  |  |  |  |  |
| Yang M 2023 \| -36.659421 -50.520666 -22.798177 |  |  |  |  |  |  |  |
| Yang M 2023 \| -36.678214 -50.543268 -22.81316 |  |  |  |  |  |  |  |
| Yang M 2023 \| -35.995635 -49.853965 -22.137305 |  |  |  |  |  |  |  |
| Cavalcante RGS 2019\| -36.271412 -50.011456 -22.531368 |  |  |  |  |  |  |  |
| Sharma N 2020 \| -36.615474 -50.426772 -22.804177 |  |  |  |  |  |  |  |
| Sharma N 2020 \| -36.411369 -50.244722 -22.578016 |  |  |  |  |  |  |  |
| Sharma N 2020 \| -37.479115 -51.068614 -23.889615 |  |  |  |  |  |  |  |
| Silva GA 2022 \| -37.452919 -50.924602 -23.981237 |  |  |  |  |  |  |  |
| Song W 2021 \| -36.600859 -50.268772 -22.932946 |  |  |  |  |  |  |  |
| Song W 2021 \| -36.202973 -49.875079 -22.530867 |  |  |  |  |  |  |  |
| Song W 2021 \| -36.84285 -50.498807 -23.186892 |  |  |  |  |  |  |  |
| Song W 2021 \| -36.485349 -50.15649 -22.814207 |  |  |  |  |  |  |  |
| Song W 2021 \| -37.180115 -50.814577 -23.545653 |  |  |  |  |  |  |  |
| Song W 2021 \| -36.842887 -50.495828 -23.189946 |  |  |  |  |  |  |  |
| Osman A 2021 \| -35.09349 -48.522156 -21.664824 |  |  |  |  |  |  |  |
| Osman A 2021 \| -34.83245 -45.887417 -23.777484 |  |  |  |  |  |  |  |
| Chen JJ 2024 \| -37.356371 -50.974707 -23.738035 |  |  |  |  |  |  |  |
| -------------------+---------------------------------------------------------- |  |  |  |  |  |  |  |
| Combined \| -36.661257 -50.097688 -23.224827 |  |  |  |  |  |  |  |
| ------------------------------------------------------------------------------ |  |  |  |  |  |  |  |

# 6.1.6 Sensitive analysis of TG

**Table S6.1.6 Sensitive analysis of TG**

| ------------------------------------------------------------------------------ |  |  |  |  |  |  |  |  |
| --- | --- | --- | --- | --- | --- | --- | --- | --- |
| Study omitted \| Estimate [95% Conf. Interval] |  |  |  |  |  |  |  |  |
| -------------------+---------------------------------------------------------- |  |  |  |  |  |  |  |  |
| Zhang HY 2021 \| -51.194121 -71.559659 -30.828583 |  |  |  |  |  |  |  |  |
| Zhang HY 2021 \| -51.520282 -71.882188 -31.158376 |  |  |  |  |  |  |  |  |
| Yang G 2020 \| -53.545959 -73.872219 -33.219698 |  |  |  |  |  |  |  |  |
| Yang G 2020 \| -53.122033 -73.423107 -32.820959 |  |  |  |  |  |  |  |  |
| Yang G 2020 \| -54.010654 -69.673122 -38.348187 |  |  |  |  |  |  |  |  |
| Yang G 2020 \| -51.860692 -72.207509 -31.513875 |  |  |  |  |  |  |  |  |
| Yang G 2020 \| -52.754931 -73.093942 -32.415919 |  |  |  |  |  |  |  |  |
| Yang G 2020 \| -51.125285 -71.484917 -30.765653 |  |  |  |  |  |  |  |  |
| Zhang L 2021 \| -51.333996 -71.700342 -30.967649 |  |  |  |  |  |  |  |  |
| Yu RQ 2013 \| -52.4803 -72.94681 -32.01379 |  |  |  |  |  |  |  |  |
| Yu RQ 2013 \| -52.390345 -72.961671 -31.81902 |  |  |  |  |  |  |  |  |
| Yi JD 2021 \| -51.890739 -72.245292 -31.536186 |  |  |  |  |  |  |  |  |
| Chen JJ 2024 \| -52.07693 -72.429986 -31.723875 |  |  |  |  |  |  |  |  |
| Cano PG 2013 \| -51.453529 -71.832562 -31.074496 |  |  |  |  |  |  |  |  |
| Moya-Pérez A 2015\| -50.598115 -70.961872 -30.234358 |  |  |  |  |  |  |  |  |
| Lin YK 2022 \| -50.141785 -70.546539 -29.737031 |  |  |  |  |  |  |  |  |
| Lin YK 2022 \| -48.405048 -68.709131 -28.100965 |  |  |  |  |  |  |  |  |
| Lee E 2018 \| -51.337459 -71.816368 -30.85855 |  |  |  |  |  |  |  |  |
| Huang E 2020 \| -52.076559 -72.47597 -31.677149 |  |  |  |  |  |  |  |  |
| Huang E 2020 \| -52.412653 -72.76105 -32.064256 |  |  |  |  |  |  |  |  |
| Huang E 2020 \| -52.500371 -72.860222 -32.140521 |  |  |  |  |  |  |  |  |
| Yang M 2023 \| -52.329404 -72.73692 -31.921888 |  |  |  |  |  |  |  |  |
| Yang M 2023 \| -52.321316 -72.728466 -31.914165 |  |  |  |  |  |  |  |  |
| Yang M 2023 \| -52.435776 -72.826656 -32.044896 |  |  |  |  |  |  |  |  |
| Yang M 2023 \| -52.191037 -72.617737 -31.764337 |  |  |  |  |  |  |  |  |
| Yang M 2023 \| -51.94755 -72.399929 -31.495172 |  |  |  |  |  |  |  |  |
| Cavalcante RGS 2019\| -51.897465 -72.544617 -31.250313 |  |  |  |  |  |  |  |  |
| Sharma N 2020 \| -46.535855 -66.84079 -26.230919 |  |  |  |  |  |  |  |  |
| Sharma N 2020 \| -47.406501 -67.724037 -27.088964 |  |  |  |  |  |  |  |  |
| Sharma N 2020 \| -46.001744 -66.298686 -25.704802 |  |  |  |  |  |  |  |  |
| Silva GA 2022 \| -51.865314 -72.265631 -31.464997 |  |  |  |  |  |  |  |  |
| Song W 2021 \| -51.475634 -71.865558 -31.08571 |  |  |  |  |  |  |  |  |
| Song W 2021 \| -51.311268 -71.714319 -30.908216 |  |  |  |  |  |  |  |  |
| Song W 2021 \| -51.805281 -72.191329 -31.419232 |  |  |  |  |  |  |  |  |
| Song W 2021 \| -51.757433 -72.137569 -31.377296 |  |  |  |  |  |  |  |  |
| Song W 2021 \| -52.346915 -72.713803 -31.980027 |  |  |  |  |  |  |  |  |
| Song W 2021 \| -51.89841 -72.274873 -31.521946 |  |  |  |  |  |  |  |  |
| Osman A 2021 \| -50.523689 -75.604041 -25.443336 |  |  |  |  |  |  |  |  |
| Osman A 2021 \| -49.403598 -68.791149 -30.016048 |  |  |  |  |  |  |  |  |
| -------------------+---------------------------------------------------------- |  |  |  |  |  |  |  |  |
| Combined \| -51.378586 -71.460252 -31.296919 |  |  |  |  |  |  |  |  |
| ------------------------------------------------------------------------------ |  |  |  |  |  |  |  |  |

# 6.1.7 Sensitive analysis of LDL-C

**Table S6.1.7 Sensitive analysis of LDL-C**

| ------------------------------------------------------------------------------ |  |  |  |  |  |  |  |  |
| --- | --- | --- | --- | --- | --- | --- | --- | --- |
| Study omitted \| Estimate [95% Conf. Interval] |  |  |  |  |  |  |  |  |
| -------------------+---------------------------------------------------------- |  |  |  |  |  |  |  |  |
| Zhang HY 2021 \| -19.166553 -26.951651 -11.381456 |  |  |  |  |  |  |  |  |
| Zhang HY 2021 \| -19.305184 -27.095133 -11.515234 |  |  |  |  |  |  |  |  |
| Yang G 2020 \| -19.634517 -27.376034 -11.893 |  |  |  |  |  |  |  |  |
| Yang G 2020 \| -19.739984 -27.471116 -12.008852 |  |  |  |  |  |  |  |  |
| Yang G 2020 \| -20.289954 -28.005876 -12.574032 |  |  |  |  |  |  |  |  |
| Yang G 2020 \| -19.301532 -27.070246 -11.532818 |  |  |  |  |  |  |  |  |
| Yang G 2020 \| -19.452482 -27.220774 -11.684189 |  |  |  |  |  |  |  |  |
| Yang G 2020 \| -19.325044 -27.089828 -11.56026 |  |  |  |  |  |  |  |  |
| Zhang L 2021 \| -19.0918 -26.910791 -11.272808 |  |  |  |  |  |  |  |  |
| Yu RQ 2013 \| -19.364908 -27.728049 -11.001768 |  |  |  |  |  |  |  |  |
| Yu RQ 2013 \| -19.326867 -27.761196 -10.892537 |  |  |  |  |  |  |  |  |
| Chen JJ 2024 \| -19.145278 -27.323728 -10.966828 |  |  |  |  |  |  |  |  |
| Oh YJ 2019 \| -18.556997 -26.439353 -10.674641 |  |  |  |  |  |  |  |  |
| Lin YK 2022 \| -17.583132 -25.248228 -9.9180352 |  |  |  |  |  |  |  |  |
| Lin YK 2022 \| -15.991515 -23.263474 -8.7195567 |  |  |  |  |  |  |  |  |
| Lee E 2018 \| -19.489443 -27.74235 -11.236536 |  |  |  |  |  |  |  |  |
| Huang E 2020 \| -19.178861 -26.963471 -11.394252 |  |  |  |  |  |  |  |  |
| Huang E 2020 \| -19.179413 -26.97341 -11.385417 |  |  |  |  |  |  |  |  |
| Huang E 2020 \| -19.262581 -27.028245 -11.496916 |  |  |  |  |  |  |  |  |
| Cavalcante RGS 2019\| -17.637866 -25.340204 -9.9355272 |  |  |  |  |  |  |  |  |
| Sharma N 2020 \| -18.876639 -26.72717 -11.026109 |  |  |  |  |  |  |  |  |
| Sharma N 2020 \| -18.940104 -26.779323 -11.100884 |  |  |  |  |  |  |  |  |
| Sharma N 2020 \| -18.775311 -26.631945 -10.918677 |  |  |  |  |  |  |  |  |
| Silva GA 2022 \| -19.143856 -26.977677 -11.310035 |  |  |  |  |  |  |  |  |
| Song W 2021 \| -19.241212 -27.020774 -11.46165 |  |  |  |  |  |  |  |  |
| Song W 2021 \| -19.177169 -26.947591 -11.406748 |  |  |  |  |  |  |  |  |
| Song W 2021 \| -18.803124 -26.592171 -11.014077 |  |  |  |  |  |  |  |  |
| Song W 2021 \| -18.650513 -26.463205 -10.837821 |  |  |  |  |  |  |  |  |
| Song W 2021 \| -19.458376 -27.28505 -11.631701 |  |  |  |  |  |  |  |  |
| Song W 2021 \| -19.318974 -27.14866 -11.489288 |  |  |  |  |  |  |  |  |
| Osman A 2021 \| -16.9194 -23.868415 -9.9703841 |  |  |  |  |  |  |  |  |
| Osman A 2021 \| -16.110857 -22.129162 -10.092552 |  |  |  |  |  |  |  |  |
| -------------------+---------------------------------------------------------- |  |  |  |  |  |  |  |  |
| Combined \| -18.857759 -26.480285 -11.235233 |  |  |  |  |  |  |  |  |
| ------------------------------------------------------------------------------ |  |  |  |  |  |  |  |  |

# 6.1.8 Sensitive analysis of HDL-C

**Table S6.1.8 Sensitive analysis of HDL-C**

| ------------------------------------------------------------------------------ |  |  |  |  |  |  |  |  |
| --- | --- | --- | --- | --- | --- | --- | --- | --- |
| Study omitted \| Estimate [95% Conf. Interval] |  |  |  |  |  |  |  |  |
| -------------------+---------------------------------------------------------- |  |  |  |  |  |  |  |  |
| Zhang HY 2021 \| 10.231852 7.2913938 13.172311 |  |  |  |  |  |  |  |  |
| Zhang HY 2021 \| 9.9881298 7.0377187 12.938541 |  |  |  |  |  |  |  |  |
| Yang G 2020 \| 10.051994 7.1520579 12.95193 |  |  |  |  |  |  |  |  |
| Yang G 2020 \| 9.9335612 7.0352722 12.83185 |  |  |  |  |  |  |  |  |
| Yang G 2020 \| 9.8728751 6.9749069 12.770843 |  |  |  |  |  |  |  |  |
| Yang G 2020 \| 9.854064 6.9609895 12.747139 |  |  |  |  |  |  |  |  |
| Yang G 2020 \| 9.9531301 7.0551634 12.851097 |  |  |  |  |  |  |  |  |
| Yang G 2020 \| 10.004442 7.1046705 12.904214 |  |  |  |  |  |  |  |  |
| Zhang L 2021 \| 9.7845396 6.8997118 12.669367 |  |  |  |  |  |  |  |  |
| Yu RQ 2013 \| 10.48007 7.4232029 13.536937 |  |  |  |  |  |  |  |  |
| Yu RQ 2013 \| 10.483309 7.429139 13.537479 |  |  |  |  |  |  |  |  |
| Chen JJ 2024 \| 10.350332 7.3114347 13.38923 |  |  |  |  |  |  |  |  |
| Oh YJ 2019 \| 10.403085 7.4288097 13.37736 |  |  |  |  |  |  |  |  |
| Lin YK 2022 \| 10.18895 7.1287867 13.249113 |  |  |  |  |  |  |  |  |
| Lin YK 2022 \| 9.6576613 6.7217368 12.593586 |  |  |  |  |  |  |  |  |
| Lee E 2018 \| 10.723398 7.9159193 13.530876 |  |  |  |  |  |  |  |  |
| Cavalcante RGS 2019\| 10.46405 7.4689038 13.459195 |  |  |  |  |  |  |  |  |
| Sharma N 2020 \| 10.971213 8.0327064 13.909721 |  |  |  |  |  |  |  |  |
| Sharma N 2020 \| 10.355609 7.4067859 13.304432 |  |  |  |  |  |  |  |  |
| Sharma N 2020 \| 9.9046085 6.938718 12.870499 |  |  |  |  |  |  |  |  |
| Silva GA 2022 \| 10.482368 7.547022 13.417715 |  |  |  |  |  |  |  |  |
| Song W 2021 \| 9.5693471 6.6158624 12.522832 |  |  |  |  |  |  |  |  |
| Song W 2021 \| 9.2769327 6.3411227 12.212743 |  |  |  |  |  |  |  |  |
| Song W 2021 \| 9.2928323 6.3562552 12.229409 |  |  |  |  |  |  |  |  |
| Song W 2021 \| 9.0226719 6.1010327 11.944311 |  |  |  |  |  |  |  |  |
| Song W 2021 \| 9.91408 6.9357312 12.892429 |  |  |  |  |  |  |  |  |
| Song W 2021 \| 9.7896019 6.8342389 12.744965 |  |  |  |  |  |  |  |  |
| Osman A 2021 \| 10.399813 7.0604151 13.739212 |  |  |  |  |  |  |  |  |
| Osman A 2021 \| 9.4327011 6.847208 12.018194 |  |  |  |  |  |  |  |  |
| -------------------+---------------------------------------------------------- |  |  |  |  |  |  |  |  |
| Combined \| 10.029123 7.1317959 12.92645 |  |  |  |  |  |  |  |  |
| ------------------------------------------------------------------------------ |  |  |  |  |  |  |  |  |

# 6.1.9 Sensitive analysis of insulin

**Table S6.1.9 Sensitive analysis of insulin**

| ------------------------------------------------------------------------------ |  |  |  |  |  |  |  |  |
| --- | --- | --- | --- | --- | --- | --- | --- | --- |
| Study omitted \| Estimate [95% Conf. Interval] |  |  |  |  |  |  |  |  |
| -------------------+---------------------------------------------------------- |  |  |  |  |  |  |  |  |
| Zhang HY 2021 \| -3.2880733 -3.5933069 -2.9828398 |  |  |  |  |  |  |  |  |
| Zhang HY 2021 \| -3.0282247 -3.3300479 -2.7264014 |  |  |  |  |  |  |  |  |
| Zhang L 2021 \| -3.8688498 -4.2254174 -3.5122822 |  |  |  |  |  |  |  |  |
| Cano PG 2013 \| -2.8634156 -3.157499 -2.5693321 |  |  |  |  |  |  |  |  |
| Moya-Pérez A 2015\| -3.3068696 -3.6084557 -3.0052834 |  |  |  |  |  |  |  |  |
| Oh YJ 2019 \| -3.4170008 -3.7250044 -3.1089972 |  |  |  |  |  |  |  |  |
| Sharma N 2020 \| -3.8976273 -4.2679671 -3.5272875 |  |  |  |  |  |  |  |  |
| Sharma N 2020 \| -3.8976273 -4.2679671 -3.5272875 |  |  |  |  |  |  |  |  |
| Sharma N 2020 \| -3.9833299 -4.3802204 -3.5864395 |  |  |  |  |  |  |  |  |
| Silva GA 2022 \| -3.6621641 -3.9791559 -3.3451723 |  |  |  |  |  |  |  |  |
| Yu RQ 2013 \| -3.1435388 -3.4469418 -2.8401358 |  |  |  |  |  |  |  |  |
| Yu RQ 2013 \| -2.8837206 -3.1756753 -2.591766 |  |  |  |  |  |  |  |  |
| Yi JD 2021 \| -3.4252972 -3.7382162 -3.1123782 |  |  |  |  |  |  |  |  |
| Chen JJ 2024 \| -2.4266435 -2.6760757 -2.1772113 |  |  |  |  |  |  |  |  |
| Lee E 2018 \| -3.1574867 -3.4602645 -2.854709 |  |  |  |  |  |  |  |  |
| -------------------+---------------------------------------------------------- |  |  |  |  |  |  |  |  |
| Combined \| -3.3332616 -3.6355265 -3.0309967 |  |  |  |  |  |  |  |  |
| ------------------------------------------------------------------------------ |  |  |  |  |  |  |  |  |

# 6.1.10 Sensitive analysis of HOMA-IR

**Table S6.1.10 Sensitive analysis of HOMA-IR**

| ------------------------------------------------------------------------------ |  |  |  |  |  |  |  |  |
| --- | --- | --- | --- | --- | --- | --- | --- | --- |
| Study omitted \| Estimate [95% Conf. Interval] |  |  |  |  |  |  |  |  |
| -------------------+---------------------------------------------------------- |  |  |  |  |  |  |  |  |
| Zhang HY 2021 \| -2.9179907 -5.7693016 -.06667969 |  |  |  |  |  |  |  |  |
| Zhang HY 2021 \| -2.7444465 -5.6363676 .1474747 |  |  |  |  |  |  |  |  |
| Zhang L 2021 \| -3.3441448 -6.7581949 .06990537 |  |  |  |  |  |  |  |  |
| Yi JD 2021 \| -3.1184833 -6.1245244 -.11244214 |  |  |  |  |  |  |  |  |
| Chen JJ 2024 \| -1.6346601 -2.4889528 -.78036726 |  |  |  |  |  |  |  |  |
| Oh YJ 2019 \| -3.4392646 -6.7362507 -.14227844 |  |  |  |  |  |  |  |  |
| Silva GA 2022 \| -3.5494954 -6.6713785 -.42761243 |  |  |  |  |  |  |  |  |
| -------------------+---------------------------------------------------------- |  |  |  |  |  |  |  |  |
| Combined \| -3.0227712 -5.6887148 -.35682759 |  |  |  |  |  |  |  |  |
| ------------------------------------------------------------------------------ |  |  |  |  |  |  |  |  |

# 6.1.11 Sensitive analysis of OGTT 0h blood glucose

**Table S6.1.11 Sensitive analysis of OGTT 0h blood glucose**

| ------------------------------------------------------------------------------ |  |  |  |  |  |  |  |  |
| --- | --- | --- | --- | --- | --- | --- | --- | --- |
| Study ommited \| Coef. [95% Conf. Interval] |  |  |  |  |  |  |  |  |
| -------------------+---------------------------------------------------------- |  |  |  |  |  |  |  |  |
| Miao ZH 2022 \| -1.3710139 -1.7004833 -1.0415444 |  |  |  |  |  |  |  |  |
| Miao ZH 2022 \| -1.2663999 -1.6753667 -.85743316 |  |  |  |  |  |  |  |  |
| Miao ZH 2022 \| -1.4727471 -1.8227645 -1.1227298 |  |  |  |  |  |  |  |  |
| Zhang HY 2021 \| -1.2658634 -1.629957 -.90176988 |  |  |  |  |  |  |  |  |
| Zhang HY 2021 \| -1.2921332 -1.6632931 -.92097342 |  |  |  |  |  |  |  |  |
| -------------------+---------------------------------------------------------- |  |  |  |  |  |  |  |  |
| Combined \| -1.3410918 -1.6538252 -1.0283584 |  |  |  |  |  |  |  |  |
| ------------------------------------------------------------------------------ |  |  |  |  |  |  |  |  |

# 6.1.12 Sensitive analysis of OGTT 2h blood glucose

**Table S6.1.12 Sensitive analysis of OGTT 2h blood glucose**

| ------------------------------------------------------------------------------ |  |  |  |  |  |  |  |  |
| --- | --- | --- | --- | --- | --- | --- | --- | --- |
| Study ommited \| Coef. [95% Conf. Interval] |  |  |  |  |  |  |  |  |
| -------------------+---------------------------------------------------------- |  |  |  |  |  |  |  |  |
| Zhang HY 2021 \| -2.4470699 -3.8245689 -1.0695709 |  |  |  |  |  |  |  |  |
| Zhang HY 2021 \| -2.1879996 -3.686571 -.68942827 |  |  |  |  |  |  |  |  |
| Miao ZH 2022 \| -2.7193721 -3.7727225 -1.6660217 |  |  |  |  |  |  |  |  |
| Miao ZH 2022 \| -1.9597384 -2.772897 -1.1465798 |  |  |  |  |  |  |  |  |
| Miao ZH 2022 \| -2.3014537 -3.7146323 -.88827515 |  |  |  |  |  |  |  |  |
| -------------------+---------------------------------------------------------- |  |  |  |  |  |  |  |  |
| Combined \| -2.3322099 -3.4547501 -1.2096696 |  |  |  |  |  |  |  |  |
| ------------------------------------------------------------------------------ |  |  |  |  |  |  |  |  |

# 6.1.13 Sensitive analysis of CRP

**Table S6.1.13 Sensitive analysis of CRP**

| ------------------------------------------------------------------------------ |  |  |  |  |  |  |  |  |
| --- | --- | --- | --- | --- | --- | --- | --- | --- |
| Study ommited \| Coef. [95% Conf. Interval] |  |  |  |  |  |  |  |  |
| -------------------+---------------------------------------------------------- |  |  |  |  |  |  |  |  |
| Zhang L 2021 \| -36.479334 -59.212719 -13.745948 |  |  |  |  |  |  |  |  |
| Yang G 2020 \| -36.465234 -58.543168 -14.3873 |  |  |  |  |  |  |  |  |
| Yang G 2020 \| -32.574434 -53.180925 -11.967944 |  |  |  |  |  |  |  |  |
| Yang G 2020 \| -31.748575 -52.704255 -10.792895 |  |  |  |  |  |  |  |  |
| Yang G 2020 \| -28.456106 -45.199383 -11.71283 |  |  |  |  |  |  |  |  |
| Yang G 2020 \| -39.42128 -57.889519 -20.953041 |  |  |  |  |  |  |  |  |
| Yang G 2020 \| -30.213917 -50.492164 -9.9356706 |  |  |  |  |  |  |  |  |
| -------------------+---------------------------------------------------------- |  |  |  |  |  |  |  |  |
| Combined \| -33.62774 -52.449531 -14.805949 |  |  |  |  |  |  |  |  |
| ------------------------------------------------------------------------------ |  |  |  |  |  |  |  |  |
|  |  |  |  |  |  |  |  |  |

# 6.1.14 Sensitive analysis of IL-6

**Table S6.1.14 Sensitive analysis of IL-6**

| ------------------------------------------------------------------------------ |  |  |  |  |  |  |  |  |
| --- | --- | --- | --- | --- | --- | --- | --- | --- |
| Study omitted \| Estimate [95% Conf. Interval] |  |  |  |  |  |  |  |  |
| -------------------+---------------------------------------------------------- |  |  |  |  |  |  |  |  |
| Zhang L 2021 \| -15.515621 -18.939952 -12.09129 |  |  |  |  |  |  |  |  |
| Moya-Pérez A 2015\| -15.384779 -18.501404 -12.268154 |  |  |  |  |  |  |  |  |
| Yang G 2020 \| -14.268005 -17.29072 -11.24529 |  |  |  |  |  |  |  |  |
| Yang G 2020 \| -15.187072 -18.617352 -11.756791 |  |  |  |  |  |  |  |  |
| Yang G 2020 \| -15.257654 -18.744813 -11.770496 |  |  |  |  |  |  |  |  |
| Yang G 2020 \| -14.68195 -18.209346 -11.154553 |  |  |  |  |  |  |  |  |
| Yang G 2020 \| -15.184446 -18.451178 -11.917713 |  |  |  |  |  |  |  |  |
| Yang G 2020 \| -15.40605 -18.815899 -11.996202 |  |  |  |  |  |  |  |  |
| Sun RY 2022 \| -15.81988 -18.867678 -12.772083 |  |  |  |  |  |  |  |  |
| Sun RY 2022 \| -14.617401 -18.205762 -11.02904 |  |  |  |  |  |  |  |  |
| Sun RY 2022 \| -14.308018 -17.463648 -11.152387 |  |  |  |  |  |  |  |  |
| -------------------+---------------------------------------------------------- |  |  |  |  |  |  |  |  |
| Combined \| -15.069375 -18.231301 -11.907449 |  |  |  |  |  |  |  |  |
| ------------------------------------------------------------------------------ |  |  |  |  |  |  |  |  |

# 6.1.15 Sensitive analysis of IL-1β

**Table S6.1.15 Sensitive analysis of IL-1β**

| ------------------------------------------------------------------------------ |  |  |  |  |  |  |  |  |
| --- | --- | --- | --- | --- | --- | --- | --- | --- |
| Study ommited \| Coef. [95% Conf. Interval] |  |  |  |  |  |  |  |  |
| -------------------+---------------------------------------------------------- |  |  |  |  |  |  |  |  |
| Zhang L 2021 \| -4.2869968 -6.3138739 -2.2601197 |  |  |  |  |  |  |  |  |
| Moya-Pérez A 2015\| -5.1090161 -7.2730758 -2.9449565 |  |  |  |  |  |  |  |  |
| Yang G 2020 \| -4.9668603 -7.1928463 -2.7408742 |  |  |  |  |  |  |  |  |
| Yang G 2020 \| -5.4338685 -7.7252387 -3.1424984 |  |  |  |  |  |  |  |  |
| Yang G 2020 \| -4.286516 -6.3931803 -2.1798518 |  |  |  |  |  |  |  |  |
| Yang G 2020 \| -4.5215674 -6.6258118 -2.4173229 |  |  |  |  |  |  |  |  |
| Yang G 2020 \| -5.7931172 -8.0259842 -3.5602501 |  |  |  |  |  |  |  |  |
| Yang G 2020 \| -4.6888662 -6.804588 -2.5731443 |  |  |  |  |  |  |  |  |
| Oh YJ 2019 \| -5.7243493 -8.23629 -3.2124086 |  |  |  |  |  |  |  |  |
| Sun RY 2022 \| -5.5632926 -8.1635889 -2.9629964 |  |  |  |  |  |  |  |  |
| Sun RY 2022 \| -5.4838915 -7.9507422 -3.0170408 |  |  |  |  |  |  |  |  |
| Sun RY 2022 \| -5.4582282 -8.046352 -2.8701044 |  |  |  |  |  |  |  |  |
| -------------------+---------------------------------------------------------- |  |  |  |  |  |  |  |  |
| Combined \| -5.1086739 -7.2696993 -2.9476485 |  |  |  |  |  |  |  |  |
| ------------------------------------------------------------------------------ |  |  |  |  |  |  |  |  |

# 6.1.16 Sensitive analysis of TNF-α

**Table S6.1.16 Sensitive analysis of TNF-α**

| ------------------------------------------------------------------------------ |  |  |  |  |  |  |  |  |
| --- | --- | --- | --- | --- | --- | --- | --- | --- |
| Study omitted \| Estimate [95% Conf. Interval] |  |  |  |  |  |  |  |  |
| -------------------+---------------------------------------------------------- |  |  |  |  |  |  |  |  |
| Zhang L 2021 \| -24.038678 -35.069059 -13.008298 |  |  |  |  |  |  |  |  |
| Moya-Pérez A 2015\| -27.980236 -41.433666 -14.526806 |  |  |  |  |  |  |  |  |
| Yang G 2020 \| -28.784596 -42.388434 -15.180758 |  |  |  |  |  |  |  |  |
| Yang G 2020 \| -29.138076 -42.788208 -15.487943 |  |  |  |  |  |  |  |  |
| Yang G 2020 \| -33.217114 -47.245757 -19.188471 |  |  |  |  |  |  |  |  |
| Yang G 2020 \| -28.164448 -41.650844 -14.678052 |  |  |  |  |  |  |  |  |
| Yang G 2020 \| -32.012964 -45.814565 -18.211362 |  |  |  |  |  |  |  |  |
| Yang G 2020 \| -34.437762 -48.476723 -20.398801 |  |  |  |  |  |  |  |  |
| Sun RY 2022 \| -34.798967 -50.752688 -18.845246 |  |  |  |  |  |  |  |  |
| Sun RY 2022 \| -34.298113 -50.574621 -18.021604 |  |  |  |  |  |  |  |  |
| Sun RY 2022 \| -33.597681 -48.775789 -18.419573 |  |  |  |  |  |  |  |  |
| Oh YJ 2019 \| -35.865561 -51.388863 -20.34226 |  |  |  |  |  |  |  |  |
| -------------------+---------------------------------------------------------- |  |  |  |  |  |  |  |  |
| Combined \| -31.421204 -44.861322 -17.981087 |  |  |  |  |  |  |  |  |
| ------------------------------------------------------------------------------ |  |  |  |  |  |  |  |  |

# 6.1.17 Sensitive analysis of LPS

**Table S6.1.17 Sensitive analysis of LPS**

| ------------------------------------------------------------------------------ |  |  |  |  |  |  |  |  |
| --- | --- | --- | --- | --- | --- | --- | --- | --- |
| Study omitted \| Estimate [95% Conf. Interval] |  |  |  |  |  |  |  |  |
| -------------------+---------------------------------------------------------- |  |  |  |  |  |  |  |  |
| Zhang L 2021 \| -62.682677 -90.401421 -34.963933 |  |  |  |  |  |  |  |  |
| Yang G 2020 \| -61.86781 -93.745892 -29.989727 |  |  |  |  |  |  |  |  |
| Yang G 2020 \| -59.774424 -93.299418 -26.249431 |  |  |  |  |  |  |  |  |
| Yang G 2020 \| -56.120432 -90.521817 -21.719047 |  |  |  |  |  |  |  |  |
| Yang G 2020 \| -45.07035 -69.694232 -20.446468 |  |  |  |  |  |  |  |  |
| Yang G 2020 \| -49.311144 -77.412806 -21.209481 |  |  |  |  |  |  |  |  |
| Yang G 2020 \| -51.420411 -82.398156 -20.442667 |  |  |  |  |  |  |  |  |
| -------------------+---------------------------------------------------------- |  |  |  |  |  |  |  |  |
| Combined \| -55.231144 -83.553812 -26.908476 |  |  |  |  |  |  |  |  |
| ------------------------------------------------------------------------------ |  |  |  |  |  |  |  |  |

# 6.1.18 Sensitive analysis of FFA

**6.1.18 Sensitive analysis of FFA**

| ------------------------------------------------------------------------------ |  |  |  |  |  |  |  |  |
| --- | --- | --- | --- | --- | --- | --- | --- | --- |
| Study omitted \| Estimate [95% Conf. Interval] |  |  |  |  |  |  |  |  |
| -------------------+---------------------------------------------------------- |  |  |  |  |  |  |  |  |
| Zhang L 2021 \| -118.24536 -173.99455 -62.496165 |  |  |  |  |  |  |  |  |
| Chen JJ 2024 \| -89.589507 -151.93349 -27.24552 |  |  |  |  |  |  |  |  |
| Yang G 2020 \| -138.88034 -205.93876 -71.821925 |  |  |  |  |  |  |  |  |
| Yang G 2020 \| -142.24239 -211.38808 -73.09671 |  |  |  |  |  |  |  |  |
| Yang G 2020 \| -125.58558 -188.91681 -62.254352 |  |  |  |  |  |  |  |  |
| Yang G 2020 \| -121.39007 -181.84223 -60.937906 |  |  |  |  |  |  |  |  |
| Yang G 2020 \| -151.38114 -221.54057 -81.2217 |  |  |  |  |  |  |  |  |
| Yang G 2020 \| -138.61519 -206.95897 -70.271412 |  |  |  |  |  |  |  |  |
| Silva GA 2022 \| -141.84857 -197.51232 -86.184818 |  |  |  |  |  |  |  |  |
| -------------------+---------------------------------------------------------- |  |  |  |  |  |  |  |  |
| Combined \| -130.36317 -191.51858 -69.207768 |  |  |  |  |  |  |  |  |
| ------------------------------------------------------------------------------ |  |  |  |  |  |  |  |  |

# 6.1.19 Sensitive analysis of Leptin

**Table S6.1.19 Sensitive analysis of Leptin**

| ------------------------------------------------------------------------------ |  |  |  |  |  |  |  |  |
| --- | --- | --- | --- | --- | --- | --- | --- | --- |
| Study omitted \| Estimate [95% Conf. Interval] |  |  |  |  |  |  |  |  |
| -------------------+---------------------------------------------------------- |  |  |  |  |  |  |  |  |
| Moya-Pérez A 2015\| -322.18109 -629.43078 -14.931399 |  |  |  |  |  |  |  |  |
| Cano PG 2013 \| -322.33561 -629.11987 -15.55136 |  |  |  |  |  |  |  |  |
| Huang E 2020 \| -10.535894 -23.779615 2.7078267 |  |  |  |  |  |  |  |  |
| Huang E 2020 \| -13.299398 -28.920328 2.3215317 |  |  |  |  |  |  |  |  |
| Huang E 2020 \| -15.805671 -33.257657 1.6463147 |  |  |  |  |  |  |  |  |
| -------------------+---------------------------------------------------------- |  |  |  |  |  |  |  |  |
| Combined \| -20.953503 -39.794593 -2.1124137 |  |  |  |  |  |  |  |  |
| ------------------------------------------------------------------------------ |  |  |  |  |  |  |  |  |

# 6.1.20 Sensitive analysis of ALT

**Table S6.1.20 Sensitive analysis of ALT**

| ------------------------------------------------------------------------------ |  |  |  |  |  |  |  |  |
| --- | --- | --- | --- | --- | --- | --- | --- | --- |
| Study omitted \| Estimate [95% Conf. Interval] |  |  |  |  |  |  |  |  |
| -------------------+---------------------------------------------------------- |  |  |  |  |  |  |  |  |
| Moya-Pérez A 2015\| -10.731904 -14.159869 -7.3039403 |  |  |  |  |  |  |  |  |
| Lin YK 2022 \| -10.209109 -15.480577 -4.9376402 |  |  |  |  |  |  |  |  |
| Lin YK 2022 \| -10.639617 -15.92143 -5.3578026 |  |  |  |  |  |  |  |  |
| Yang M 2023 \| -9.7497121 -15.145558 -4.3538667 |  |  |  |  |  |  |  |  |
| Yang M 2023 \| -9.9009346 -15.392492 -4.4093768 |  |  |  |  |  |  |  |  |
| Yang M 2023 \| -9.8404477 -15.295529 -4.3853666 |  |  |  |  |  |  |  |  |
| Yang M 2023 \| -9.7799568 -15.198881 -4.3610324 |  |  |  |  |  |  |  |  |
| Yang M 2023 \| -9.467523 -14.659762 -4.2752836 |  |  |  |  |  |  |  |  |
| Sharma N 2020 \| -9.1849122 -14.318636 -4.0511879 |  |  |  |  |  |  |  |  |
| Sharma N 2020 \| -9.2651598 -14.442437 -4.087883 |  |  |  |  |  |  |  |  |
| Sharma N 2020 \| -8.1539908 -12.452661 -3.8553207 |  |  |  |  |  |  |  |  |
| -------------------+---------------------------------------------------------- |  |  |  |  |  |  |  |  |
| Combined \| -9.7179546 -14.690089 -4.7458205 |  |  |  |  |  |  |  |  |
| ------------------------------------------------------------------------------ |  |  |  |  |  |  |  |  |

# 6.1.21 Sensitive analysis of AST

**Table S6.1.21 Sensitive analysis of AST**

| ------------------------------------------------------------------------------ |  |  |  |  |  |  |  |  |
| --- | --- | --- | --- | --- | --- | --- | --- | --- |
| Study omitted \| Estimate [95% Conf. Interval] |  |  |  |  |  |  |  |  |
| -------------------+---------------------------------------------------------- |  |  |  |  |  |  |  |  |
| Lin YK 2022 \| -27.431786 -35.719609 -19.143963 |  |  |  |  |  |  |  |  |
| Lin YK 2022 \| -27.94955 -35.069844 -20.829257 |  |  |  |  |  |  |  |  |
| Yang M 2023 \| -25.090699 -34.332578 -15.848819 |  |  |  |  |  |  |  |  |
| Yang M 2023 \| -24.898352 -34.066737 -15.729966 |  |  |  |  |  |  |  |  |
| Yang M 2023 \| -25.625641 -35.171833 -16.079448 |  |  |  |  |  |  |  |  |
| Yang M 2023 \| -25.321342 -34.806293 -15.836391 |  |  |  |  |  |  |  |  |
| Yang M 2023 \| -24.497934 -33.441073 -15.554795 |  |  |  |  |  |  |  |  |
| Sharma N 2020 \| -24.582672 -33.426327 -15.739017 |  |  |  |  |  |  |  |  |
| Sharma N 2020 \| -22.960562 -31.335118 -14.586006 |  |  |  |  |  |  |  |  |
| Sharma N 2020 \| -23.340339 -31.870141 -14.810538 |  |  |  |  |  |  |  |  |
| -------------------+---------------------------------------------------------- |  |  |  |  |  |  |  |  |
| Combined \| -25.169455 -33.494777 -16.844132 |  |  |  |  |  |  |  |  |
| ------------------------------------------------------------------------------ |  |  |  |  |  |  |  |  |

# 6.2 Human studies

# 6.2.1 Sensitive analysis of FPG

**Table S6.2.1 Sensitive analysis of FPG**

| ------------------------------------------------------------------------------ |  |  |  |  |  |  |  |  |
| --- | --- | --- | --- | --- | --- | --- | --- | --- |
| Study ommited \| Coef. [95% Conf. Interval] |  |  |  |  |  |  |  |  |
| -------------------+---------------------------------------------------------- |  |  |  |  |  |  |  |  |
| Yang JL 2023 \| -9.7869514 -20.061457 .48755384 |  |  |  |  |  |  |  |  |
| Luangphiphat W 2025\| -9.9699066 -20.244166 .30435301 |  |  |  |  |  |  |  |  |
| Rabiei S 2018 \| -6.501333 -17.027483 4.0248171 |  |  |  |  |  |  |  |  |
| Eslamparast T 2014\| -9.2318625 -19.454353 .99062825 |  |  |  |  |  |  |  |  |
| Parastouei K 2020 \| -6.2858821 -14.696263 2.1244987 |  |  |  |  |  |  |  |  |
| Rahimi F 2022 \| -9.0246358 -19.637326 1.5880548 |  |  |  |  |  |  |  |  |
| Gorenjak M 2015 \| -9.2935373 -19.00868 .42160589 |  |  |  |  |  |  |  |  |
| Cicero AFG 2020 \| -9.7381853 -18.629095 -.84727532 |  |  |  |  |  |  |  |  |
| Wastyk HC 2023 \| -9.106882 -19.340058 1.1262943 |  |  |  |  |  |  |  |  |
| -------------------+---------------------------------------------------------- |  |  |  |  |  |  |  |  |
| Combined \| -8.7571432 -18.288264 .77397788 |  |  |  |  |  |  |  |  |
| ------------------------------------------------------------------------------ |  |  |  |  |  |  |  |  |

# 6.2.2 Sensitive analysis of HbA1c

**Table S6.2.2 Sensitive analysis of HbA1c**

| ------------------------------------------------------------------------------ |  |  |  |  |  |  |  |  |
| --- | --- | --- | --- | --- | --- | --- | --- | --- |
| Study omitted \| Estimate [95% Conf. Interval] |  |  |  |  |  |  |  |  |
| -------------------+---------------------------------------------------------- |  |  |  |  |  |  |  |  |
| Luangphiphat W 2025\| -.06330728 -.16504285 .03842829 |  |  |  |  |  |  |  |  |
| Yang JL 2023 \| -.09 -.17175944 -.00824056 |  |  |  |  |  |  |  |  |
| Depommier C 2020 \| -.07348285 -.15351122 .00654551 |  |  |  |  |  |  |  |  |
| -------------------+---------------------------------------------------------- |  |  |  |  |  |  |  |  |
| Combined \| -.07718056 -.1476841 -.00667703 |  |  |  |  |  |  |  |  |
| ------------------------------------------------------------------------------ |  |  |  |  |  |  |  |  |
|  |  |  |  |  |  |  |  |  |

# 6.2.3 Sensitive analysis of BW

**Table S6.2.3 Sensitive analysis of BW**

| ------------------------------------------------------------------------------ |  |  |  |  |  |  |  |  |
| --- | --- | --- | --- | --- | --- | --- | --- | --- |
| Study omitted \| Estimate [95% Conf. Interval] |  |  |  |  |  |  |  |  |
| -------------------+---------------------------------------------------------- |  |  |  |  |  |  |  |  |
| Luangphiphat W 2025\| -1.0654029 -1.8320406 -.29876514 |  |  |  |  |  |  |  |  |
| Rahimi F 2022 \| -1.1571722 -1.8447202 -.46962425 |  |  |  |  |  |  |  |  |
| Gorenjak M 2015 \| -1.0752735 -1.8389857 -.31156119 |  |  |  |  |  |  |  |  |
| Rabiei S 2018 \| -.79879286 -1.0773922 -.52019356 |  |  |  |  |  |  |  |  |
| Depommier C 2020 \| -1.5901288 -2.405711 -.77454667 |  |  |  |  |  |  |  |  |
| -------------------+---------------------------------------------------------- |  |  |  |  |  |  |  |  |
| Combined \| -1.0614747 -1.7025904 -.42035905 |  |  |  |  |  |  |  |  |
| ------------------------------------------------------------------------------ |  |  |  |  |  |  |  |  |

# 6.2.4 Sensitive analysis of WC

**Table S6.2.4 Sensitive analysis of WC**

| ------------------------------------------------------------------------------ |  |  |  |  |  |  |  |  |
| --- | --- | --- | --- | --- | --- | --- | --- | --- |
| Study omitted \| Estimate [95% Conf. Interval] |  |  |  |  |  |  |  |  |
| -------------------+---------------------------------------------------------- |  |  |  |  |  |  |  |  |
| Luangphiphat W 2025\| -2.1386627 -3.8240773 -.45324822 |  |  |  |  |  |  |  |  |
| Parastouei K 2020 \| -1.6205699 -2.6944906 -.54664924 |  |  |  |  |  |  |  |  |
| Rahimi F 2022 \| -2.3684705 -4.0254748 -.71146623 |  |  |  |  |  |  |  |  |
| Yang JL 2023 \| -2.4410813 -3.9892351 -.89292757 |  |  |  |  |  |  |  |  |
| Gorenjak M 2015 \| -2.0423293 -3.6853013 -.39935729 |  |  |  |  |  |  |  |  |
| Rabiei S 2015 \| -1.8458219 -3.5330741 -.15856963 |  |  |  |  |  |  |  |  |
| Cicero AFG 2020 \| -2.0006492 -3.817998 -.18330044 |  |  |  |  |  |  |  |  |
| Wastyk HC 2023 \| -2.2456806 -3.8972195 -.59414177 |  |  |  |  |  |  |  |  |
| Depommier C 2020 \| -2.2910741 -4.0044248 -.57772333 |  |  |  |  |  |  |  |  |
| -------------------+---------------------------------------------------------- |  |  |  |  |  |  |  |  |
| Combined \| -2.124067 -3.6978874 -.55024662 |  |  |  |  |  |  |  |  |
| ------------------------------------------------------------------------------ |  |  |  |  |  |  |  |  |

# 6.2.5 Sensitive analysis of TC

**Table S6.2.5 Sensitive analysis of TC**

| ------------------------------------------------------------------------------ |  |  |  |  |  |  |  |
| --- | --- | --- | --- | --- | --- | --- | --- |
| Study omitted \| Estimate [95% Conf. Interval] |  |  |  |  |  |  |  |
| -------------------+---------------------------------------------------------- |  |  |  |  |  |  |  |
| Luangphiphat W 2025\| -9.8128683 -12.950662 -6.6750749 |  |  |  |  |  |  |  |
| Rabiei S 2018 \| -13.259481 -17.375125 -9.1438364 |  |  |  |  |  |  |  |
| Eslamparast T 2014\| -12.39817 -16.66128 -8.135061 |  |  |  |  |  |  |  |
| Parastouei K 2020 \| -12.261445 -17.61832 -6.90457 |  |  |  |  |  |  |  |
| Rahimi F 2022 \| -13.223641 -17.43323 -9.014052 |  |  |  |  |  |  |  |
| Gorenjak M 2015 \| -12.482686 -16.538091 -8.4272816 |  |  |  |  |  |  |  |
| Cicero AFG 2020 \| -12.48787 -17.038568 -7.9371718 |  |  |  |  |  |  |  |
| Kathiya MD 2025 \| -11.462956 -15.92952 -6.9963915 |  |  |  |  |  |  |  |
| Depommier C 2020 \| -12.206058 -17.381959 -7.0301581 |  |  |  |  |  |  |  |
| -------------------+---------------------------------------------------------- |  |  |  |  |  |  |  |
| Combined \| -12.094423 -16.149073 -8.039773 |  |  |  |  |  |  |  |
| ------------------------------------------------------------------------------ |  |  |  |  |  |  |  |

# 6.2.6 Sensitive analysis of TG

**Table S6.2.6 Sensitive analysis of TG**

| ------------------------------------------------------------------------------ |  |  |  |  |  |  |  |  |
| --- | --- | --- | --- | --- | --- | --- | --- | --- |
| Study omitted \| Estimate [95% Conf. Interval] |  |  |  |  |  |  |  |  |
| -------------------+---------------------------------------------------------- |  |  |  |  |  |  |  |  |
| Luangphiphat W 2025\| -21.004554 -28.4488 -13.560307 |  |  |  |  |  |  |  |  |
| Rabiei S 2018 \| -21.857095 -29.346496 -14.367694 |  |  |  |  |  |  |  |  |
| Eslamparast T 2014\| -18.548145 -25.632959 -11.463331 |  |  |  |  |  |  |  |  |
| Parastouei K 2020 \| -18.823587 -25.919768 -11.727405 |  |  |  |  |  |  |  |  |
| Rahimi F 2022 \| -21.542811 -29.053778 -14.031845 |  |  |  |  |  |  |  |  |
| Gorenjak M 2015 \| -21.19424 -28.304623 -14.083858 |  |  |  |  |  |  |  |  |
| Cicero AFG 2020 \| -20.653116 -28.870368 -12.435865 |  |  |  |  |  |  |  |  |
| Kathiya MD 2025 \| -20.254846 -29.142051 -11.367642 |  |  |  |  |  |  |  |  |
| Wastyk HC 2023 \| -22.545342 -29.766571 -15.324114 |  |  |  |  |  |  |  |  |
| Depommier C 2020 \| -18.555776 -26.129903 -10.981649 |  |  |  |  |  |  |  |  |
| -------------------+---------------------------------------------------------- |  |  |  |  |  |  |  |  |
| Combined \| -20.513401 -27.688785 -13.338017 |  |  |  |  |  |  |  |  |
| ------------------------------------------------------------------------------ |  |  |  |  |  |  |  |  |

# 6.2.7 Sensitive analysis of LDL-C

**Table S6.2.7 Sensitive analysis of LDL-C**

| ------------------------------------------------------------------------------ |  |  |  |  |  |  |  |  |
| --- | --- | --- | --- | --- | --- | --- | --- | --- |
| Study omitted \| Estimate [95% Conf. Interval] |  |  |  |  |  |  |  |  |
| -------------------+---------------------------------------------------------- |  |  |  |  |  |  |  |  |
| Luangphiphat W 2025\| -7.3775372 -10.086191 -4.6688838 |  |  |  |  |  |  |  |  |
| Rabiei S 2018 \| -10.889919 -15.984039 -5.7957991 |  |  |  |  |  |  |  |  |
| Eslamparast T 2014\| -11.079701 -15.653897 -6.5055044 |  |  |  |  |  |  |  |  |
| Parastouei K 2020 \| -10.715465 -16.543669 -4.8872612 |  |  |  |  |  |  |  |  |
| Rahimi F 2022 \| -12.122913 -16.632123 -7.6137043 |  |  |  |  |  |  |  |  |
| Gorenjak M 2015 \| -11.013564 -15.457382 -6.5697468 |  |  |  |  |  |  |  |  |
| Cicero AFG 2020 \| -10.857995 -15.880618 -5.8353708 |  |  |  |  |  |  |  |  |
| Kathiya MD 2025 \| -10.187835 -15.189657 -5.1860123 |  |  |  |  |  |  |  |  |
| Depommier C 2020 \| -11.284712 -16.536699 -6.0327251 |  |  |  |  |  |  |  |  |
| -------------------+---------------------------------------------------------- |  |  |  |  |  |  |  |  |
| Combined \| -10.562519 -14.95793 -6.1671091 |  |  |  |  |  |  |  |  |
| ------------------------------------------------------------------------------ |  |  |  |  |  |  |  |  |

# 6.2.8 Sensitive analysis of HDL-C

**Table S6.2.8 Sensitive analysis of HDL-C**

| ------------------------------------------------------------------------------ |  |  |  |  |  |  |  |  |
| --- | --- | --- | --- | --- | --- | --- | --- | --- |
| Study omitted \| Estimate [95% Conf. Interval] |  |  |  |  |  |  |  |  |
| -------------------+---------------------------------------------------------- |  |  |  |  |  |  |  |  |
| Luangphiphat W 2025\| 1.9093261 .45915658 3.3594956 |  |  |  |  |  |  |  |  |
| Rabiei S 2018 \| 2.2739325 .33480781 4.2130573 |  |  |  |  |  |  |  |  |
| Eslamparast T 2014\| 1.1035196 .03963609 2.1674031 |  |  |  |  |  |  |  |  |
| Parastouei K 2020 \| 2.2567511 .29414599 4.2193562 |  |  |  |  |  |  |  |  |
| Rahimi F 2022 \| 2.3939674 .84515898 3.9427758 |  |  |  |  |  |  |  |  |
| Gorenjak M 2015 \| 2.0014543 .56670687 3.4362017 |  |  |  |  |  |  |  |  |
| Cicero AFG 2020 \| 1.7028181 .26026599 3.1453702 |  |  |  |  |  |  |  |  |
| Kathiya MD 2025 \| 1.9705125 .37275739 3.5682677 |  |  |  |  |  |  |  |  |
| Wastyk HC 2023 \| 2.1061186 .63097932 3.581258 |  |  |  |  |  |  |  |  |
| -------------------+---------------------------------------------------------- |  |  |  |  |  |  |  |  |
| Combined \| 1.9715374 .55391132 3.3891635 |  |  |  |  |  |  |  |  |
| ------------------------------------------------------------------------------ |  |  |  |  |  |  |  |  |

# 6.2.9 Sensitive analysis of insulin

**Table S6.2.9 Sensitive analysis of insulin**

| ------------------------------------------------------------------------------ |  |  |  |  |  |  |  |  |
| --- | --- | --- | --- | --- | --- | --- | --- | --- |
| Study ommited \| Coef. [95% Conf. Interval] |  |  |  |  |  |  |  |  |
| -------------------+---------------------------------------------------------- |  |  |  |  |  |  |  |  |
| Rabiei S 2018 \| -9.3120042 -14.295614 -4.3283948 |  |  |  |  |  |  |  |  |
| Rahimi F 2022 \| -14.422544 -20.997358 -7.8477309 |  |  |  |  |  |  |  |  |
| Depommier C 2020 \| -2.3689585 -7.134766 2.3968489 |  |  |  |  |  |  |  |  |
| Cicero AFG 2020 \| -14.45033 -21.262196 -7.638464 |  |  |  |  |  |  |  |  |
| -------------------+---------------------------------------------------------- |  |  |  |  |  |  |  |  |
| Combined \| -9.6744346 -14.660349 -4.6885206 |  |  |  |  |  |  |  |  |
| ------------------------------------------------------------------------------ |  |  |  |  |  |  |  |  |

# 6.2.10 Sensitive analysis of HOMA-IR

**Table S6.2.10 Sensitive analysis of HOMA-IR**

| ------------------------------------------------------------------------------ |  |  |  |  |  |  |  |  |
| --- | --- | --- | --- | --- | --- | --- | --- | --- |
| Study ommited \| Coef. [95% Conf. Interval] |  |  |  |  |  |  |  |  |
| -------------------+---------------------------------------------------------- |  |  |  |  |  |  |  |  |
| Yang JL 2023 \| -1.0106576 -3.4036899 1.3823747 |  |  |  |  |  |  |  |  |
| Rabiei S 2018 \| .07292296 -.36078629 .50663222 |  |  |  |  |  |  |  |  |
| Rahimi F 2022 \| -1.1338585 -4.0506747 1.7829577 |  |  |  |  |  |  |  |  |
| Cicero AFG 2020 \| -1.3106643 -3.9071875 1.285859 |  |  |  |  |  |  |  |  |
| -------------------+---------------------------------------------------------- |  |  |  |  |  |  |  |  |
| Combined \| -.85775285 -2.8458763 1.1303706 |  |  |  |  |  |  |  |  |
| ------------------------------------------------------------------------------ |  |  |  |  |  |  |  |  |
|  |  |  |  |  |  |  |  |  |

# 6.2.11 Sensitive analysis of ALT

**Table S6.2.11 Sensitive analysis of ALT**

| ------------------------------------------------------------------------------ |  |  |  |  |  |  |  |  |
| --- | --- | --- | --- | --- | --- | --- | --- | --- |
| Study ommited \| Coef. [95% Conf. Interval] |  |  |  |  |  |  |  |  |
| -------------------+---------------------------------------------------------- |  |  |  |  |  |  |  |  |
| Cicero AFG 2020 \| -.91715983 -3.7986437 1.9643241 |  |  |  |  |  |  |  |  |
| Luangphiphat W 2025\| -.67970373 -2.7356954 1.3762879 |  |  |  |  |  |  |  |  |
| Yang JL 2023 \| -1.508167 -3.5855338 .56919986 |  |  |  |  |  |  |  |  |
| Depommier C 2020 \| -2.0034985 -3.6302158 -.37678127 |  |  |  |  |  |  |  |  |
| Rahimi F 2022 \| -.72856076 -3.0227985 1.565677 |  |  |  |  |  |  |  |  |
| -------------------+---------------------------------------------------------- |  |  |  |  |  |  |  |  |
| Combined \| -1.1327945 -3.1388902 .8733013 |  |  |  |  |  |  |  |  |
| ------------------------------------------------------------------------------ |  |  |  |  |  |  |  |  |

# 6.2.12 Sensitive analysis of AST

**Table S6.2.12 Sensitive analysis of AST**

| ------------------------------------------------------------------------------ |  |  |  |  |  |  |  |  |
| --- | --- | --- | --- | --- | --- | --- | --- | --- |
| Study ommited \| Coef. [95% Conf. Interval] |  |  |  |  |  |  |  |  |
| -------------------+---------------------------------------------------------- |  |  |  |  |  |  |  |  |
| Cicero AFG 2020 \| -.72869678 -2.2361513 .77875768 |  |  |  |  |  |  |  |  |
| Depommier C 2020 \| -1.1575468 -2.7281579 .41306435 |  |  |  |  |  |  |  |  |
| Luangphiphat W 2025\| -.7313937 -1.7720542 .30926677 |  |  |  |  |  |  |  |  |
| Rahimi F 2022 \| -.83686152 -2.1320496 .4583266 |  |  |  |  |  |  |  |  |
| Yang JL 2023 \| -1.1340605 -2.0147811 -.25333984 |  |  |  |  |  |  |  |  |
| -------------------+---------------------------------------------------------- |  |  |  |  |  |  |  |  |
| Combined \| -.94759633 -2.0040138 .10882113 |  |  |  |  |  |  |  |  |
| ------------------------------------------------------------------------------ |  |  |  |  |  |  |  |  |

# Supplementary 7 Confunnel plots for human and animal studies

# 7.1 Animal studies

# 7.1.1 Confunnel plots for HbA1c

**Figure S7.1.1 Confunnel plots for HbA1c**

# 7.1.2 Confunnel plots for BW

**Figure S7.1.2 Confunnel plots for BW**

# 7.1.3 Confunnel plots for LDL-C

**Figure S7.1.3 Confunnel plots for LDL-C**

# 7.1.4 Confunnel plots for HDL-C

**Figure S7.1.4 Confunnel plots for HDL-C**

# 7.1.5 Confunnel plots for insulin

**Figure S7.1.5 Confunnel plots for insulin**

# 7.1.6 Confunnel plots for HOMA-IR

**Figure S7.1.6 Confunnel plots for HOMA-IR**

# 7.1.7 Confunnel plots for OGTT 0h blood glucose

**Figure S7.1.7 Confunnel plots for OGTT 0h blood glucose**

# 7.1.8 Confunnel plots for OGTT 2h blood glucose

**Figure S7.1.8 Confunnel plots for OGTT 2h blood glucose**

# 7.1.9 Confunnel plots for CRP

**Figure S7.1.9 Confunnel plots for CRP**

# 7.1.10 Confunnel plots for IL-6

**Figure S7.1.10 Confunnel plots for IL-6**

# 7.1.11 Confunnel plots for IL-1β

**Figure S7.1.11 Confunnel plots for IL-1β**

# 7.1.12 Confunnel plots for TNF-α

**Figure S7.1.12 Confunnel plots for TNF-α**

# 7.1.13 Confunnel plots for LPS

**Figure S7.1.13 Confunnel plots for LPS**

# 7.1.14 Confunnel plots for FFA

**Figure S7.1.14 Confunnel plots for FFA**

# 7.1.15 Confunnel plots for leptin

**Figure S7.1.15 Confunnel plots for leptin**

# 7.1.16 Confunnel plots for ALT

**Figure S7.1.16 Confunnel plots for ALT**

# 7.1.17 Confunnel plots for AST

**Figure S7.1.17 Confunnel plots for AST**

# 7.2 Human studies

# 7.2.1 Confunnel plots for BW

**Figure S7.2.1 Confunnel plots for BW**

# 7.2.2 Confunnel plots for WC

**Figure S7.2.2 Confunnel plots for WC**

# 7.2.3 Confunnel plots for LDL-C

**Figure S7.2.3 Confunnel plots for LDL-C**

# 7.2.4 Confunnel plots for HDL-C

**Figure S7.2.4 Confunnel plots for HDL-C**

# 7.2.5 Confunnel plots for insulin

**Figure S7.2.5 Confunnel plots for insulin**

# 7.2.6 Confunnel plots for HOMA-IR

**Figure S7.2.6 Confunnel plots for HOMA-IR**

# 7.2.7 Confunnel plots for ALT

**Figure S7.2.7 Confunnel plots for ALT**

# 7.2.8 Confunnel plots for AST

**Figure S7.2.8 Confunnel plots for AST**
